
JOURNAL INFORMATION
==============================
NLM Title Abbreviation: Artery Res
Journal ID: Artery Res
NLM Journal ID: 101310251

Artery Research

ISSN: 1872-9312
EISSN: 1876-4401

ARTICLE INFORMATION
==============================
PMCID: PMC9904244
PMID: 36776700
DOI: 10.1007/s44200-022-00028-8
Article ID: 28
Article version: 1
Subjects: Abstracts

Selected Abstracts from Artery 22

Wednesday 19 – Saturday 22 October 2022, Centre de Congrès Prouvé, Nancy, France

Electronic publication date: 2023 Feb 7
Print publication date: 2023
Volume: 29
Issue: Suppl 1
First page: 1
Last page: 50
Copyright: © The Author(s) 2023
License: Open AccessThis article is licensed under a Creative Commons Attribution 4.0 International License, which permits use, sharing, adaptation, distribution and reproduction in any medium or format, as long as you give appropriate credit to the original author(s) and the source, provide a link to the Creative Commons licence, and indicate if changes were made. The images or other third party material in this article are included in the article's Creative Commons licence, unless indicated otherwise in a credit line to the material. If material is not included in the article's Creative Commons licence and your intended use is not permitted by statutory regulation or exceeds the permitted use, you will need to obtain permission directly from the copyright holder. To view a copy of this licence, visit http://creativecommons.org/licenses/by/4.0/.
License URL: https://creativecommons.org/licenses/by/4.0/

issue-copyright-statement: © The Author(s) 2023

==============================

*Artery Research* (2023) 29:S1–S50
https://doi.org/10.1007/s44200-022-00028-8

Artery Research

ABSTRACTS

Open Access

### Selected Abstracts from Artery 22

Wednesday 19 – Saturday 22 October 2022, Centre de Congrès Prouvé, Nancy, France

### Oral presentations

### O.1
### Microstructural deterioration drives progressive functional loss in Marfan syndrome aneurysms

**Cristina Cavinato**[1], David Lee[1], Marcos Latorre[2], Minghao Chen[3], Dar Weiss[1], María Jesús Ruiz-Rodríguez[4], Martin A. Schwartz[3,5], Jay D. Humphrey[1,5]

[1]Department of Biomedical Engineering, Yale University, New Haven, USA, [2]Center for Research & Innovation in Bioengineering, Valencia Polytechnic University, Valencia, Spain, [3]Cardiovascular Research Center, Yale School of Medicine, New Haven, USA, [4]Centro Nacional de Investigaciones Cardiovasculares Carlos III, Madrid, Spain, [5]Vascular Biology and Therapeutics Program, Yale School of Medicine, New Haven, USA

**Background:** Marfan Syndrome is a primary cause of thoracic aortic aneurysms; it arises from dysfunctional fibrillin-1, which normally stabilizes elastic fibers and promotes smooth muscle mechano-sensing of the matrix. Despite significant advancements, clear correlations between microstructural integrity and aortic functionality remain wanting.
**Methods:** Age-matched wild-type, Fbn1C1041G/+[1], and Fbn1mgR/mgR [2] mice represented three stages of disease severity. Experiments quantified specimen-specific thoracic aortopathy in terms of: (1) mechanical metrics from ex vivo biaxial testing that were described by a four-fiber family hyperelastic model [3]; (2) microstructural metrics[4] from ex vivo multiphoton microscopy including elastin porosity, density, and engagement of collagen fibers and cells; (3) cardiac function from in vivo ultrasound and μCT imaging. Material properties were incorporated within a mechanobiologically equilibrated constrained mixture model of arterial growth and remodeling (G&R) [5]. The analysis assessed long-term impacts of locally compromised elastin integrity, cellular mechanosensing and mechanoregulation, collagen turnover, and endothelial function on disease progression through perturbations to the initial homeostatic state.
**Results**: Aortic dilatation correlated strongly with key mechanical metrics of compromised aortic functionality as well as with elastin defects, collagen remodeling, and altered cellular function. Variable dilatations at a given age reflected a "pseudo-time" of progressive deterioration consistent with a progressive anoikis. The G&R model reproduces the same trends in aortic dilatation, stored energy, and circumferential stiffness with increasing losses of elastic fiber integrity. The progressive deterioration of elastic fibers and mechano-sensing appear to be primary drivers of aberrant tissue remodeling and associated dilatation in the Marfan aorta, which is characterized by progressive stiffening.

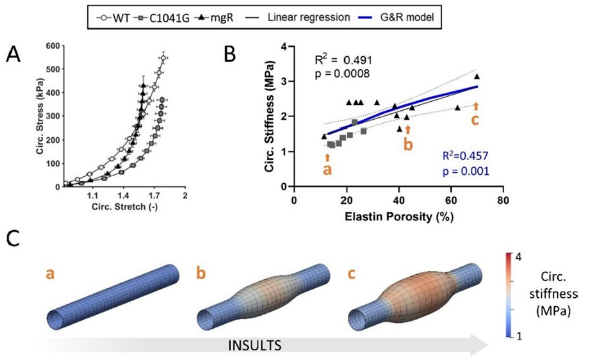

Biaxial stress-stretch curves (A) and circumferential stiffness vs. elastin porosity (B) for the ascending thoracic aorta of the analyzed Marfan mice. Dilatation and circumferential stiffness from the G&R model (C).

**References**

[1] Milewicz DM, et al. Marfan syndrome. Nat Rev Dis Primers. 2021;7(1):64.
[2] Pereira L, et al. Pathogenetic sequence for aneurysm revealed in mice underexpressing fibrillin-1. PNAS. 1999;96(7):3819–23.
[3] Judge DP, et al. Evidence for a critical contribution of haploinsufficiency in the complex pathogenesis of Marfan syndrome. J Clin Invest. 2004;114(2):172–81.
[4] Cavinato C, Chen M, Weiss D, Ruiz-Rodríguez MJ, Schwartz MA, Humphrey JD. Progressive Microstructural deterioration dictates evolving biomechanical dysfunction in the Marfan aorta. Front Cardiovascular Medicine. 2021;8:1904.
[5] Latorre M, Humphrey JD. Numerical knockouts–In silico assessment of factors predisposing to thoracic aortic aneurysms. PLoS Comput Biol. 2020;16(10).

**Keywords**: Marfan syndrome, Biaxial mechanics, Microstructure, Growth and remodeling, Aneurysms

© The Author(s) 2022. **Open Access** This article is licensed under a Creative Commons Attribution 4.0 International License, which permits use, sharing, adaptation, distribution and reproduction in any medium or format, as long as you give appropriate credit to the original author(s) and the source, provide a link to the Creative Commons licence, and indicate if changes were made. The images or other third party material in this article are included in the article's Creative Commons licence, unless indicated otherwise in a credit line to the material. If material is not included in the article's Creative Commons licence and your intended use is not permitted by statutory regulation or exceeds the permitted use, you will need to obtain permission directly from the copyright holder. To view a copy of this licence, visit http://creativecommons.org/licenses/by/4.0/.

### O.2

### Impaired β2-adrenergic endothelium-dependent vasodilation is reversed by phosphodiesterase inhibition in patients previously hospitalized with COVID-19

**Dr. Luca Faconti**[1], Ms Bushra Farikh[1], Dr. Ryan J McNally[1], Dr Sally Brett[1], Prof. Philip J Chowienczyk[1]

[1]King's College London, London, United Kingdom

**Background:** Endothelial dysfunction may underlie many of the complications of COVID-19 [1]. The pulse wave response to salbutamol (PWRS)—change in the augmentation index, AIx- provides a means to assess endothelial vasodilator function mediated through the nitric oxide-cyclic guanosine monophosphate pathway (NO-cGMP) [2,3]. Here we aim to determine whether PWRS is abnormal in patients recovered from COVID-19.

**Methods**: We examined PWRS in subjects previously hospitalized with COVID-19, those recovered from mild symptoms and seronegative controls (absence of SARS-CoV-2-antibodies) with similar risk factors for cardiovascular disease. In a sub-sample, we also assessed the response in the presence and absence of the phosphodiesterase type 5 inhibitor sildenafil which inhibits the breakdown of cGMP.

**Results**: 101 subjects (60 men) aged 47.8 ± 14.1 (mean ± SD) years of whom 33 were previously hospitalized with COVID-19 were recruited. Inhaled salbutamol reduced AIx in controls (n = 34) and those recovered from mild symptoms of COVID-19 (n = 34) but produced an increase in AIx in those previously hospitalized: mean change [95% confidence interval] − 2.85 [− 5.52, − 0.188] %, − 2.32 [− 5.17, 0.54] %, and 3.03 [0.06, 6.00] % for controls, those recovered from mild symptoms and those previously hospitalised respectively (P = 0.001). In a sub-sample (n = 22), sildenafil enhanced the response to salbutamol (change in AIx 0.05 [− 2.15, 2.24] vs. − 3.96 [− 7.01, − 2.18], P = 0.006) with no significant difference between hospitalized (n = 12) and non-hospitalized subjects (n = 10).

**Conclusions**: In patients previously hospitalized with COVID-19, there is long-lasting impairment of endothelial function which can be ameliorated by sildenafil.

**References**

[1] Libby P, Lüscher T. COVID-19 is, in the end, an endothelial disease. Eur. Heart J. 2020;41:3038–3044.

[2] Chowienczyk PJ, Kelly RP, MacCallum H, Millasseau SC, Andersson TLG, Gosling RG, Ritter JM, Änggård EE. Photoplethysmographic assessment of pulse wave reflection: Blunted response to endothelium-dependent beta2-adrenergic vasodilation in type II diabetes mellitus. J. Am. Coll. Cardiol. 1999;34:2007–2014.

[3] Wilkinson IB, Hall IR, Maccallum H, Mackenzie IS, Mceniery CM, Arend BJ Van Der, Shu Y, Mackay LS, Webb DJ, Cockcroft JR. Clinical Evaluation of a Noninvasive, Widely Applicable Method for Assessing Endothelial Function. 2002;

**Keywords**: COVID-19, Endothelium, AIx

### O.3

### How does mechanical stress affect gene expression in human aortic smooth muscle cells?

Mme Claudie Petit[1], Amira Ben Hassine[1], Mireille Thomas[2], Alain Guignandon[2], **Pr. Stéphane Avril**[1]

[1]Mines Saint-Etienne, Université de Lyon, INSERM, U 1059 SAINBIOSE, Saint-Etienne, France, [2]Université Jean Monnet, Université de Lyon, INSERM, U 1059 SAINBIOSE, Saint-Etienne, France

**Background:** SMC modulate their phenotype in response to environmental conditions, as in for instance ascending thoracic aortic aneurysms (ATAA) (1–3). It was previously shown that missensing of mechanical stimuli plays a major role in ATAA (2,4). Nevertheless, there is a pressing need to better quantify the mechanobiological behaviour of SMCs.

**Methods**: To address this need, we applied mechanical stimulations on human aortic SMC in culture at passage 6–7, by using the Flexcell tension system (Fig. 1A). We tried different durations of stimulation (24 h, 48 h, 72 h, and 100 h) versus unstimulated control. We chose 7 genes, coding for contractile proteins of the cytoskeleton (Fbn1, ACTA2), extracellular matrix components (Coll1A1, LAMA5), or involved in activation/regulation of traction forces (TGFBR1, MYLK), and in cell differentiation (TAGLN). The expression of these genes was quantified with qPCR analysis, relatively to a reference gene (HPRT) (Fig. 1B).

**Results**: We observed that: 1. From 72 h of stimulation, the difference between stimulated and control groups is the most significant; 2. For the majority of the genes, their expression decreases with the stimulation; 3. The basal medium enhances α-SMA production. Nevertheless, due to the low quantity of RNA available, we had to repeat cell stimulation, and reach n = 3 repetitions for each group during the qPCR analysis for more accurate results.

**Conclusions**: We were able to quantify SMC mechanosensitivity and mechanotransduction. AoSMC seem to modulate their gene expression after 72 h of stimulation. As future work, we would like to investigate the influence of intercellular signaling under stimulation.

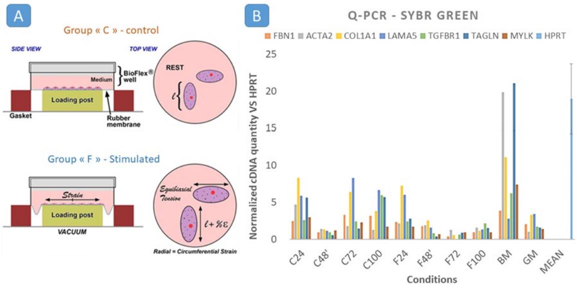

(A) Control group and stimulated group with Flexcell tension system. (B) Results of the qPCR analysis on all groups for 7 genes (cDNA quantity, relatively to a reference gene (HPRT)).

**References**

1. Michel J-B, Jondeau G, Milewicz DM. From genetics to response to injury: vascular smooth muscle cells in aneurysms and dissections of the ascending aorta. Cardiovasc Res. 2018;114(4):578–89.
2. Milewicz DM, Trybus KM, Guo D, Sweeney HL, et al. Altered Smooth Muscle Cell Force Generation as a Driver of Thoracic Aortic Aneurysms and Dissections. Arteriosclerosis, Thrombosis, and Vascular Biology. 2016;116:303229.
3. Liu M, Gomez D. Smooth Muscle Cell Phenotypic Diversity. Arterioscler Thromb Vasc Biol. 2019;39(9):1715–1723.
4. Humphrey JD, Schwartz MA, Tellides G, Milewicz DM. Role of Mechanotransduction in Vascular Biology: Focus on Thoracic Aortic Aneurysms and Dissections. Circulation Research. 2015;116(8):1448–61.

**Keywords**: Cell biomechanics, Epigenetics, Mechanical stimulation, qPC

### O.4

### Pentosan polysulfate, an aggrecanase inhibitor modulates arterial stiffness in spontaneously hypertensive rats

Miss Aleksandra Klosinska[1], **Dr Keith Siew**[2], Mr Tao Luo[3], Mrs Nichola Figg[4], Mrs Sarah Cleary[1], Dr Isam Sharif[5], Mr Colin Williams[5], Professor Ian Wilkinson[1], Professor Michael Sutcliffe[3], Professor Kevin O'Shaughnessy[1], Dr Y Yasmin[1]

[1]University of Cambridge, Department of Medicine, EMIT Division, Cambridge, United Kingdom, [2]University College London, Department of Renal Medicine, London, United Kingdom, [3]University of Cambridge,

Department of Engineering, Cambridge, United Kingdom, [4]University of Cambridge, Department of Medicine, Cardiovascular Division, Cambridge, United Kingdom, [5]Covance CRS Limited, Huntingdon, United Kingdom

**Background:** Arterial stiffness is an independent predictor of all-cause and cardiovascular mortality in many populations. Our recent research showed that loss of aggrecan integrity associates with age-related arterial stiffening (ARAS) in humans1, and others have shown that inhibition of ADAMTS/aggrecanase enzymes, which degrade aggrecan, improves cardiac function 2. Currently, there are no drugs that specifically target arterial stiffening in humans. We hypothesise that ADAMTS inhibitor, Pentosan Polysulfate (PPS), represents an attractive molecule that can be repurposed as a first-in-class drug treatment for ARAS.

**Methods**: We performed an in vivo pharmacological experiment using 15wk-old spontaneous hypertensive male rats (SHR) that were administered either PPS or vehicle control (n = 7 per group) subcutaneously 3 times per week for 4 weeks. Animals were sacrificed and fully intact aortae including blood, cartilage, etc. were harvested and stored at −800 °C. Arterial wall thickness, stress-strain and failures stress were measured, and tensile elasticity calculated ex vivo.
**Results**: Preliminary analysis showed that PPS significantly reduced aortic wall thickening normally associated with arterial stiffening in hypertension (Vehicle 225 ± 6 μm vs 204 ± 6 μm, p = 0.0143). PPS also decreased aortic stiffening significantly at supraphysiological pressures in treated rats, and treated rats had a higher failure stress relative to vehicle controls.
**Conclusions**: This proof-of-principle study demonstrated that an aggrecanase inhibitor can modulate aortic stiffness markers in SHR, but the short treatment period may not be adequate to reveal clinically significant differences. Further longitudinal studies are therefore, needed to establish if longer exposure to PPS can reduce aortic stiffness at clinically significant levels in older animals.

**References**
[1] Yasmin et al. The matrix proteins aggrecan and fibulin-1 play a key role in determining aortic stiffness. Sci Rep. 2018 Jun 4;8(1):8550. https://doi.org/10.1038/s41598-018-25851-5.
[2] Vistnes M. et al. Penstisan polysulfate decreases myocardial expression of the extracellular matrixenzyme ADAMTS4 and improves cardiac function in vivo rates subjected to pressure overload by aortoc banding. PLOS One 2014: 9, e8923. https://doi.org/10.1371/journal.pone.0089621.

**Keywords**: Arterial stiffness, Aggrecan, Pentosan polysulfate, Spontaneous hypertensive rats

### O.5

### Radial-digital pulse wave velocity: stiffness of small conduit arteries increases after nitroglycerin administration

**Dr Catherine Fortier**[1,2], Charles-Antoine Garneau[1], Mathilde Paré[1], Dr Hasan Obeid[1], Nadège Côté[1], Karine Duval[1], Dr Rémi Goupil[2], Dr Mohsen Agharazii[1]

[1]Chu De Québec Research Center - Université Laval, Québec, Canada, [2]CIUSSS NIM- Sacré Coeur de Montréal, Montréal, Canada

**Background:** The alteration of the physiological stiffness gradient within larger vessels would increase the transmission of greater pulsatility to the microcirculation, thus explaining the damage to the pressure-sensitive organs. We aimed to describe the response of small conduit arteries of the hand (radial-digital PWV, rd-PWV) (1) following the pharmacological alteration of the stiffness gradient using nitroglycerin. (2).
**Methods**: Simultaneous application of piezoelectric sensors (Complior) at the level of the carotid (c), the radial artery (r) and the tip of the index finger (d) was used to calculate the rd-PWV, before and 4 min after a sublingual administration of 0.4 mg of NTG. Changes in arterial stiffness pre-post NTG and comparisons between two groups whether healthy adults (controls, n = 36) or patients with moderate chronic kidney disease (CKD, n = 30) were analyzed respectively with paired or independent samples t-tests.
**Results**: Despite similar cd-PWV and cr-PWV, rd-PWV at baseline was significantly lower in CKD than in controls (3.6 ± 1.4 m/s vs. 4.5 ± 1.8 m/s, p = 0.024), but this difference faded (p = 0.145) after adjustment for age. Post NTG, brachial blood pressure decreased and heart rate increased similarly between groups. Rd-PWV increased in controls (from 4.62 ± 1.49 m/s to 5.94 ± 2.29 m/s, p < 0.001) and in CKD (from 3.71 ± 1.60 m/s to 5.18 ± 2.12 m/s, p < 0.001), in a similar extent (interaction p = 0.111). However, cd-PWV significantly increased post-NTG only in the CKD group (p = 0.009).
**Conclusions**: This technique, adapted by our team for small conduit arteries, may broaden our understanding of the consequences of the inversion of the stiffness gradient.

**References**
1. Obeid H, Fortier C, Garneau CA, Pare M, Boutouyrie P, Bruno RM, et al. Radial-digital pulse wave velocity: a noninvasive method for assessing stiffness of small conduit arteries. Am J Physiol Heart Circ Physiol. 2021;320(4):H1361-H9.
2. Fortier C, Garneau CA, Pare M, Obeid H, Cote N, Duval K, et al. Modulation of Arterial Stiffness Gradient by Acute Administration of Nitroglycerin. Front Physiol. 2021;12:774056.

**Keywords:** Chronic kidney disease, Nitroglycerin, Arterial stiffness gradient, Small conduit arteries

### O.6

### Characterization of internal jugular vein region-specific distension during progressive volume loading

**Mr Jeremy N. Cohen**[1], Mr Eric T. Hedge[1,2], Dr Danielle K. Greaves[2], Dr Andrew D. Robertson[1,2], Dr Lonnie G. Petersen[3], Dr Jason S. Au[1]

[1]Department of Kinesiology and Health Sciences, University Of Waterloo, Waterloo, Canada, [2]Schlegel-University of Waterloo Research Institute for Aging, University of Waterloo, Waterloo, Canada, [3]Department of Aeronautics and Astronautics, Massachusetts Institute of Technology, Cambridge, United States of America

**Background:** The internal jugular vein (IJV) is highly compliant with roles in intracranial blood flow and pressure regulation. The dynamic and variable nature of venous flow, especially during changes in gravitational stress, leads to non-linear geometry. However, the impact of this irregular anatomy on IJV distension during progressive volume loading is not understood and may contribute to adverse flow profiles, a component of Virchow's triad for thrombotic risk. We characterized IJV 3D shape and volume expansion during progressive head-down tilt (HDT), a microgravity analogue.
**Methods**: We recruited 5 healthy, young adults (2 females, 25 ± 4 year, 168 ± 8 cm, 68 ± 15 kg). Using an ultrasound probe tracked in 3D space, we captured right IJV cross-sectional area (CSA) from clavicle to mandible. Progressive cephalad fluid shift was achieved by HDT at 0°, −6°, −15°, and −30°, each held for 5 min. CSA were traced at 0.3 cm intervals from caudal to cranial and vein volume calculated by cylindrical CSA.
**Results**: Progressive HDT significantly altered IJV distension, demonstrating stepwise effects on average CSA (0.89 ± 0.4cm$^2$, 1.26 ± 0.6cm$^2$, 1.68 ± 0.7cm$^2$, 2.02 ± 1cm$^2$; P = 0.003, $\eta^2$ = 0.67) and total volume (4.0 ± 1.5 mL, 5.8 ± 3.3 mL, 7.6 ± 4.2 mL, 9.3 ± 5.4 mL; P = 0.007, $\eta^2$ = 0.63) through HDT conditions 0°, −6°, −15°, −30°, respectively. Caudal regions displayed greater distension capacity compared to cranial across conditions (P < 0.001).
**Conclusion**: Our precise 3D volume measures demonstrate the IJV can accommodate significant fluid shifts through a large range in distension ability, beyond stimuli mimicking microgravity. Irregular expansion patterns in the caudal regions may lend to flow abnormalities and requires investigation to ascertain prognostic value of IJV geometry on thrombotic risk.

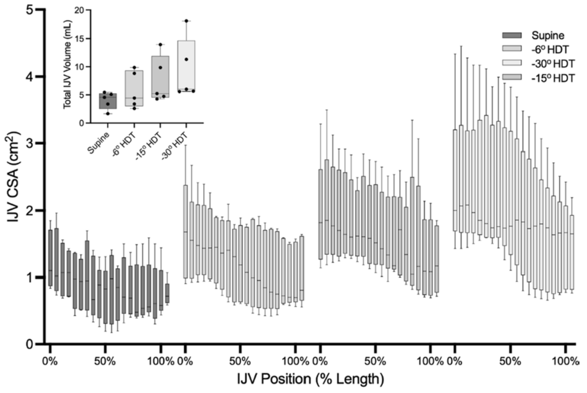

**Fig. 1** Internal jugular vein cross-sectional area from caudal to cranial regions normalized to individual neck length (A) and total cylindrical vein volume during progressive head-down tilt (B)

**Keywords**: Microgravity, Venous, 3D

### O.7

### New approach in the design of a human tissue engineered vascular graft and preliminary studies in arterial bypass models in the pig

Dr Adrien Fayon[1], Mrs Deborah Helle[1], Dr Véronique Regnault[2], Dr Marc Ponçot[3], Pr Isabelle Royaud[3], Pr Jean-Pablo Maureira[2,4], Dr Dan Pan[4], **Dr Caroline Gaucher**[5], Pr Patrick Menu[1], Dr Reine El Omar[1]

[1]Université de Lorraine, CNRS, IMoPA, F-54000 Nancy, France, [2]Université de Lorraine, Inserm, DCAC, F-54000 Nancy, France, [3]Université de Lorraine, CNRS, IJL, F-54000 Nancy, France, [4]Université de Lorraine, CHRU-Nancy, Service de Chirurgie Cardiovasculaire, F-54000 Nancy, France, [5]Université de Lorraine, CITHEFOR, F-54000 Nancy, France

**Background:** Arterial bypass surgery of small-caliber vessels using synthetic vascular prostheses remains inefficient as they promote thrombosis probably due to a low or non-functionalization of their internal surface(1). As a potential therapeutic alternative, we have developed a human cellularized TEVG (tissue-engineered vascular graft) whose components are all derived from the umbilical cord.
**Methods**: Decellularized umbilical arteries were coated at their luminal surface with an extracellular matrix extracted from Wharton's jelly and then cellularized by mesenchymal stromal cells derived also from WJ. The luminal coating and cellularization were optimized by an innovative "inside-out" method allowing easy access to the luminal surface. TEVG hemocompatibility (Thrombin Generation Assay) and mechanical properties (burst pressure test, stress rupture test, dynamic mechanical analysis) were evaluated. Preliminary in vivo implantations were conducted in pigs for coronary or femoral arterial bypass.
**Results**: The "inside-out" method, which did not impact mechanical properties of the TEVG, allowed a homogeneous cellularization of arteries luminal surface confirming its adaptability to a vascular context. Hemocompatibility assays showed that the TEVG behaves as a native blood vessel due to full cell covering of the luminal surface. The TEVG was successfully implanted in a coronary artery bypass model ($n = 1$), and was well tolerated, colonized and remain patent for 2 weeks post-implantation in a femoral replacement model ($n = 1$).
**Conclusion**: Our TEVG is an allogeneic therapeutic solution offering a ready-to-use graft that may supply a tissue bank and which can be grafted by minimally invasive robotic techniques avoiding an invasive surgery, having beneficial societal and economic impacts.

**Reference**

Fayon A, Menu P & El Omar, R. Cellularized small-caliber tissue-engineered vascular grafts: looking for the ultimate gold standard. npj Regen Med. 2021 Aug (6), 46

**Keywords**: Tissue-engineered vascular graft, Human umbilical cord, Inside-out, Artery bypass models, Vascular Tissue Engineering

### O.8

### Inhibition of atherosclerotic plaque calcification by Omega-3 polyunsaturated fatty acids through the resolvin E1 receptor ChemR23

PhD Gonzalo Artiach[1], PhD Andres Laguna-Fernandez[1], PhD Miguel Carracedo[1], **PhD Hildur Arnardottir**[1], Prof. Magnus Bäck[1,2]

[1]Department of Medicine, Karolinska Institutet, Stockholm, Sweden, [2]Theme Heart and Vessels, Karolinska University Hospital, Stockholm, Sweden

**Background:** The immune cell response in atherosclerotic plaques is characterized by an impaired resolution of inflammation (1). Resolvin E1 (RvE1), a specialized pro-resolving lipid mediator derived from omega-3 polyunsaturated fatty acids (PUFA) has been shown to play a critical role in atherosclerosis by promoting the resolution of the inflammation (2). The aim of the present study was to unravel the role of omega-3 PUFA, RvE1 and the RvE1 receptor ChemR23 in the process of atherosclerotic plaque calcification.
**Methods**: Fat-1 transgene (Fat-1tg), which enables the endogenous production of n-3 PUFA, was inserted in apolipoprotein E (ApoE)-deficient mice, in combination or not with genetic deletion of ChemR23. Calcification was assessed by Alizarin Red staining and macrophage markers were assessed by immunohistochemistry in aortic root sections.
**Results**: Our results show that 72 week old Fat-1tg × Apoe-/- mice developed less atherosclerotic plaque calcification compared with Apoe-/- mice (0–3% vs 4–8%, $p < 0.001$). Moreover, deletion of ChemR23 enhanced atherosclerotic plaque calcification (4–13% vs. 4–8%, $p < 0.001$), and this effect was not reversed by the presence of Fat-1tg (4–14% vs. 4–8%, $p < 0.001$). Furthermore, the Fat-1tg × Apoe-/- mice had significantly higher expression of the M2 macrophage marker Arg1 compared with Apoe-/- mice (17.4 ± 2.5% vs 5.1 ± 1.5%; $p < 0.0001$), which was reversed by genetic deletion of ChemR23 (5.0 ± 1.1% vs. 17.4 ± 2.5%; $p < 0.0001$ vs Fat-1tg × Apoe-/- mice).
**Conclusion**: These results suggest that the beneficial effects of Fat-1tg were mediated through ChemR23. Hence, omega-3 PUFA may have a therapeutic potential for reducing atherosclerotic plaque calcification through RvE1-signaling by means of ChemR23.

**References**

1. Bäck M, Yurdagul A, Tabas I, Öörni K and Kovanen PT. Inflammation and its resolution in atherosclerosis: mediators and therapeutic opportunities. Nat Rev Cardiol. 2019 Jul;16(7):389–406.
2. Carracedo M, Artiach G, Arnardottir H and Bäck M. The resolution of inflammation through omega-3 fatty acids in atherosclerosis, intimal hyperplasia, and vascular calcification. Semin Immunopathol. 2019;41:757–766.

**Keywords**: Atherosclerosis, Calcification, Omega-3, Resolvin

### O.9

### Reduced Micromechanical Stiffness of Large Diameter Abdominal Aortic Aneurysm (AAA) Wall Tissue

Martin Hossack[2], Robert Fisher[2], Francesco Torella[2], Jillian Madine[1], **Riaz Akhtar**[1]

[1]University of Liverpool, United Kingdom, [2] Liverpool University Hospitals NHS Foundation Trust, United Kingdom

**Introduction:** Use of a maximum diameter threshold as the sole indicator for aneurysm repair risks rupture during surveillance in higher-risk cases, and unnecessary repair in others [1]. Here, we characterised the micromechanical properties of aneurysmal aortic tissues with the aim of identifying high-risk cases and directing specific management.

**Methods**: Full thickness anterior aortic wall tissue samples were harvested from 16 patients undergoing repair of degenerative AAA. Nanoindentation was used to determine the shear storage modulus (G′). We performed indentations on tissue cross-sections in 3 layers (inner, middle, outer). At least 4 samples were tested from each patient. In total, there were 102 samples (1269 indentations). We stratified micromechanical findings according to maximum transverse diameter (MTD), established through interrogation of preoperative contrast-enhanced CT scans.

**Results & Discussion**: Aortic wall tissue demonstrated a pattern of reducing stiffness from the inner to middle (median 31.5 kPa vs 24.4 kPa, $P < 0.05$) and middle to outer layers (24.4 kPa vs 13.1 kPa, $P < 0.05$). Wall stiffness increased as MTD increased from 50–59 mm to 60–69 mm (median 20.7 kPa vs 29.5 kPa, $P < 0.05$). At 70-79 mm, wall stiffness reduced (median 22 kPa, NS), and reduced further as MTD exceeded 80 mm (median 19.6 kPa, $P < 0.05$) (Fig. 1). The mechanical properties of vascular tissues depend largely on the extracellular matrix. A reduced G′, observed in larger diameter aneurysms may indicate a failure in the collagen network, predisposing to rupture.

**Conclusion**: At higher MTD, AAA wall loses stiffness at larger diameters. The work can be translated to identify individuals with high-risk AAA.

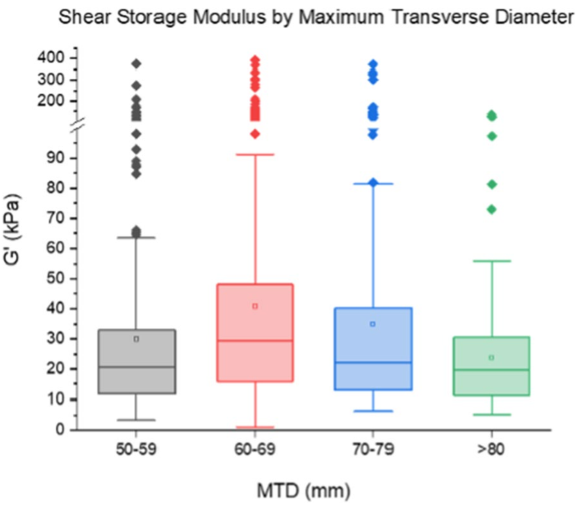

**Fig. 1** Shear storage modulus shown as a function of maximum transverse diameter

**Reference**

1.Polzer, S., Gasser, T.C., Vlachovský, R., Kubíček, L., Lambert, L., Man, V., Novák, K., Slažanský, M., Burša, J. and Staffa, R., 2020. Biomechanical indices are more sensitive than diameter in predicting rupture of asymptomatic abdominal aortic aneurysms. Journal of vascular surgery, 71(2), pp.617–626.

**Keywords**: abdominal aortic aneurysms, maximum diameter, biomechanics, vascular

### O.10

### Reservoir-wave parameters and cardiovascular prediction: Analysis of the population-based CARTaGENE cohort

**Dr Louis-Charles Desbiens**[1,4], Dr Catherine Fortier[2,3], Dr Annie-Claire Nadeau-Fredette[1,4], Dr François Madore[2,4], Dr Bernhard Hametner[5], Dr Siegfried Wassertheurer[5], Dr Mohsen Agharazii[3,6], Dr Rémi Goupil[2,4]

[1]Hôpital Maisonneuve-Rosemont, Montréal, Canada, [2]Hôpital du Sacré-Coeur de Montréal, Montréal, Canada, [3]CHU de Québec—Université Laval, Québec, Canada, [4]Université de Montréal, Montréal, Canada, [5]AIT Austrian Institute of Technology, Vienna, Austria, [6]Université Laval, Québec, Canada

**Background:** The reservoir-wave concept hypothesizes that blood pressure is the sum of a reservoir and an excess pressure. Nevertheless, the clinical association of reservoir-wave parameters with cardiovascular outcomes remains controversial.

**Methods**: We studied individuals aged between 40 and 69 from the CARTaGENE cohort (Canada). Radial waveforms were measured with aplanation tonometry (SphygmoCor). They were transformed to central waveforms using generalized transfer functions and used to generate reservoir parameters (Reservoir pressure [RP], Reservoir pressure integral [RPI], Excess pressure [XSP], Excess pressure integral [XSPI], Systolic rate constant [SC], Diastolic rate constant [DC], Optimized asymptotic pressure [PInf]). Major adverse atherosclerotic events (MACE: cardiovascular death, stroke, myocardial infarction) during a 10-year follow-up were obtained using medico-administrative databases. Associations of reservoir parameters with MACE were derived using crude and fully adjusted Cox models. Incremental predictive performance over the ASCVD score (atherosclerotic cardiovascular disease score; using revised pooled cohort equations) for each reservoir parameter was displayed using c-statistic improvement and continuous net reclassification indexes (NRI).

**Results**: From 17,629 individuals, 2327 had a MACE during the follow-up. All reservoir parameters were significantly higher in patients who experienced a MACE. After full adjustment, RP, XSPI and DC were associated with increased MACE incidence (Table). Spline analysis did not reveal any non-linear relationships between reservoir parameters and MACEs. When added to the ASCVD prediction score, XSP and DC significantly improved c-statistics while RP, XSPI, DC and PInf led to a significant net reclassification improvement.

**Conclusion**: Reservoir parameters, especially the diastolic rate constant, improve cardiovascular prediction in a population-based cohort.

Table

| Parameter | Hazard ratios (95% CI) | | Predictive statistics (95% CI) | |
|---|---|---|---|---|
| | Crude | Fully adjusted | C-Statistic improvement | Net reclassification index |
| **RP** | 1.30 (1.26-1.35)* | 1.10 (1.02-1.18)* | -0.02 (-0.07, 0.11) | 0.049 (0.006, 0.093)* |
| **RPI** | 1.19 (1.15-1.24)* | 1.02 (0.96-1.09) | 0.00 (-0.05, 0.06) | 0.028 (-0.016, 0.071) |
| **XSP** | 1.27 (1.23-1.31)* | 1.04 (0.99-1.09) | 0.02 (0.00, 0.03)* | 0.012 (-0.031, 0.056) |
| **XSPI** | 1.35 (1.30-1.39)* | 1.09 (1.04-1.15)* | 0.01 (-0.12, 0.14) | 0.064 (0.020, 0.108)* |
| **DC** | 1.17 (1.14-1.20)* | 1.05 (1.01-1.09)* | 0.17 (0.04, 0.30)* | 0.076 (0.033, 0.120)* |
| **SC** | 1.09 (1.05-1.13)* | 1.01 (0.97-1.05) | 0.03 (-0.04, 0.10) | 0.035 (-0.009, 0.078) |
| **PInf** | 1.05 (1.01-1.09)* | 0.99 (0.94-1.04) | 0.08 (-0.10, 0.25) | 0.079 (0.035, 0.122)* |

Hazard ratios are presented for one standard deviation increase. Fully adjusted models include: age, sex, race, body mass index, active smoking, diabetes, total cholesterol, HDL cholesterol, eGFR, heart rate, statin, brachial SBP, pre-existing cardiovascular disease, antihypertensive use

* Denotes a *p*-value $< 0.05$

### O.11

### Histomorphometric analysis of cell and matrix components of ascending thoracic aortic aneurysm

**Berta Ganizada**[1,2], Shaiv Parikh[3], Mitch Ramaekers[4], Armand Jaminon[2], Asim Cengiz Akbulut[2], Ehsan Natour[1], Ryan Accord[5], Joachim Ernst

Wildberger[4], Simon Schalla[1], Jos Maessen[1], Reesink[3], Elham Bidar[1], Leon Schurgers.[2]

[1]Department of Cardiothoracic Surgery, Maastricht University Medical Centre+, Maastricht, The Netherlands, [2]Department of Biochemistry, Cardiovascular Research Institute Maastricht, Maastricht University, Maastricht, The Netherlands, [3]Department of Biomedical Engineering, Maastricht University, Maastricht, The Netherlands, [4]Department of Radiology and Nuclear Medicine Cardiology, Maastricht University Medical Centre+, Maastricht, The Netherlands, [5]Department of Pediatric and Congenital Cardiothoracic Surgery, University Medical Center Groningen, Groningen, The Netherlands.

**Background:** Current indication for ascending thoracic aortic aneurysm (aTAA) surgery is based on aortic diameter of 5–5.5 cm or a growth rate of > 0.5 cm/year [1]. However, current screening surveillance and risk estimation simplifies the complexity of aTAA disease, which might lead to a high-risk open-chest cardiac surgery [2]. Our aim was to examine ex vivo histological features of aTAA specimens, to assess changes in extracellular matrix (ECM) content and vascular smooth muscle cell (VSMC) properties.

**Methods**: Surgical samples of the ventral aspect of the ascending aorta were collected from patients suffering from aTAA (n = 20) and patients with non-aneurysmal coronary bypass or stenotic valve surgery which served as controls (n = 10). Medial cross-sectional thickness, collagen/elastin content, and VSMC number were determined by quantitative histomorphometry. In addition, immunohistochemical markers of VSMC phenotype, α-smooth muscle actin (α-SMA), calponin-1 (CNN1), and S100 calcium binding protein A4 (S100A4) were assessed. Image quantification analysis was performed using QuPath.

**Results**: Aneurysmal aortas showed increased elastin fragmentation and regionally more dense collagen I/III confirming medial degeneration. This resulted in a marked rise in collagen-to-elastin ratio (Table 1). Medial cross-sectional thickness and number of VSMCs were increased in aneurysmal aortas (Table 1). Expression of α-SMA and CNN1 decreased significantly, whilst S100A4 expression was not different between the groups (Table 1).

**Conclusions**: Our preliminary results support the notion of an imbalanced interaction between ECM-VSMCs that may play a crucial role in arterial remodeling cascade, leading to aTAA formation. Further research is needed with the ultimate aim to guide clinical management.

**Table 1. Clinical characteristics and morphometric differences between aneurysmal and control aortas**

| | Aneurysmal | Control | *p*-value |
|---|---|---|---|
| **Subject characteristics** | | | |
| Number of subjects | 20 | 10 | |
| Aortic diameter (mm) | 52 [46.5-59.3] | 38 [37.5-42.0] | |
| Age (years) | 61±14 | 64±12 | |
| Gender (male-%) | 74 | 89 | |
| Weight (kg) | 83±16 | 79±14 | |
| Hypertension-% | 79 | 79 | |
| Diabetes mellitus-% | 9 | 4 | |
| Hypercholesterolemia-% | 62 | 68 | |
| Valve morphology-% | 30 | 21 | |
| Aortic stenosis-% | 19 | 54 | |
| **Quantitative histomorphometry** | | | |
| **Wall thickness** | | | |
| Medial cross-section thickness (mm) | 1.55 [1.23-1.81] | 1.22 [1.13-1.32] | 0.009 |
| **ECM content** | | | |
| Collagen area fraction-% | 37.4 [28.9-44.0] | 35.4 [31.8-39.6] | 0.773 |
| Elastin area fraction-% | 36.0 [27.6-41.5] | 41.5 [40.3-47.7] | 0.022 |
| Collagen-to-elastin ratio | 1.00 [0.9-1.7] | 0.77 [0.7-0.9] | 0.005 |
| **Cell count** | | | |
| VSMC cells per $\mu m^2$ | 1.76E-03 | 1.25E-03 | 0.027 |
| **VSMC phenotype** | | | |
| α-SMA area fraction-% | 12.8 [8.4-18.6] | 19.4 [14.6-33.0] | 0.036 |
| CNN1 area fraction-% | 15.2 [9.9-19.3] | 27.7 [16.5-28.6] | 0.016 |
| S100A4 area fraction-% | 6.4 [4.2-19.6] | 6.9 [2.1-9.3] | 0.415 |

Age and weight are represented as mean ± SD and other values as median with IQR [Q1-Q3].

**References**

1. Erbel R, Aboyans V, Boileau C, et al. 2014 ESC Guidelines on the diagnosis and treatment of aortic diseases: Document covering acute and chronic aortic diseases of the thoracic and abdominal aorta of the adult. The Task Force for the Diagnosis and Treatment of Aortic Diseases of the European Society of Cardiology (ESC) [published correction appears in Eur Heart J. 2015 Nov 1;36(41):2779]. Eur Heart J. 2014;35(41):2873–2926.
2. Cebull HL, Rayz VL, Goergen CJ. Recent Advances in Biomechanical Characterization of Thoracic Aortic Aneurysms. Front Cardiovasc Med. 2020;7:75

**Keywords**: Ascending aorta, Aneurysms, ECM-VSMC, Arterial remodeling

### O.12

### C–C chemokine ligand 5 from subcutaneous adipose tissue has a central role in vascular aging

__Laura Le Pelletier__[1]

[1]UMRS938 Inserm Saint Antoine Research Center, Paris, France

**Background:** Adipose tissue (AT) has a critical role in cardiovascular diseases – particularly through its secretory activity. Aging is associated with AT redistribution, senescence, and changes in the secretome (1,2). We have previously shown that human adipose stromal cells (ASCs) from the subcutaneous AT (SCAT) of aged women display senescence and oxidative stress (3). We hypothesized that the ASC secretome contributes to the onset of endothelial dysfunction, an early stage in vascular aging.

**Methods**: Conditioned media were prepared from ASCs isolated from SCAT of healthy young (< 25y) or aged (> 60y) women. ASCs' secretome were added to human coronary artery endothelial cells. C–C-chemokine-ligand-5 (CCL5) was identified by an adipokine array. The expression of CCL5 in SCAT from men with coronary disease was evaluated. The effect of a CCL5 receptor antagonist, maraviroc, was investigate in peripheral blood mononuclear cells (PBMCs) in HIV-infected individuals from two studies.

**Results**: The secretome of aged-donor ASCs induced endothelial cell dysfunction and senescence. We showed that CCL5 was responsible for these effects and corroborate in experiments with recombinant protein and maraviroc. We observed that CCL5 expression in SCAT of patients with coronary heart disease was strongly associated with blood pressure. Moreover, maraviroc prevented endothelial cell dysfunction in vitro and reverted PBMC senescence in HIV-infected individuals.

**Conclusions**: Our results highlighted the ability of the CCL5 secreted by aged ASCs from SCAT to induce endothelial dysfunction and senescence—both of which are early steps in vascular aging—and a potential link between these phenomena and hypertension.

**References**

1.Stout MB, Justice JN, Nicklas BJ, Kirkland JL. Physiological Aging: Links Among Adipose Tissue Dysfunction, Diabetes, and Frailty. Physiol Bethesda Md. janv 2017;32(1):9-19.
2.Liu Z, Wu KKL, Jiang X, Xu A, Cheng KKY. The role of adipose tissue senescence in obesity- and ageing-related metabolic disorders. Clin Sci Lond Engl 1979. 31 janv 2020;134(2):315-30.
3.Le Pelletier L, Mantecon M, Gorwood J, Auclair M, Foresti R, Motterlini R, et al. Metformin alleviates stress-induced cellular senescence of aging human adipose stromal cells and the ensuing adipocyte dysfunction. eLife. 21 sept 2021;10:e62635.

**Keywords**: Aging, adipose tissue, endothelial dysfunction, adipose stromal cells

### O.13

### Cardiovascular risk in adolescents translates into lower carotid intima-media thickness and better distensibility in young adults—The KiGGS2-cohort

**Karsten Königstein**[1,2,3], Julia Büschges[2,3], Arno Schmidt-Trucksäss[1], Hannelore Neuhauser[2,3]

[1]University of Basel, Basel, Switzerland, [2]Robert-Koch Institute, Berlin, Germany, [3]DZHK (German Centre for Cardiovascular Research), Berlin, Germany

**Background:** Lifestyle-associated cardiovascular risk may be elevated already during adolescence translating into an increased disease burden in adulthood. The KiGGS cohort characterizes cardiovascular aging from childhood until young adulthood in the German general population. This study analyzes the effects of increased cardiovascular risk during adolescence on carotid properties in young adults.

**Methods**: 1,545 participants of the representative healthy population sample of the national KiGGS-0 cohort (10–17 years of age) had carotid ultrasound-assessment 10 years later at the KiGGS-2 follow-up (20–28 years of age). A cardiovascular risk score (CV-R) was calculated at KiGGS-0 including variables of arterial hypertension, obesity, dyslipidemia and smoking. Carotid intima-media thickness (CIMT) and distensibility (DC) at KiGGS-2 were associated with CV-R.

**Results**: Unfavorable alterations of all components of CV-R were associated with higher CIMT and/or reduced DC. Relative risks for pathologically elevated CIMT $\geq$ 90th percentile and/or decreased DC $\leq$ 10th percentile were elevated in participants with 'intermediate' (RRCIMT = 1.89 [1.23–2.91], $p < 0.05$; RRDC = 1.27 [0.79–2.06]) or 'high' risk (RRCIMT = 1.83 [0.95–3.52], $p < 0.1$; RRDC = 1.76 [0.93–3.32], $p < 0.1$) according to CV-R.

**Conclusions**: If an intermediate or high cardiovascular risk according to CV-R is apparent in adolescence, signs of early vascular aging may occur at a very young age. The promotion of a favorable lifestyle to reduce risk factor burden even in the overall healthy general population at a young age seems to be crucial for primary prevention of cardiovascular diseases.

**Keywords**: carotid intima-media thickness (cIMT), primary prevention, cardiovascular risk, adolescents

### O.14

### Evaluation of skin microvascular dysfunction with Laser Speckle Contrast Analysis in prediabetes

Stamatina Lamprou[1], **Nikolaos Koletsos**[1], Ioanna Zografou[2], Gesthimani Mintziori[3], Konstantinos Mastrogiannis[1], Michael Doumas[2], Eugenia Gkaliagkousi[1], Areti Triantafyllou.[1]

[1]Third Department of Internal Medicine, Papageorgiou General Hospital, Aristotle University of Thessaloniki, Thessaloniki, Greece, [2]Second Propedeutic Department of Internal Medicine, Hippokration General Hospital, Aristotle University of Thessaloniki, Thessaloniki, Greece, [3]Unit of Reproductive Endocrinology, 1st Department of Obstetrics and Gynecology, Papageorgiou General Hospital, Aristotle University of Thessaloniki, Thessaloniki, Greece

**Objectives:** Prediabetes is recognized as a significant metabolic status, being a key factor in the occurrence of diabetes mellitus (DM). There is increasing evidence concerning microvascular complications in prediabetes most commonly in skin, kidneys and retina (1,2). Laser speckle contrast analysis (LASCA) is a non-invasive technique that can be used to evaluate skin microvascular function. Previous studies have shown skin microvascular dysfunction in patients with DM (3). However, to our knowledge, no previous study has evaluated skin microcirculation, using LASCA, in patients with prediabetes.

**Methods**: In all subjects, forearm skin blood flow was recorded under standardized conditions using a laser speckle contrast imager (PeriCam PSI NR System, Perimed). Post-occlusive reactive hyperemia (PORH) was assessed following a standardized protocol and data were analyzed with a signal processing software (PIMSoft, Perimed). The amplitude of PORH responses was expressed as a percentage increase between peak and baseline perfusion (%).

**Results**: Twenty-nine individuals (14 patients with prediabetes and 15 controls) were studied. There wasn't any statistically significant difference regarding age, sex, body mass index and blood pressure levels between the two groups. At baseline, skin microvascular perfusion was significantly higher in patients with prediabetes compared to controls (50.9 ± 11.5 vs. 39.2 ± 8.7, $p = 0.006$) while during occlusion, perfusion was similarly reduced in both groups. Post occlusion reperfusion was significantly lower in the prediabetes group as compared to the controls (145.0 ± 42.8 vs. 195.2 ± 47.3% respectively, $p = 0.007$).

**Conclusions**: We showed, for the first time, that individuals with prediabetes demonstrated skin microvascular dysfunction, that may reflect a more generalized microvascular damage.

#### References

1. Lamparter J, Raum P, Pfeiffer N, Peto T, Höhn R, et al. Prevalence and associations of diabetic retinopathy in a large cohort of prediabetic subjects: The Gutenberg Health Study. J Diabetes Complications. 2014;28(4):482–7.
2. Friedman A, Marrero D, Ma Y, Ackermann R, Narayan KMV, et al. Value of urinary albumin-to-creatinine ratio as a predictor of type 2 diabetes in pre-diabetic individuals. Diabetes Care. 2008;31(12):2344–8.
3. de Matheus AS, Clemente ELS, de Lourdes RM, Torres VDC, Gomes MB, et al. Assessment of microvascular endothelial function in type 1 diabetes using laser speckle contrast imaging. J Diabetes Complications. 2017, 31(4):753–7.

**Keywords**: prediabetes, microcirculation dysfunction, LASCA

### O.15

### Arterial Stiffness Assessment by Pulse Arrival Time: An In Silico Proof of Concept.

**Jingyuan Hong**[1], Manasi Nandi[2], Jordi Alastruey.[1]

[1]School of Biomedical Engineering & Imaging Sciences, King's College London, London, UK, [2]School of Cancer & Pharmaceutical Sciences, King's College London, London, UK

**Arterial** stiffness (AS) is one of the primary symptoms of vascular ageing (1). Stiffer arteries lead to increased pulse wave velocity (PWV) and decreased pulse transit time (PTT). PWV is considered the clinical gold standard for the diagnosis of AS, but direct measurement in daily life is challenging (2). Pulse arrival time (PAT), which consists of the pre-ejection period (PEP) and PTT, is defined as the time interval between the R-peak of electrocardiogram (ECG) and a characteristic point of photoplethysmogram (PPG) (3,4). Since most standard wearable devices can capture PPG and ECG signals, and PAT correlates highly with vascular properties, such as vascular tone, PAT extracted from wearable signals has the potential to indicate cardiovascular health (5). The study used a database of in silico pulse wave signals for 4,374 virtual subjects to calculate aortic PWV (aPWV), aortic-radial PTT (arPTT), and PEP (6). The strength of the correlation between PAT and aPWV was assessed using the correlation coefficient (R2). Relative sensitivity analysis was used to investigate the effects of cardiac and vascular properties on PAT. The R2 value between PAT and aPWV was 0.84. The inverse relationship between PAT and aPWV illustrates that stiffer arteries resulted in decreased PAT, even when considering specific age groups. According to the relative sensitivity analysis, PAT is mainly affected by stroke volume and PWV. Our in silico study suggests that PAT has the potential to be used as a marker for assessing the arterial stiffening component of vascular ageing.

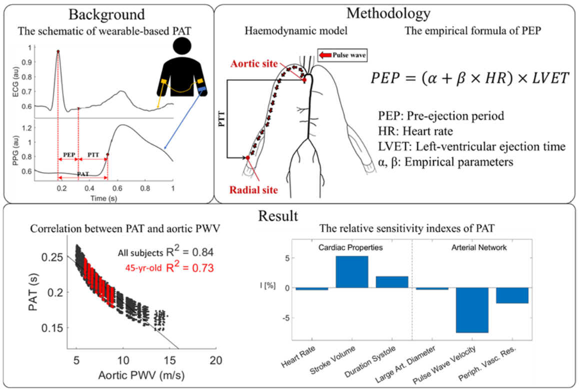

Schematic representation of the definition of pulse arrival time (Background), the extraction method of pulse transit time and pre-ejection period (Methodology), and arterial stiffness analysis of pulse arrival time (Result).

**References**

1. Olsen MH, Angell SY, Asma S, Boutouyrie P, et al. The Lancet. 2016 Nov;388(10060):2665–712.
2. Willum Hansen T, Staessen JA, Torp-Pedersen C, Rasmussen S, et al. Circulation. 2006 Feb;113(5):664–70.
3. Zhang G, Gao M, Xu D, Olivier NB, Mukkamala R. Journal of Applied Physiology. 2011 Dec;111(6):1681–6.
4. Zheng YL, Ding XR, Poon CCY, Lo BPL, et al. IEEE Trans Biomed Eng. 2014 May;61(5):1538–54.
5. Zheng YL, Yan BP, Zhang YT, Poon CCY. Ann Biomed Eng. 2015 Sep;43(9):2242–52.
6. Charlton PH, Mariscal Harana J, Vennin S, Li Y, Chowienczyk P, Alastruey J. Am. J. Physiol.-Heart Circ. Physiol. 2019 Nov;H1062–85.

**Keywords**: Vascular ageing, Arterial stiffness, Pulse arrival time, Pulse wave velocity, Wearable signals

### O.16

### Prevalence and determinants of vascular aging: the LEAD study.

**Kathrin Danninger**[1], Otto Burghuber[2], Marie-Kathrin Breyer[2], Patricia Puchhammer[2], Alina Ofenheimer[2], Robab Breyer-Kohansal[2], Christoph Kaufmann[3], Sylvia Hartl[2], Thomas Weber[1]

[1]Klinikum Wels-Grieskirchen, Department of Cardiology, Linz, Austria, [2]Ludwig-Boltzmann-Institute for Lung Diseases, Vienna, Austria, [3]Klinikum Ottakring, Department of Cardiology, Vienna, Vienna

**Background:** Vascular aging (VA) is an important and prognostically relevant aspect of biological aging. Its determinants are incompletely understood, and a holistic view is missing.

**Methods**: The LEAD (Lung, Heart, Social, Body) study is an ongoing, longitudinal, population-based observational study, which started in 2011 in Vienna and six villages from Lower Austria. As part of the study, cfPWV was measured non-invasively using applanation tonometry (SphygmoCor device, Atcor medical). In a predefined healthy normal population (non-smokers without known hypertension, diabetes, hyperlipidemia, or cardiovascular disease, free from antihypertensive and lipid-lowering medication, blood pressure of < 130/85 mmHg), age-specific Z-scores for cfPWV were calculated. Healthy VA (HVA), normal (NVA) and early (EVA) VA was defined as cfPWV value < 10th, 10th-90th, and > 90th percentile, respectively.

**Results**: In the overall population (n = 7924, 54.2% women, age 18–82 years), the prevalence of HVA/NVA/EVA was 7.8/68.1/24.1%, respectively, with EVA prevalence increasing in older age. NVA and EVA, as compared to HVA, were independently associated with anthropometric (BMI), metabolic (HbA1c), psychosocial (family status) and lifestyle (pack years, alcohol intake) factors, on top of age, gender, and blood pressure (Figure). Additional associations with VA categories were found in younger and older age (level of education), in middle age (income), and in older age (lack of physical activity).

**Conclusions**: In this large population-based study we found a high percentage of early vascular aging, with a significant increase with increasing age. Psychosocial and lifestyle factors seem to play an independent role.

| *Parameter* | *coefficient* | *p-value* |
|---|---|---|
| Age years | 0.0035 | <0.0001 |
| Body Mass Index kg/m² | -0.0061 | 0.001 |
| Gender (m-0, f-1) | -0.04 | 0.007 |
| SBP mm Hg | 0.007 | <0.0001 |
| HbA1c % | 0.066 | <0.0001 |
| Alcohol regular y/n | 0.057 | 0.015 |
| Smoking pack years | 0.01 | 0.04 |
| Metabolic syndrome y/n | 0.074 | 0.0006 |
| Family status* | -0.014 | 0.049 |

1...single, 2...married living together, 3...married separated, 4...divorced, 5...widowed, 6...partnership living together, 7...partnership separated

**Keywords**: Vascular aging

### O.17

### Hydrochlorothiazide, but not chlortalidone nor furosemide, enhances vascular calcification in CKD rats with mineral bone disorder

**Mohsen Agharazii**[1], Richard Larivière[1], Roth-Visal Ung[1], Sylvain Picard[1], Dailson N. de Souza[1], Darren E. Richard[1], Fabrice Mac-Way[1]

[1]Chu De Québec-université Laval, Québec, Canada

**Background:** Previously, we reported that hydrochlorothiazide (HCTZ)-based regimen aggravated arterial calcification and stiffness in a rat model of chronic kidney disease (CKD) with mineral and bone disorder (MBD). In this study, we investigated if all diuretic-based treatments aggravate of arterial stiffness and calcification in CKD-MBD rats.

**Methods**: In rats with renal mass ablation-induced CKD, MBD was generated by a Ca/P-rich diet and calcitriol. The animals were divided into four groups; (1) CKD-MBD control; (2) CKD-MBD + HCTZ (thiazide diuretic, 5 mg/kg/d); (3) CKD-MBD + Chlorthalidone (thiazide-like diuretic, 5 mg/kg/d), and; (4) CKD-MBD + Furosemide (loop diuretics, 10 mg/kg/d). At week 6, systolic and mean blood pressure (SBP and MBP), pulse pressure (PP) and pulse wave velocity (PWV) were determined invaisively. Thoracic aorta calcification was assessed by von Kossa staining.

**Results**: SBP was reduced by all types of diuretics. Contrary to chlortalidone and furosemide, HCTZ treatment led to a reduction of MBP, but an increase in PP and PWV in CKD-MBD rats (p < 0.05). As expected from these hemodynamic changes, medial calcification in the thoracic aorta was significantly greater in CKD-MBD rats treated with HCTZ as compared to all the other groups of rats (p < 0.05).

**Conclusions**: In rats with CKD-MBD, HCTZ, but not other types of diuretic, exacerbated arterial stiffness and vascular calcification despite a reduction in SBP. The deleterious effect of HCTZ in CKD-MBD rats may have major clinical impact as this diuretic is widely use in patients with CKD that often develop MBD.

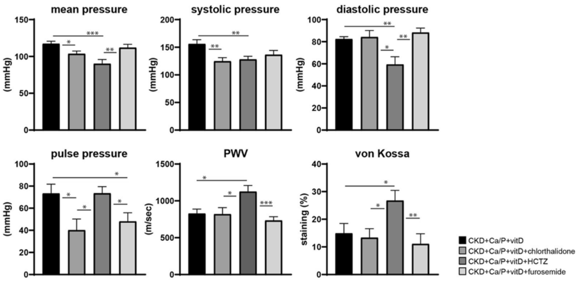

The figures shows blood pressure (systolic, mean, diastolic, pulse pressure), and aortic stiffness by pulse wave velocity (PWV), and aortic calcification by von Kossa staining.

**Keywords**: Chronic kidney disease, mineral bone disease, vascular calcification

### O.18

### Relationships between excessive daytime sleepiness, arterial stiffness, and physical activity. The Atherosclerosis Risk in Communities (ARIC) Study

Michael A Newton, Rachael Hamm, Emma Barinas-Mitchell, Pamela L Lutsey, Hirofumi Tanaka, Kapuaola Gellert, Lee Stoner, **Michelle Meyer**[1]

[1]University of North Carolina at Chapel Hill, Chapel Hill, United States, [2]University of North Carolina at Chapel Hill, Chapel Hill, United States, [3]University of Pittsburgh, Pittsburgh, United States, [4]University of Minnesota Twin Cities, Minneapolis, United States, [5]University of Texas at Austin, Austin, United States, [6]University of Hawaii, Honolulu, United States, [7]University of North Carolina at Chapel Hill, Chapel Hill, United States, [8]University of North Carolina at Chapel Hill, Chapel Hill, United States

**Background:** Excessive Daytime Sleepiness (EDS) is associated with higher risk of cardiovascular disease (CVD) events (1,2) and mortality (3). However, the association between EDS and subclinical CVD, such as arterial stiffness, is not fully understood nor is the role of physical activity (PA) in this association. We examined the relationship between EDS and arterial stiffness, measured using carotid-femoral pulse wave velocity (cfPWV), with PA as a potential effect modifier.

**Methods**: A cross-sectional analysis of ARIC Study participants (n = 2349, mean age: 79.6, 57.2% female, 19.2% black adults) who underwent cfPWV measures (VP-1000 Plus, Omron Co., Kyoto, Japan) and completed the Epworth Sleep Scale (ESS) and Baecke questionnaires in 2016–2019. EDS was defined as ESS ≥ 11. We calculated moderate-vigorous PA (min/week) and categorized PA based on the distribution and guidelines. We used multivariable linear regression to estimate the association between ESS, EDS, and cfPWV, and evaluated effect modification by PA. Results are presented as beta coefficients (β) and 95% confidence intervals (CI).

**Results**: A total of 14.4% participants reported EDS. The association of ESS and EDS with cfPWV differed by PA level. The association of ESS (Figure A) and EDS (Figure B) with cfPWV became more negative with higher PA levels, although the associations with EDS were not statistically significant.

**Conclusion**: A negative association was observed between ESS and cfPWV at the most intense level of PA in older adults. In those not meeting PA guidelines, other adverse life and participant characteristics could outweigh the effects of ESS and EDS on cfPWV.

Figure. Association of Epworth Sleepiness Scale (ESS) and excessive daytime sleepiness (EDS) with carotid-femoral pulse wave velocity (cfPWV) in ARIC Visit 6/7 (N= 2,349) by physical activity (PA)

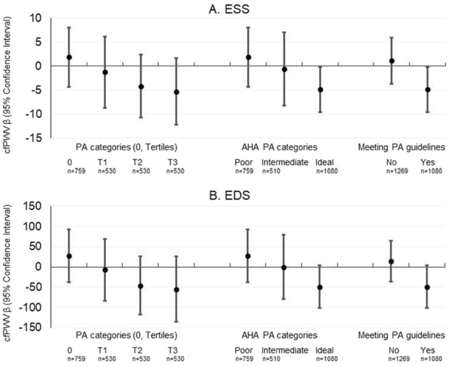

Results are adjusted for age, race-study center, body mass index, diabetes, heart rate, mean arterial pressure, and blood-pressure medication use. AHA: American Heart Association: T: tertile.

**References**

1. Blachier M, Dauvilliers Y, Jaussent I et al. Excessive daytime sleepiness and vascular events: the Three City Study. Ann Neurol. 2012;71(5):661–7.
2. Lee CH, Ng WY, Hau W et al. Excessive daytime sleepiness is associated with longer culprit lesion and adverse outcomes in patients with coronary artery disease. J Clin Sleep Med. 2013;9(12):1267–72.
3. Li J, Covassin N, Bock JM et al. Excessive Daytime Sleepiness and Cardiovascular Mortality in US Adults: A NHANES 2005–2008 Follow-Up Study. Nat Sci Sleep. 2021;13:1049–59.

**Keywords**: Arterial stiffness, sleep, physical activity, older adults

### O.19

### Greater intrinsic arterial wall stiffness and its unfavourable trajectory over time in type 2 diabetes

**Kunihiko Aizawa**[1], Phillip E Gates[1], David M Mawson[1], Francesco Casanova[1], Kim M Gooding[1], Suzy V Hope[1], Isabel Goncalves[2,3], Jan Nilsson[2], Faisel Kahn[4], Helen M Colhoun[5], Andrea Natali[6], Carlo Palombo[7], Angela C Shore[1]

[1]NIHR Exeter Clinical Research Facility, University of Exeter Medical School, Exeter, United Kingdom, [2]Department of Clinical Sciences, Lund University, Malmö, Sweden, [3]Department of Cardiology, Skåne University Hospital, Malmö, Sweden, [4]Division of Systems Medicine, University of Dundee, Dundee, UK, [5]Centre for Genomic and Experimental Medicine, University of Edinburgh, Edinburgh, UK, [6]Department of Clinical and Experimental Medicine, University of Pisa, Pisa, Italy, [7]Department of Surgical, Medical, Molecular and Critical Area Pathology, University of Pisa, Pisa, Italy

**Background:** A greater central artery stiffness is observed in people with type 2 diabetes (T2DM). However, it is unclear about intrinsic arterial wall stiffness in these patients. We aimed to determine the utility of $\beta_o$ cross-sectionally and longitudinally in T2DM. METHODS: We studied 753 adults with T2DM (DM+: 67.5 ± 8.3 years, 227F) and 436 adults without T2DM (DM−: 67.0 ± 9.2 years, 159F) cross-sectionally (Phase 1), and subsequently studied 310 adults in DM + (68.6 ± 7.6 years, 104F) and 210 adults in DM− (67.6 ± 8.5 years, 83F) over three years longitudinally (Phase 2). Carotid-femoral pulse wave velocity was measured, and its data were used to calculate $\beta_o$ as previously described[1].

**Results**: In Phase 1, $\beta_o$ was significantly greater in DM+than DM− after adjusting for age and sex [27.5 (26.6–28.3) vs 23.6 (22.4–24.8) au, p<0.001]. Partial correlation analyses after adjusting for age and sex found that $\beta_o$ was significantly associated with HbA1c (r=0.15 p<0.001) and heart rate (r=0.23 p<0.001) in DM+. I Phase 2, percentage changes in $\beta_o$ were significantly greater in DM+than DM− [19.5 (14.9–24.0) vs 5.0 (−0.6–10.6) %, p<0.001] after adjusting for age, sex and baseline $\beta_o$. Multivariable linear regression analyses revealed that the percentage changes in $\beta_o$ were independently associated with percentage changes in heart rate in DM+ (overall $R^2=0.19$).
**Conclusion**: $\beta_o$ was greater in DM+than DM−. Furthermore, $\beta_o$ changed over three years with ageing but it changed much more in DM+than DM−. These data suggest that intrinsic arterial wall stiffness may be a useful target for therapeutic intervention.

**Reference**
[1]Spronck et al. J Hypertens 2017;35:98–104.

**Keywords**: Ageing, Aorta, Blood pressure, Heart rate

### O.20
### The bidirectional longitudinal relationships between arterial stiffness and hypertension and those between arterial stiffness and diabetes mellitus

**Professor Hirofumi Tomiyama**[1]

[1]Japan, Suginami, Japabn

**Background:** Hypertension and diabetes mellitus frequently coexist; however, it has not yet been clarified if the bidirectional longitudinal relationships between arterial stiffness and hypertension are independent of those between arterial stiffness and diabetes mellitus.
**Methods**: In this 16-year prospective observational study, 3960 middle-aged employees of a Japanese construction company without hypertension/diabetes mellitus at the study baseline underwent annual repeated measurements of the blood pressure, serum glycosylated hemoglobin A1c level (HbA1c), and brachial-ankle pulse wave velocity (baPWV).
**Results**: By the end of the study period, 664, 779, 154, and 406 subjects developed hypertension, prehypertension, diabetes mellitus, and prediabetes, respectively. Increased baPWV at the baseline was associated with a significant odds ratio (per 1 standard deviation increase) for new onset of prehypertension/hypertension with (2.45/3.28, P<0.01) or without (2.49/2.76, P<0.01) coexisting prediabetes/diabetes mellitus, but not for new onset of prediabetes/diabetes mellitus without coexisting hypertension. Analyses using the latent growth curve model confirmed the bi-directional relationships between baPWV and hypertension, but no such relationship was observed between baPWV and abnormal glucose metabolism.
**Conclusions**: In middle-aged Japanese subjects in contrast to the bi-directional relationships that exist between arterial stiffness and hypertension, increased arterial stiffness preceding the development of diabetes mellitus may represent that associated with the development of hypertension, as it is observed only in cases of diabetes mellitus coexisting with hypertension. Therefore, arterial stiffness may be associated to a greater degree with the development of hypertension than with the development of diabetes mellitus.
**Keywords**: arterial stiffness; hypertension

### O.21
### Characterization of the sex-specific pattern of angiogenesis and lymphangiogenesis in aortic stenosis

**Lara Matilla Cuenca**[1], Ernesto Martín-Núñez[1], Mattie Garaikoetxea Zubillaga[1], Adela Navarro[1], Julieta Anabela Vico[1], Vanessa Arrieta[1], Amaia Garcia-Peña[1], Amaya Fernández-Celis[1], Alicia Gainza[1], Virginia Álvarez[1], Rafael Sádaba[1], Natalia López-Andrés[1], Eva Jover García[1]

[1]Cardiovascular Translational Research, Navarrabiomed, Hospital Universitario de Navarra (HUN), Universidad Pública de Navarra (UPNA), IdiSNA, Pamplona, Spain, Pamplona, Spain

**Background:** The pathophysiological role of angiogenesis and lymphangiogenesis in aortic stenosis (AS) remains unknown. Valve avascularity is seemly abrogated in AS and neovascularization is well-correlated with the disease(1,2). We study sex-related differences in angiogenesis and lymphangiogenesis in aortic valves (AVs) and valve interstitial cells (VICs) from AS patients.
**Methods**: 226 patients recruited (60.6% men) with severe AS undergoing surgical valve replacement.
**Results**: The density of total neovessels was higher in AVs from men versus women's. Small and medium neovessels were more abundant in men's AVs. Male AVs exhibited enhanced CD31 and VE-cadherin expressions. Levels of the pro-angiogenic markers [vascular endothelial growth factor (VEGF)-A, VEGF receptor (VEGFR)1, VEGFR2, insulin-like growth factor-binding protein-2 (IGFBP-2), interleukin (IL)-8, chemerin and fibroblast growth factor (FGF)-7] were increased in men's AVs. Transforming growth factor-β expression was higher in male AVs. Expression of antiangiogenic molecules [thrombospondin (Tsp)-1, endostatin and CD36] was upregulated in male AVs, although the levels of Tsp-2, IL-4, IL-12p70 and chondromodulin-1 were similar between sexes. The number of lymphatic vessels and the expression of the lymphangiogenic markers Lyve-1 and D2-40 was enhanced in men's AV also VEGF-C, VEGF-D and VEGFR3. VICs isolated from men's AVs secreted higher amounts of pro-angiogenic (VEGF-A, VEGFR1, IGFBP-2 and FGF-7) and pro-lymphangiogenic factors (VEGF-C, VEGF-D and VEGFR3) than women's without changes in antiangiogenic markers.
**Conclusions**: We show that aberrant angiogenic and lymphangiogenic cues are over-represented in male AVs. VICs are a relevant source of multiple morphogens involved in angiogenesis and lymphangiogenesis likely endowing the AV of men with the predominant calcific AS phenotypes (3,4).

**References**
1. Collett GD, Canfield AE. Angiogenesis and pericytes in the initiation of ectopic calcification. Circ Res. 2005;96(9):930–8.
2.Rajamannan NM, et al. Calcified rheumatic valve neoangiogenesis is associated with vascular endothelial growth factor expression and osteoblast-like bone formation. Circulation. 2005;111(24):3296–301.
3.Gerber HP, Vu TH, Ryan AM, Kowalski J, Werb Z, Ferrara N. VEGF couples hypertrophic cartilage remodeling, ossification and angiogenesis during endochondral bone formation. Nat Med. 1999;5(6):623–8.
4.Nelson V, Patil V, Simon LR, Schmidt K, McCoy CM, Masters KS. Angiogenic Secretion Profile of Valvular Interstitial Cells Varies With Cellular Sex and Phenotype. Front Cardiovasc Med. 2021;8:736303.

**Keywords**: Aortic-stenosis, sex, angiogenesis, lymphangiogenesis

### O.22
### Arterial stiffness predicts sustained hypertension in patients with high normal blood pressure/grade 1 hypertension

**Dr. Enrique Rodilla**[1,2], Dr. Andrea Mendizábal[1], Dr. Iratxe Jiménez[1], Dr. Leticia Pérez[1], Dr. Sergio Canales[1], Dr. Alicia Roldán[1], Dr. Santiago Pintos[1], Dr. Marta Catalán[1], Dr. María-Carmen Sáez[1], Dr. José Chordá[2], Dr. José-Antonio Costa[2]

[1]Hospital Universitario de Sagunto, Puerto De Sagunto, Spain, [2]Universidad Cardenal Herrera-CEU, CEU Universities, Moncada, Spain

**Background:** 2018 ESC-ESH Guidelines for the Management of Arterial Hypertension recommend pharmacological treatment if patients with grade 1 HTN at low-moderate risk remain hypertensive after a period of lifestyle intervention. Our objective was to assess the predictive value of early vascular aging (EVA) to identifying patients who developed sustained HTN after baseline diagnosis.

**Methods**: Retrospective, descriptive, longitudinal study including all consecutive patients referred to a HTN Unit with suspected naïve HTN without prior pharmacological treatment. EVA was defined according to estimated pulse wave velocity (brachial oscillometry, Mobil-O-Graph (IEM®) in seven age-groups(1). Standard clinical tests were performed at baseline and after 12 months.

**Results**: Since 2010, 335 consecutive patients entered the study, with 201 women (60%), a mean age of 46,4 years (±13), mean office BP of 130/76 (±12/9), and ambulatory BP of 122/78 (±8/7) mmHg. Distribution of BP was 155 (46.3%) patients with high-normal BP, 28 (8,4%) with white-coat HTN, 108 (32.2%) with masked HTN and 44 (13.1%) with established HTN. At baseline, 57% of patients showed EVA, after a mean time of 1.1 year, 65% of participants presented elevated ABPM. In univariate analysis, baseline stiffness (EVA) was associated with elevated ABPM-values in the follow-up visit (OR: 2.0; IC 1.3–3.1; $p = 0.003$). After adjustment for age, gender and pulse pressure, baseline EVA kept its significant predictive value (OR:2.6; IC 1.6–4.2; $p = 0.001$).

**Conclusions**: Arterial damage characterized as EVA according to estimated PWV by brachial oscillometry doubles the probability of sustained HTN one year after initial assessment in naïve patients with high-normal BP/grade 1 HTN at low-moderate risk.

**Reference**

Nunan D, Fleming S, Hametner B, Wassertheurer S. Performance of pulse wave velocity measured using a brachial cuff in a community setting. Blood Press Monit. 2014;19:315–9.

**Keywords**: Early vascular aging (EVA), risk stratification, pulse wave velocity, high-normal blood pressure

### O.23

### Awareness and perceptions of health care providers and researchers on vascular ageing: Quantitative Survey Results.

**Chloe Park**[1], Andrie Panayiotou[2], Thomas Weber[5], Christopher Mayer[4], Areti Triantafyllou[3], Rachel Climie[6]

[1]MRC Unit for lifelong Health and Ageing at UCL, London, United Kingdom, [2]Cyprus International Institute for Environmental and Public Health, Cyprus University of Technology, Limassol, Cyprus, [3]Medical School, Aristoteleio University Thessaloniki, Thessaloniki, Greece, [4]AIT Austrian Institute of Technology GmbH, Vienna, Austria, [5]Cardiology Department, Klinikum Wels-Grieskirchen, Wels, Austria, [6]Menzies Institute for Medical Research, University of Tasmania, Hobart, Australia

**Introduction:** Vascular Age (VA) can assess cardiovascular disease risk, independently of chronological age, however it is not yet widely applied in routine clinical practice. A quantitative questionnaire was developed to assess current knowledge gaps related to VA and barriers to implementation in routine practice in both research and clinical settings.

**Methods**: Using a stepwise mixed-methods approach, a quantitative questionnaire was constructed. The 22-item anonymous survey was based on a previous qualitative analysis including 80 participants with multiple scientific backgrounds, and included questions on perceptions/beliefs, knowledge, and implementation of VA. The survey was disseminated to clinicians and researchers world-wide, via social media and targeted emails from well-known societies (including Artery, ESH, ISH, ESC).

**Results**: 276 (50% female) completed the questionnaire, 46% were clinicians, 33% researchers, 10% students. Clinical specialties included cardiology (36%), internal medicine (22%) and General Practice (10%). While 84% of clinicians and researchers rate VA importance as high or very high (Table), only 11% of clinicians measure VA in clinical settings. Limiting factors include cost, lack of guidelines and lack of knowledge.

**Discussion**: These results show that implementation of VA is very low in clinical settings and awareness of VA needs to be improved via planned targeted awareness strategies and educational material.

| Question | Options | Clinician (n=126) | Researcher/ Academic (n=91) |
|---|---|---|---|
| Measures VA in research setting (%) | - | 33 | 58 |
| Measures VA in clinical setting (%) | - | 11 | 7 |
| How do you measure VA? (%) | Ultrasound | 48 | 58 |
| | Pulse Wave Velocity | 67 | 78 |
| | Central Blood Pressure | 35 | 50 |
| | Endothelial Function | 59 | 53 |
| | MRI | 23 | 40 |
| Who benefits the most from VA measures? (%) | No one | 2 | 1 |
| | Everyone | 32 | 55 |
| Importance of VA (1-5) (%) | Low-medium (1-3) | 16 | 16 |
| | High (4) | 36 | 24 |
| | Very high (5) | 48 | 60 |
| Limitations (%) | Lack of knowledge | 29 | 20 |
| | No limitations | 2 | 21 |
| | Cost | 42 | 23 |
| | Lack of guidelines | 40 | 16 |

A selection of Survey Results

**Keywords**: Survey, Knowledge, Awareness, Vascular age

### O.25

### Early vascular aging associated with sympathetic hyperactivity in obese hypertensive individuals with moderate to severe obstructive sleep apnea

Samanta Mattos[1], Michelle Rabello Cunha[1], Marcia Regina Simas Klein[1], **Mario Neves**[1]

[1]State University of Rio de Janeiro, Rio de Janeiro, Brazil

**Background:** Obstructive sleep apnea (OSA) is an independent cardiovascular risk (CVR) factor. The objective was to evaluate sympathetic tone and vascular disease in obese hypertensive with moderate and severe OSA.

**Methods**: Individuals of both sexes, aged 40–70 years and body mass index (BMI) $\geq 30$ and $< 40$ kg/m2, submitted to assessment of heart rate variability (HRV), central parameters by Mobil-O-Graph and carotid ultrasound. Sleep study was performed through a portable home sleep test device (WatchPAT).

**Results**: Patients ($n = 49$) were divided into two groups based on the apnea–hypopnea index (AHI): absent-mild (AM) group (AHI < 15 events/h, $n = 17$) and moderate-severe (MS) group (AHI ≥ 15 events/h, $n = 32$). The mean BMI was similar (35±3 vs 34±2 kg/m2, $p = 0.248$). Systolic blood pressure (120±15 vs 131±14 mmHg, $p = 0.003$), pulse pressure (43±9 vs 49±8 mmHg, $p = 0.011$), CVR (6.8±4.1 vs 14.4±10.7%, $p = 0.003$) and cardiometabolic age (48±6 vs 52±8 years, $p = 0.034$) were higher in the MS group. The same group presented higher low frequency/high frequency (LF/HF) ratio (0.83±0.56 vs 1.91±1.98, $p = 0.017$), pulse wave velocity (PWV) (7.1±0.7 vs 8.0±1.2 m/s, $p = 0.003$), vascular age (50±6 vs 56±8 years, $p = 0.014$) and carotid intima-media thickness (0.58±0.09 vs 0.70±0.12 mm, $p = 0.001$). PWV was significantly correlated with LF/HF ratio ($r = 0.609$, $p < 0.001$) only in the MS group.

**Conclusion**: In this sample of obese hypertensive patients, moderate to severe OSA was associated with sympathetic hyperactivity and evidence of early vascular aging with increased arterial stiffness and subclinical atherosclerosis.

**Reference**

Bironneau V, Tamisier R, Trzepizur W, et al. Sleep apnoea and endothelial dysfunction: An individual patient data meta-analysis. Sleep Med Rev. 2020 Aug;52:101–309.

**Keywords**: Hypertension; Sleep apnea; Obesity; Arterial stiffness

### O.26

### Acute vasopressin neutralization with the aptamer NOX-F37 improves immediately cardiac but not peripheral endothelial dysfunction in rats with chronic heart failure

Yohan Stephan[1], Marina Hamrouche[1], Lise Charrier[1], Lionel Nicol[1], Marie-Laure Ozoux[2], Philip Janiak[2], Jeremy Bellien[1], **Paul Mulder**[1]

[1]Inserm U1096, Rouen, France, [2]Corteria Pharmaceuticals, Paris, France

**Background:** Vasopressin is one of the leading pathophysiological drivers of chronic heart failure (CHF) acting via V1a-, V1b- and V2 receptors. Selective V2 and dual V1a-V2 receptor antagonists ameliorate plasma sodium levels, but fail to reduce mortality in clinical studies. Vasopressin neutralization is an original alterative for receptor blockers but its effect in CHF is unknown. For this purpose, we sought investigated the short-term cardiac and vascular effects of the vasopressin neutralizing aptamer NOX-F37.
**Methods**: Left ventricular (LV) function (hemodynamics by LV catherization) and LV tissue perfusion (MRI) as well as mesenteric artery endothelium function (flow mediated dilation by arteriograph) were determined 2 h after NOX-F37 administration (80 nM/kg; IP) to rats with well-established CHF induced by coronary artery ligation.
**Results**: Two hours after administration, NOX-F37 significantly improved LV systolic function, illustrated by the significant increase in LV end-systolic pressure volume relation (CHF: 20.2 ± 0.0.7; CHF + NOX: 23.3 ± 1.0 mmHg/RVU) and diastolic function, illustrated by the significant decrease in LV end-diastolic pressure volume relation (CHF: 4.03 ± 0.48; CHF + NOX: 2.06 ± 0.21 mmHg/RVU), which were associated with a significant increase in LV tissue perfusion (CHF: 6.12 ± 0.24; CHF + NOX: 10.10 ± 0.26 ml/min/g LV tissue). However, mesenteric artery flow-induced dilatation was not modified and remained impaired (% dilatation at 150 µl/min; CHF: 10 ± 7; CHF + NOX: 9 ± 8).
Conclusions: These results illustrate the immediate protective effects on cardiovascular function of vasopressin neutralization in chronic heart failure confirming the existence of a deleterious vasopressinergic tone in chronic heart failure. Whether these beneficial cardiac effects persist with chronic vasopressin neutralization needs to be confirmed.

**Keywords**: Heart failure; pharmacology; vasopressin

### O.27

### Hypertensive aortic remodelling as induced by adrenergic receptor activation vrsus renin–angiotensin–aldosterone system activation in mice

**Bart Spronck**[1,2,3], Alexander W. Caulk[2], Abhay B. Ramachandra[2], Sae-Il Murtada[2], Jay D. Humphrey[2,4]

[1]Dept. of Biomedical Engineering, Maastricht University, Maastricht, The Netherlands, [2]Dept. of Biomedical Engineering, Yale University, New Haven, United States, [3]Macquarie Medical School, Macquarie University, Sydney, Australia, [4]Vascular Biology and Therapeutics Program, Yale School of Medicine, New Haven, United States

**Background:** Hypertension causes the aorta to remodel and potentially stiffen. We aimed to compare the aortic remodelling response to hypertension as induced by adrenergic receptor activation versus renin–angiotensin–aldosterone system activation.
**Methods:** Adult male C57BL/6 J mice were studied under seven conditions: untreated, and after 7/14/28-day subcutaneous infusion of 3880 ng/kg/min norepinephrine (NE) or 1000 ng/kg/min angiotensin II (AngII). After euthanasia, ascending/descending thoracic (ATA/DTA) and infrarenal abdominal (IAA) aortas were dissected, placed within a computer-controlled biaxial testing device, and subjected to isobaric (90 mmHg) vasoreactivity experiments to, among others, 1 µM phenylephrine + 1 mM L-NAME [1]. Under passive conditions, pressure-diameter tests were performed at 95/100/105% of the < i > in vivo < /i > axial stretch and axial force–length tests at 10/60/100/140 mmHg. Data were fit using a nonlinear constitutive model [1].
**Results:** Figure (bar charts, < i > n < /i > = 4–8 per group) shows passive metrics calculated at < i > in vivo < /i > axial stretch and group-specific systolic blood pressures [2]. AngII caused larger increases in wall thickness than NE. Both NE and AngII led to significant structural arterial stiffening, driven by a combination of wall thickening and stiffening of the wall material. Figure also shows correlation of wall thickness with contractility (scatter plots, < i > n < /i > = 4–7 per group; symbols represent individual aortas). The stronger an individual aorta was able to contract (larger absolute stress change; to the left on < i > x < /i > -axis), the weaker its remodelling response.
**Conclusions:** NE- and AngII-induced hypertension elicit distinct aortic remodelling responses. However, independent of the hypertensive stimulus, aortic contractile capacity emerged as protective against hypertensive arterial remodelling.

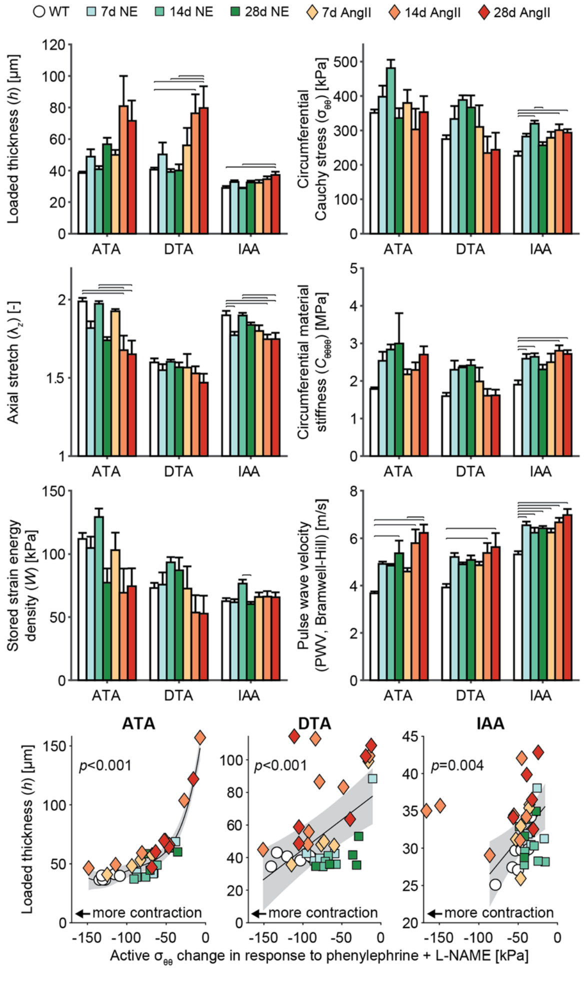

Whiskers: standard errors, overbars: < i > p < /i > < 0.05 (Bonferroni). Systolic/diastolic blood pressures: 120/80 mmHg (WT); 154/103, 162/108, 154/103 mmHg (7/14/28d NE); 150/100, 159/106, 177/118 mmHg (7/14/28d AngII). Lines/grey areas: multilevel regressions/95% confidence intervals.

**References**

[1] Spronck B, Latorre M, Wang M, Mehta S, Caulk AW, Ren P, et al. Excessive adventitial stress drives inflammation-mediated fibrosis in hypertensive aortic remodelling in mice. J R Soc Interface 2021; 18:20,210,336.

[2] Owens AP, 3rd, Subramanian V, Moorleghen JJ, Guo Z, McNamara CA, Cassis LA, Daugherty A. Angiotensin II induces a region-specific hyperplasia of the ascending aorta through regulation of inhibitor of differentiation 3. Circ Res 2010; 106:611–619.

**Keywords**: Angiotensin II; arterial mechanics; norepinephrine; vasoconstriction

### O.28

### Raised arterial stiffness at 24–26 weeks of gestation is associated with the development of hypertension in pregnancy

**Pritika Dutta**[1], Tarang Gupta[1], Rajesh Kumari[1], Vidushi Kulshrestha[1], Ashok Kumar Jaryal[1], Kishore Kumar Deepak[1], Garima Kachhawa.[1]

[1]All India Institute of Medical Sciences, New Delhi, India

**Background:** Increase in arterial stiffness in third trimester is proposed to be involved in the pathogenesis of hypertensive disorders of pregnancy. The present study was conducted to evaluate arterial stiffness in pregnant women at 24–26 + 6 weeks of gestation. They were grouped into healthy pregnancy (HP), preeclampsia (PE) or gestational hypertension (GHTN) depending upon maternal outcome.

**Methods**: Arterial stiffness was measured using applanation tonometry. Central arterial stiffness was quantified by augmentation index normalized to heart rate 75 beats/minutes (AIx@75) and carotid-femoral pulse wave velocity (cfPWV), peripheral arterial stiffness was quantified by carotid-radial pulse wave velocity (crPWV) using SphgmoCor® CVMS CPVH device.

**Results**: Out of 313 women, PE developed in 3.51% (n = 11), GHTN in 5.11% (n = 16) and 22.04% (n = 69) remained healthy pregnant without any obstetrical or medical factors. The mean age in years, BMI in kilograms/metre2 and MAP in mmHg between the groups were [(30.0 ± 1.6 vs 28.81 ± 4.8 vs 27.70 ± 4.0, p = 0.094), {27.41(25.10–33.09) vs 28.51(24.29–31.84) vs 27.01(25.39–28.40), p = 0.529}, (99.09 ± 7.58 vs 95.88 ± 9.09 vs 85.99 ± 9.64, p < 0.0001)] respectively. AIx@75 was found to be increased (PE:22.82 ± 14.65%, GHTN:19.47 ± 10.60%, HP:10.35 ± 12.14%, p = 0.001) in women developing hypertension in pregnancy than normotensive healthy pregnancy. Similarly cfPWV (PE:7.21 ± 1.24 m/s, GHTN:6.64 ± 1.22 m/s, HP: 6.19 ± 1.06 m/s, p = 0.013) was also significantly higher in pregnant women with PE and GHTN. The crPWV were comparable among PE, GHTN and healthy pregnant.

**Conclusion**: Raised central arterial stiffness is observed before the clinical onset of disease in women destined to develop PE later in pregnancy. AIx@75 and cfPWV could be used as a putative prognostic marker of hypertensive disorders of pregnancy.

**References**

1.Garg P, Jaryal AK, Kachhawa G, Kriplani A, Deepak KK. Sequential profile of endothelial functions and arterial stiffness in preeclampsia during the course of pregnancy. Pregnancy Hypertens. 2019; 18: 88–95.

2.Franzi MB, Burgmann M, Neubauer A, Zeisleri H, Sanani R, Gottsauner-Wolf M et al. Augmentation index and pulse wave velocity in normotensive and pre-eclamptic pregnancies. Acta. Obstet. Gynecol. Scand. 2013; 92: 960–66.

3. Tomiyama H, Yamashina A. Non-invasive vascular function tests: Their pathophysiological background and clinical application. Circ. J. 2010; 74: 24–33.

**Keywords**: Arterial stiffness, augmentation index, pulse wave velocity, hypertensive disorders of pregnancy

Posters

### Basic

### P.001

### Variability of invasive aortic pulse wave velocity measured by catheter pull-back method and implications for pulse wave velocity device validation

**Alexander Stäuber**[1], Bart Spronck[2,7], Alessandro Giudici[2,8], Cornelia Piper[3], Siegfried Eckert[3], Stefan Richter[4], Marc-Alexander Ohlow[5], Johannes Baulmann[6]

[1]Professorship of Sports Medicine / Sports Biology, Chemnitz University of Technology, Chemnitz, Germany, [2]Department of Biomedical Engineering, CARIM School for Cardiovascular Diseases, Maastricht University, Maastricht, The Netherlands, [3]Klinik für Allgemeine und Interventionelle Kardiologie/Angiologie, Herz- und Diabeteszentrum NRW, Universitätsklink der Ruhr-Universität Bochum, Bad Oeynhausen, Germany, [4]Department of Cardiology, Zentralklinik Bad Berka GmbH, Herzzentrum, Department of Cardiology, Bad Berka, Germany, [5]Department of Cardiology, SRH Wald-Klinikum, Gera, Germany, [6]Praxis Dres. Gille/Baulmann, Rheinbach, Germany, [7]Macquarie Medical School, Faculty of Medicine, Health and Human Sciences, Macquarie University, Australia, [8]GROW School for Oncology and Reproduction, Maastricht, The Netherlands

**Background:** The invasive method of catheter pullback from ascending aorta (asc) to iliac bifurcation (bifu) is gold standard for aortic pulse wave velocity (aPWV) and the reference standard for validation of non-invasive devices that estimate aPWV [1]. In this work, we examine aPWV variability in invasive recordings of patients undergoing cardiac catheterization.

**Methods:** Invasive measurements were performed in 56 patients (57% male, 67 ± 13 years, mean ± standard deviation (SD)) with a femoral catheter access. Catheter pullback method was used to measure aortic pulse transit time (aPTT) from asc to bifu. Pulse wave analysis using the intersecting tangent method (Sirius, Redwave Medical GmbH, Jena, Germany) provided the diastolic foot points for each recording site (asc, bifu) and recorded heartbeat (number of beats, asc: 86 ± 42, bifu: 82 ± 43). From the respective time difference with the R-wave of the time-synchronised electrocardiogram, the pulse transit time for the corresponding recording site (PTTasc, PTTbifu) was derived for each heartbeat. aPTT was then determined from the difference of the averaged PTTbifu and PTTasc. Based on aPTT, the known catheter pullback length and the estimated SD of aPTT, $SD\_aPTT = \sqrt{(SD\_PTTasc^2 + SD\_PTTbifu^2)}$, the SD of the corresponding aPWV was calculated as $SD\_aPWV = aPWV \times SD\_aPTT/aPTT$ for each patient.

**Results:** aPTT was 44.01 ± 12.89 ms; aPWV was 9.7 ± 3.1 m/s. SD_aPTT was 3.72 ± 1.73 ms, resulting in an SD_aPWV of 1.0 ± 0.8 m/s.

**Conclusions:** Our data indicate a substantial beat-to-beat SD in invasively determined aPWV by catheter pull-back method. The issue of aPWV variability in the invasive reference needs to be addressed in validation protocols for non-invasive estimation of aPWV.

**Reference**

[1] Wilkinson IB, McEniery CM, Schillaci G, Boutouyrie P, Segers P, Donald A, Chowienczyk PJ (2010). ARTERY Society guidelines for validation of non-invasive haemodynamic measurement devices: Part 1, arterial pulse wave velocity. Artery Research, 4(2), 34–40.

**Keywords**: PWV, PTT, variability

### P.002

### Multiple linear regression analysis of age, gender, anthropometric and haemodynamic factors to predict variability in aortic pulse transit time determined by the catheter pull-back method

**Alexander Stäuber**[1], Bart Spronck[2,7], Alessandro Giudici[2,8], Cornelia Piper[3], Siegfried Eckert[3], Stefan Richter[4], Marc-Alexander Ohlow[5], Johannes Baulmann.[6]

[1]Professorship of Sports Medicine/Sports Biology, Chemnitz University of Technology, Chemnitz, Germany, [2]Department of Biomedical Engineering, CARIM School for Cardiovascular Diseases, Maastricht University, Maastricht, The Netherlands, [3]Klinik für Allgemeine und Interventionelle Kardiologie/Angiologie, Herz- und Diabeteszentrum NRW, Universitätsklink der Ruhr-Universität Bochum, Bad Oeynhausen, Germany, [4]Department of Cardiology, Zentralklinik Bad Berka GmbH, Herzzentrum, Department of Cardiology, Bad Berka, Germany, [5]Department of Cardiology, SRH Wald-Klinikum, Gera, Germany, [6]Praxis Dres. Gille/Baulmann, Rheinbach, Germany, [7]Macquarie Medical School, Faculty of Medicine, Health and Human Sciences, Macquarie University, Australia, [8]GROW School for Oncology and Reproduction, Maastricht, The Netherlands

**Background:** Aortic pulse transit time (aPTT) is not constant but fluctuates, which affects the accurate determination of aortic pulse wave velocity (aPWV). In this work, we investigate the influence of age, gender, anthropometric and haemodynamic parameters on aPTT variability determined by the catheter pull-back method.
**Methods**: aPTT could be analysed in 69 patients (61% male, 68 ± 13 years) with femoral catheter access. A stepwise multiple linear regression analysis was performed with aPTT variability as dependent variable and age, gender, BMI, heart rate, aortic systolic blood pressure (aSBP), aortic diastolic blood pressure (aDBP) and variability of aSBP (aSBPV) and aDBP (aDBPV) as predictors. For the dependent haemodynamic variables, only data from the ascending aorta were used.
**Results**: The regression model with the factors heart rate, aSBP, aSBPV, aDBPV achieved the highest goodness of fit of 0.49 (adjusted R-squared). aSBPV and heart rate proved to be the strongest factors (standardised regression coefficient beta 0.397 and 0.301, respectively) followed by 0.258 for aDBPV and -0.199 for aSBP (all $p < 0.05$). The unstandardised regression coefficients B were 0.489 for aSBPV, 0.322 for aDBPV, -0.020 for aSBP and 0.047 for heart rate.
**Conclusions**: Our data show the influence of aortic systolic and diastolic blood pressure variations, heart rate and aSBP on aPTT variability whereas age, gender, and BMI had no significant influence. However, the adjusted R-squared of the model suggests that a considerable part of aPTT variability cannot be explained by the independent variables included in the model.

**Keywords**: PTT variability, PWV variability

### P.003
### Smooth muscle cell-specific knock-out of CTIP2 gene results in aortic hemorrhage

Ara Parlakian[1,2,3], Jocelyne Blanc[1,2,3], Jacqueline GAO-Li[1,2,3], Véronique Regnault[4], Onnik Agbulut[1,2,3], Patrick Lacolley[4], **Zhenlin Li**[1,2,3]

[1]Sorbonne Universite, Paris, France, [2]CNRS UMR8256, Paris, France, [3]INSERM ERL U1164, Paris, France, [4]INSERM—U1116, Nancy, France

**Background:** Ctip2/Bcl11b is a transcription factor with dual action (repression/activation) that couples epigenetic regulation to gene transcription in a variety of physiological responses under healthy and pathological conditions of various tissues. Single nucleotide polymorphisms of Ctip2/Bcl11b gene are associated with a higher susceptibility for aortic stiffness (1). although Ctip2/Bcl11b has been proposed as a crucial regulator of aortic smooth muscle function (2), its mechanism of action in smooth muscle cells is still to be uncovered.
**Methods**: Morphological, cellular and molecular analysis were carried out on the arteries of smooth muscle cell-specific Ctip2-knockout (KO) mice at 3, 7, 28 days after tamoxifen injections.
**Results**: There is no difference between control and mutant mice at the macroscopic level 3 days after Ctip2 KO induction, however, 7 day after Bcl11b inactivation, 65% of the Ctip2-SMKO mice showed signs of hemorrhage in the distal part of the thoracic aorta near the abdominal aorta. The histological examination of thoracic aorta at 7 indicated the presence of "bumpy region" in the mutant aorta. These areas is covered by a thicker layer of extracellular matrix and the presence of IgG positive cells, indicating that cell death is occurring. However, the hemorrhages is contained over time, do not impact mice survival. qPCR analysis indicated the altered expression of circadian-related genes such as genes of Bmal and ciart.
**Conclusions**: Our data indicate the primary effect of Bcl11b inactivation on cell death, probably by necroptosis.

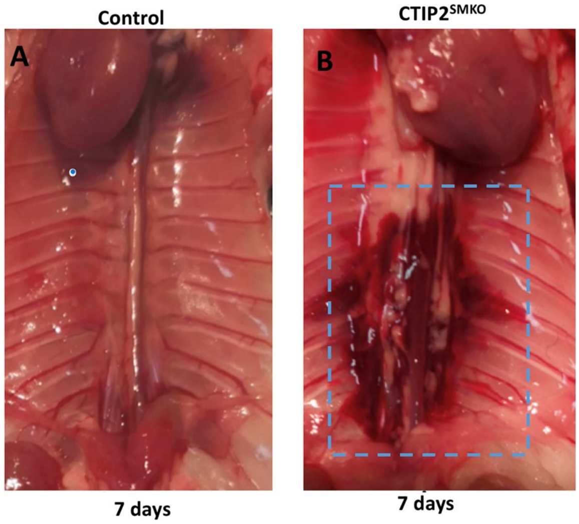

Photos showing thoracic aorta from control (A) and mutant mice (B) at 7 days after the injection of tamoxifen to 3 month-old mice.

**References**
Mitchell GF, Verwoert GC, Tarasov KV et al., Common genetic variation in the 3'-BCL11B gene desert is associated with carotid-femoral pulse wave velocity and excess cardiovascular disease risk: the AortaGen Consortium. Circ Cardiovasc Genet. 2012;5(1):81–90.
Valisno JAC, May J, Singh K, Helm EY, Venegas L, Budbazar E, Goodman JB, Nicholson CJ, Avram D, Cohen RA, Mitchell GF, Morgan KG, Seta F. BCL11B Regulates Arterial Stiffness and Related Target Organ Damage. Circ Res. 2021;128(6):755–768.

**Keywords**: Cell death, CTIP2/Bcl11b, hemorrhage

### P.004
### Smooth muscle integrin αv contributes to the regulation of cell stiffness

Alexandre Raoul[1], Ekaterina Belozertseva, Lei Tian[2], Xiao Liu[3], Caterina Maria Tone[3], Jocelyne Blanc[2], Dario Coletti[2], Daniel Henrion[4], Véronique Regnault[1], Patrick Lacolley[1], Emmanuelle Lacaze[3], Pascal Challande[5], **Dr Zhenlin Li**[2]

[1]INSERM1116, Vandoeuvre-lès-nancy, France, [2]CNRS8256, Paris, France, [3]CNRS7588, Paris, France, [4]INSERM1083 – CNRS6214, Angers, France, [5]CNRS7190, Paris, France

**Background:** Integrin αv is a receptor for adhesion proteins expressed at high density in vascular smooth muscle cells (VSMC) whose phenotypic modulation plays a crucial role in arterial ageing.
**Objectives**: To define the arterial phenotype in mice conditionally inactivated for the integrin αv subunit in VSMC and the role of this integrin in angiotensin II (Ang II)-induced arterial and VSMC stiffness.
**Methods and results**: We used a VSMC specific knock-out αv mouse model induced in adult mice by injection of tamoxifen. Trangenic mice (αvSMKO) and control littermates (Ctrl) were infused with Ang II (1.5 mg/kg/day) for 4 weeks. The pressure effect of Ang II was similar in Ctrl and αvSMKO mice. The carotid distensibility/pressure and elastic modulus/wall stress curves were similar in control and αvSMKO mice, indicating comparable arterial stiffness. Ang II treatment resulted in increased carotid stiffness in both groups without changes in vascular reactivity and myogenic tone. Electronic microscopy revealed less vesicles containing fiber-like materials in the SMCs of Ang II-treated αvSMKO carotids Elastic modulus of cultured VSMCs determined using atomic force microscopy was higher after Ang II treatment in cells from both groups. At baseline and after treatment, elastic modulus was higher in cells from αvSMKO mice than in cells from Ctrl mice.
**Conclusion**: Inactivation of αv-containing integrins on VSMCs increases cell stiffness. The general mechanism involves a cross-talk between extracellular matrix, αv integrins and cytoskeletal complex. The lack of distensibility changes suggests additional changes at the level of αv-mediated dynamics of focal adhesion.

**Keywords**: Integrin αv, vascular smooth muscle cells, cell stiffness

### P.005

### Estrogen modulates phenotypic state of male vascular smooth muscle cells exposed to flow conditions

**Enzo Lecoq**[1], Nathan Wisniewski[1], Leo Jannot[1], Eva Feigerlova[1]

[1]INSERM UMR_S 1116—DCAC Université de Lorraine, Nancy, France

**Although** an estrogen-mediated vasculoprotective effect is widely accepted in premenopausal women, literature data indicate that estrogen therapy in transgender women confer an increased risk of cardiovascular events. Vascular smooth muscle cell (VSMC) reside in a 3-dimensional environment and are not normally exposed directly to the shear stresses of flowing blood in the vascular system, because the endothelial cell layer provides the contacting surface for blood flow. However, in cases of endothelial injury, the superficial layer of SMCs is exposed directly to blood flow shear stresses. We hypothesized that treatment of male VSMCs with estrogens alters cell behavior.
Our aim was to study the effect of shear stress on male VSMCs in a 2D environment under flow model. Cells were treated with 17-β-estradiol and cultured in the Ibidi chamber under laminar flow and shear stress of 1–2 $dyn.cm^{-2}$. The cell orientation and morphology and phenotypic changes were analyzed.
**Results**: We observed an increased expression of MYH10 exposed to shear stress. The expression of MYH10 seems to be correlated with the orientation of VSMCs. The orientation of VSMCs treated with estrogens is parallel to the culture medium flow. Our preliminary results further suggest an increased expression of the MMP-2 under estrogen treatment under flow conditions in the 2D model.
**Conclusion**: The differential effects of laminar flow and shear stress flow may be due to the different phenotypic state of the VSMCs.

**Keywords**: Sex hormones, Laminar flow and shear stress flow, Vascular smooth muscle cell

### Brain

### P.006

### Physiological effects of a biased angiotensin II type 1 receptor agonist on cerebral circulation

**Mélissa Colin**[1,4], Céline Delaitre[1], Sandra Lecat[2], Samir Acherar[3], Sébastien Foulquier[4], Isabelle Lartaud[1], François Dupuis.[1]

[1]Université de Lorraine, CITHEFOR, Vandoeuvre-lès-Nancy, France, [2]Université de Strasbourg, CNRS, Biotechnologie et Signalisation Cellulaire, Illkirch-Graffenstaden, France, [3]Université de Lorraine, CNRS, LCPM, Nancy, France, [4]Maastricht University, School for Mental Health and Neuroscience, Maastricht, The Netherlands

**Background:** The angiotensin II type 1 ($AT_1$) receptor has a relevant role in the physiology and pathophysiology of the cerebrovascular system. Its vasoconstrictor effect, consecutive to Gq protein activation, reduces cerebral perfusion during stroke. In addition, $AT_1$ receptor activity is directly regulated by the β-arrestin pathway, involved in receptor internalization [1]. Recently, the development of biased agonists, able to selectively activate the β-arrestin pathway without Gq activation appears to be a promising new therapeutic strategy in cardiovascular pathologies [2]. In the current project, we explore the impact of an $AT_1$ biased agonist (TRV027) on the regulation of the cerebral circulation.
**Methods**: We evaluated the TRV027 signaling on HEK293-cells overexpressing $AT_1$ using bioluminescence resonance energy transfer (NanoBRET) and calcium mobilization assays. In parallel, concentration–response curves to TRV027 were built on an ex vivo model of isolated and perfused middle cerebral arteries (MCA) by measuring changes in internal diameter.
**Results**: BRET results show that TRV027 induces an activation of the β-arrestin pathway with a maximal increase of BRET ratio of 0.08 while inactivating the Gq pathway. Calcium mobilization assays confirm this Gq inactivation. As expected, results obtained in MCA show no effect of TRV027 on arterial diameter.
**Discussion**: Tracking the $AT_1$ receptor using specific fluorescent tools to follow its internalization (confocal microscopy) is currently under development.
The next step will be to assess in vivo the potential beneficial and protective effects of TRV027 in cerebrovascular pathologies, in collaboration with Maastricht University.

#### References

1. Violin JD, Lefkowitz RJ. β-Arrestin-biased ligands at seven-transmembrane receptors. Trends Pharmacol Sci. août 2007;28(8):416-22.
2. Boerrigter G, Soergel DG, Lark MW, Burnett JC. TRV120027, a Novel Beta-Arrestin Biased Ligand at the Angiotensin II Type I Receptor, Unloads the Heart and Maintains Renal Function When Added to Furosemide in Experimental Heart Failure. J Card Fail. août 2011;17(8):S63-4.

**Keywords**: $AT_1$ receptor; cerebral circulation; β-arrestin; biased agonist

### Imaging technologies

### P.011

### Non-contact Method for Fast Localization of Perforator Arteries

**Valentina Vassilenko**[1,2], Anna Poplavska[1], Diogo Casal[3,4], Edivaldo Junior[1,4]

[1]Nova School Of Science And Technology—Nova University Of Lisbon, Campus FCT UNL, Portugal, [2]Iberian Network on Arterial Structure, Central Hemodynamics and Neurocognition,, Portugal, [3]Plastic and Reconstructive Surgery Department and Burn Unit, Centro Hospitalar de Lisboa Central, Lisbon, Portugal, [4]Anatomy Department, Nova Medical School—Nova University of Lisbon, Lisbon, Portugal

**Recently** an extensive clinical experience in flap surgery has confirmed that its success depends on the correct identification of vital perforator vessels [1]. Unfortunately, the perforator vessels frequently have a variable location. So, the knowledge about perforator anatomy during preoperative planning is one of the most critical factors.
In this work, we present the method and experimental results for non-contact and fast localization of the cutaneous perforators using Infrared Thermography (IRT). This imaging technique can provide real-time information on skin perfusion by measuring body surface temperature. Validation of the method was performed against the ultrasound technology realized in the hand-held Doppler flowmeter, which is widely used in most hospitals and is an essential tool where a rapid analysis of the vascular status of a patient is routine.
Both technics were applied in this work for the identification of forearm cutaneous perforator vessels. The reflection of sound waves, predominantly from intravascular blood flow of the forearm, was registered by a hand-held BT-200 V® Vascular Doppler pan. The infrared images were obtained by two cameras: FLIR® E6 with temperature sensitivity $< 0.06$ °C and ($320 \times 240$)-pixel display resolution, and Thermal Expert with sensitivity $< 0.05$ °C and array format $640 \times 480$. Perforator mapping of the forearm area ware compared for accuracy, timing, and the operator's skills.
Obtained results show that IRT images provide valuable real-time information on the hemodynamic quality of perforators and their accurate location. Its potential to reveal underlying perforator vessels may also be used for postoperative monitoring of flap perfusion [2].

**References**
[1] D. Casal et al., "A Model of Free Tissue Transfer: The Rat Epigastric Free Flap," J. Vis. Exp., no. 119, pp. 14–16, 2017.
[2] T. Sjøberg, J. B. Mercer, S. Weum, and L. de Weerd, "The Value of Dynamic Infrared Thermography in Pedicled Thoracodorsal Artery Perforator Flap Surgery," Plast. Reconstr. Surg. – Glob. Open, vol. 8, no. 7, 2020.

**Keywords**: Cutaneous Perforator Vessels (CPVs), Infrared Thermography (IRT), hand-held Doppler devices (HHDD), perforator mapping

### P.013

### Radial artery phenotyping in systemic sclerosis through ultra-high frequency ultrasound: a radiomic approach

**Federica Poli**[1], Goncalo Boleto[2], Hakim Khettab[3], Pierre Boutouyrie[1,3], Yannick Allanore[2], Rosa Maria Bruno[1,3]

[1]Paris Cardiovascular Research Center (PARCC)-INSERM UMR-970, Paris, France, [2]Department of Rheumatology, Université de Paris, Cochin Hospital, Paris, France, [3]AP-HP, Hopital Européen Georges Pompidou, Université de Paris, Paris, France

**Background:** Systemic sclerosis (SSc) is a disorder characterized by a massive vascular involvement. Imaging biomarkers of vascular involvement in SSc may have potential clinical implications for prediction of the pathogenesis of vascular complications [1]. This study is aimed at identifying possible patterns of vascular wall disarray and remodeling in radial arteries of SSc patients, by means of ultrahigh frequency ultrasound (UHFUS).
**Methods**: 5 end-diastolic frames of the right radial arteries of 41 patients with SSc and 41 healthy controls were obtained by VevoMD (70 MHz probe, FUJIFILM, VisualSonics, Toronto, Canada). 74 radiomic features and 4 engineered parameters were extracted: inner and outer layer thickness, and presence of adjunctive acoustic interfaces (triple signal). A feature selection algorithm was applied to reduce the number of features. The selected features were used to train classification model, using Linear Support Vector Machine (SVM).
**Results**: The SVM classification model showed good performance (sensitivity = 0.63, specificity = 0.88, accuracy = 0.75, AUC = 0.75) to discriminate SSc patients from controls using sixteen selected features. Inner layer (208 ± 61 vs 179 ± 47 μm, p = 0.04) and outer layer thickness (104 ± 22 vs 120 ± 36 μm, p = 0.03) were significantly higher in SSc than in controls, triple signal pattern more frequent in patients (p = 0.002).
**Conclusions**: Wall ultrastructure of radial arteries of SSc patients is altered: inner and outer layer thickened, showing frequently a triple signal pattern. Radiomic approach allow to distinguish between radial images from SSc patients and controls with a 75% accuracy.

**Reference**
1. Allanore Y, Distler O, Matucci-Cerinic M, Denton CP. Review: Defining a Unified Vascular Phenotype in Systemic Sclerosis. Arthritis Rheumatol Hoboken NJ. febbraio 2018;70(2):162–70.

**Keywords**: UHFUS, radial arteries, radiomic analysis, machine learning

### P.014 Cardiovascular adaptation to strenuous exercise: exploring the complexity of the arterial protective role for the heart

**Maria Raffaella Martina**[1], Elisabetta Bianchini[1], Marco Scalese[1], Nicole Di Lascio[1], Rosa Maria Bruno[2], Lorenza Pratali.[1]

[1]Institute of Clinical Physiology—Italian National Research Council (CNR-IFC), Pisa, Italy, [2]Université Paris Cité, Inserm, PARCC, F-75015, Paris, France

**Background:** The intense exercise effect evaluation on the cardiovascular system can help to profile and reduce the risks (1–3). This work aims at assessing cardiovascular adaptation in runners by a multi-site non-invasive approach.
**Methods**: 49 runners (A) trained for 8.5 ± 8.9 years, 3.9 ± 1.4 days/week and 15 sedentary (S) subjects matched by sex, age, BMI, baseline brachial pressures and heart rate underwent ultrasound semi-automatic assessment of the vascular system (arterial mean diameters, MD, and distensibility, DC, of abdominal aorta, common carotid, common femoral, brachial artery) and of cardiac parameters. Central pressure-based (applanation tonometry) hemodynamic properties according to the reservoir theory were derived.
**Results**: Cardiac parameters related to dimensions, mass and volumes showed significantly higher values in A compared to S (A/S: Left Ventricular Internal diameter, 29 ± 3 mm/27 ± 3 mm; Left Ventricular Mass, 161 ± 31 g/141 ± 25 g; Aortic root size 30 ± 3 mm/27 ± 2 mm; Stroke Volume, 76 ± 13 mL/69 ± 16 mL; Arterial Elastance, 7 ± 1 mm $HgmL^{-1}$/6 ± 1 mm $HgmL^{-1}$).
MD was greater in each large arterial site in A than in S reporting a trend in the carotid and significant differences in aorta and femoral artery (aorta: 16 ± 2 mm/13 ± 1 mm; femoral: 10 ± 1 mm/9 ± 1 mm). DC evidenced a lowering trend in A for each arterial site except for the brachial artery. Hemodynamic parameters showed higher reservoir pressure in A compared to S (Pressure reservoir integral, 14 ± 4/11 ± 3).
**Conclusions**: Strenuous exercise induced a well-known cardiac remodeling which can be hypothesized to be slower in the arterial tree because of highly differentiated and complex mechanism aiming to heart protection. Accordingly, the increased reservoir pressure in runners could be interpreted as sentinel parameter of vascular "fatigue".

**References**
1. Heffernan KS, Yoon ES, Sharman JE, Davies JE, Shih YT, Chen CH, et al. Resistance exercise training reduces arterial reservoir pressure in older adults with prehypertension and hypertension. Hypertens Res. 2013;36(5):422–7.
2. Ramos JS, Ramos M V., Dalleck LC, Borrani F, Walker KB, Fassett RG, et al. Fitness Is Independently Associated with Central Hemodynamics in Metabolic Syndrome. Med Sci Sports Exerc. 2016;48(8):1539–47.
3. Ujka K, Bruno RM, Bastiani L, Bernardi E, Sdringola P, Dikic N, et al. Relationship between occupational physical activity and subclinical vascular damage in moderate-altitude dwellers. High Alt Med Biol. 2017;18(3):249–57.

**Keywords**: Cardiovascular adaptation, exercise, ultrasound

### P.015

### Sex-related differences in skin microvascular function of healthy normotensive individuals as assessed with laser speckle contrast imaging

**Antonios Lazaridis**[1], Konstantinos Mastrogiannis[1], Anastasia Malliora[1], Stamatina Lamprou[1], Nikolaos Koletsos[1], Barbara Nikolaidou[1], Panagiotis Dolgyras[1], Areti Triantafyllou[1], Eugenia Gkaliagkousi[1]

[1]3rd Department of Internal Medicine, Papageorgiou General Hospital, Aristotle University of Thessaloniki, Thessaloniki, Greece

**Background:** Skin microcirculation is considered a window to assess generalized microvascular function. Changes in skin microvascular function (SMF) have been identified in several cardiovascular disease states. However, scarce data exists regarding SMF in healthy adults and the impact of gender on it [1,2]. In this study, we assessed SMF in healthy individuals with the dynamic technique Lase Speckle Contrast Imaging (LSCI).
**Methods**: Healthy normotensives were included in the study. Office blood pressure (BP) was measured according to standard guidelines. SMF was assessed with LSCI (PeriCam PSI NR, Perimed, Sweden) coupled with post-occlusive reactive hyperemia (PORH). Results were expressed as perfusion during baseline, occlusion and peak period (arbitrary Perfusion Units,PUs), time until maximal perfusion (sec), the percentage increase of perfusion between baseline and peak period

(%) and PORH amplitude calculated as peak cutaneous vascular conductance (CVC) – baseline CVC. CVC was calculated as mean perfusion during each PORH period divided by mean BP (LSPUs/mmHg).
**Results**: We studied 86 healthy normotensives including 50 women and 36 age-matched men. Body mass index, creatinine, office systolic BP (SBP) and diastolic BP (DBP) were significantly higher in men compared to women. Regarding SMF, perfusion during baseline and occlusion, baseline CVC, peak CVC and PORH amplitude ($0.89 \pm 0.21$ vs $0.75 \pm 0.19$, $p < 0.01$) were significantly higher in females compared to males. In addition, PORH was negatively associated with office SBP ($r = -0.258$, $p < 0.05$).
**Conclusions**: Healthy females present significantly higher SMF parameters compared to age-matched males. Further research is needed to clarify the impact of gender on microvascular function and its further implications.

#### References

[1] Stupin A, Stupin M, Baric L, Matic A, Kolar L and Drenjancevic I. Sex-related differences in forearm skin microvascular reactivity of young healthy subjects. Clin Hemorheol Microcirc. 2019;72(4):339–351.
[2] Hodges G, Sharp L, Clements R, Goldspink R, George K, and Cable N. Influence of age, sex, and aerobic capacity on forearm and skin blood flow and vascular conductance. Eur J Appl Physiol. 2010;109(6):1009–15.

**Keywords**: Microcirculation, laser speckle contrast imaging, perfusion, skin microvascular function

### P.021

### Longitudinal and radial distensibility of the ascending aorta in aging and aortic valve stenosis using MRI feature tracking

**Marie Shannon Soulez**[1,2], Jérôme Lamy[3], Vincent Nguyen[1], Umit Gencer[4], Gilles Soulat[4], Elie Mousseaux[4], Emilie Bollache[1], Nadjia Kachenoura[1]

[1]Sorbonne Université, CNRS, INSERM, Laboratoire d'Imagerie Biomédicale, LIB, Paris, France, [2]Université Paris-Est Créteil, Creteil, France, [3]Department of Radiology and Biomedical Imaging, Yale University, New Haven, USA, [4]PARCC, Université de Paris, Inserm/AP-HP, Hôpital Européen Georges Pompidou, Paris, France

**Background:** We aimed to test the feasibility of MRI-based feature tracking (FT) to measure longitudinal strain and radial motion fraction of the proximal ascending aorta (AA) and to investigate how these measures are affected by aging and by the presence of calcified aortic valve stenosis (AVS).
**Methods**: Twenty healthy volunteers (HV) < 40 years ($29 \pm 1.6$ years, 10 males), 20 HV ≥ 40 years ($58 \pm 1.5$ years, 10 males) and 31 patients with AVS ($73 \pm 1.6$ years, 20 males) underwent 2D cine thoracic aortic MRI in sagittal and axial views immediately followed by carotid artery applanation tonometry. AA anterior wall (Figure a) was semi-automatically tracked on sagittal images throughout the cardiac cycle to estimate longitudinal strain and radial motion fraction peaks, while using custom FT software [1], which was previously dedicated to multi-chamber strain evaluation in the heart. Conventional global AA strain was also measured on axial views based on cross-sectional area [2]. Finally, distensibility was derived as strain/central pulse pressure.
**Results**: Axial (Dist-axial: $R = -0.82$, $p < 0.0001$) and sagittal (radial DistR-sagittal: $R = -0.54$, $p = 0.0004$, longitudinal DistL-sagittal: $R = -0.37$, $p = 0.02$) distensibility measures decreased significantly with age and even more in the presence of AVS (Figure b). When investigating the ability of distensibility measures to discriminate HV from patients, newly proposed DistR-sagittal (0.84) and DistL-sagittal (0.92) demonstrated higher area under the ROC curve than Dist-axial (0.81).
**Conclusions**: MRI FT revealed that age has a stronger impact on AA axial distensibility, while longitudinal distensibility could be more sensitive to the effect of AVS probably because of valvular calcifications that limit this longitudinal motion specifically.

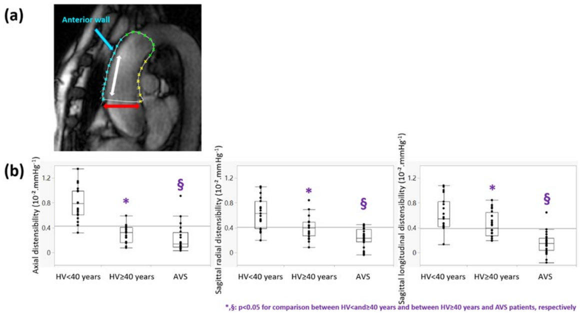

(a): AA longitudinal (white) and radial (red) strain from sagittal MRI
(b): Dist-axial (left), DistR-sagittal (middle) and DistL-sagittal (right) according to subject group: HV < 40 years, HV ≥ 40 years and AVS patients.

#### References

[1] Lamy J, Soulat G, Evin M, Huber A, de Cesare A, Giron A, et al. Scan-rescan reproducibility of ventricular and atrial MRI feature tracking strain. Comput Biol Med. 2018; 92:197–203. https://doi.org/10.1016/j.compbiomed.2017.11.015.
[2] Redheuil A, Yu WC, Wu CO, Mousseaux E, de Cesare A, Yan R, Kachenoura N, Bluemke D, Lima JA. Reduced ascending aortic strain and distensibility: earliest manifestations of vascular aging in humans. Hypertension. 2010 Feb;55(2):319–26. https://doi.org/10.1161/HYPERTENSIONAHA.109.141275.

**Keywords**: MRI, Aortic strain, Aging, Aortic valve stenosis

### Hypertension

### P.022

### Exploring strain-dependent collagen degradation as a driver of hypertension-induced arterial remodelling in lean ZSF1 rats: a pilot study

**Koen van der Laan**[1,2], Koen Reesink[1,2], Sara Lambrichts[3,4], Laura van der Taelen[1,3], Nicole Bitsch[1], Tammo Delhaas[1,2], Sebastien Foulquier[1,3,4], Bart Spronck[1,2,5]

[1]Carim, University Maastricht, Maastricht, Netherlands, [2]Department of Biomedical Engineering, Maastricht University medical centre, Maastricht, Netherlands, [3]Department of Pharmacology & Toxicology, Maastricht University, Maastricht, Netherlands, [4]MHENS School for Mental Health and Neuroscience, Maastricht University, Maastricht, Netherlands, [5]Macquarie Medical School, Faculty of Medicine, Health and Human Sciences, Macquarie University, Sydney, Australia.

**Background:** Hypertension-induced arterial remodelling involves e.g., increased wall thickness, changes in collagen structure and increased collagen content [1]. Normally, collagen is degraded and deposited in 8–10 week turn-over cycles [2]. Collagen fibre strain significantly influences enzymatic degradation efficiency, where straightened but not overstretched fibres experience minimum degradation (Fig. A) [3]. In a homeostatic situation where collagen experiences minimum degradation at mean arterial pressure, fibres would be optimally strained [4]. The onset of hypertension overstretches collagen (1 in Fig. B), accelerating collagen degradation [4]. We hypothesise that optimally strained fibres will gradually make up the bulk of collagen during hypertension, as overstrained collagen degrades faster. This implies that, as collagen returns to the optimal level of strain, increased collagen content is required to maintain luminal diameter and avoid dilatation (2 in Fig. B). We evaluated this hypothesis, expecting hypertensive rat aorta to exhibit less collagen strain under normotensive conditions compared to normotensive control (3 in Fig. B).

**Methods**: One normotensive (NT, Wistar) and one hypertensive (HT, ZSF1 lean [5]) 22-week-old rat abdominal aorta were stretched to in vivo-like length and pressurised to 100 mmHg. Three-dimensional collagen structure was then imaged by second harmonic generation using a two-photon microscope.

**Results**: At 100 mmHg, the HT artery visibly displayed a thicker collagen layer and curlier collagen fibres than the NT artery (Fig. C-F), suggesting lower collagen fibre strain at normotensive pressures (3 in Fig. B).

**Conclusions**: Strain-dependent collagen degradation may be a key process driving hypertension-induced arterial remodelling.

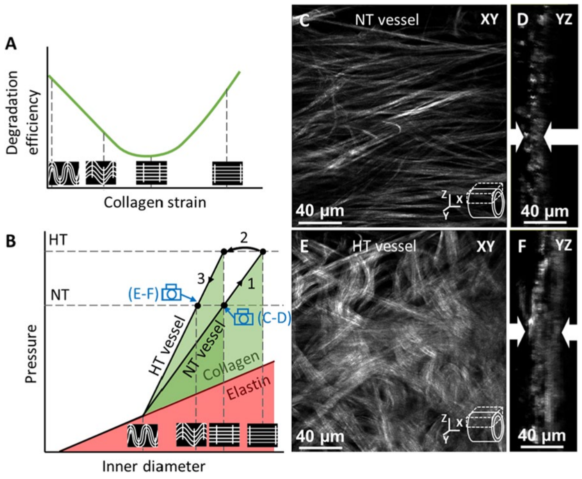

A: Straight-but-not-overstretched collagen experiences minimal degradation [3]. B: Diameter maintenance in hypertension requires additional collagen at minimum-degradation strain. More and curlier collagen in hypertension (E–F) than normotension (C-D).

**References**

1. Intengan and Schiffrin. Vascular remodeling in hypertension. Hypertension. 2001;38:581–587.
2. Nissen et al. Increased turnover of arterial collagen in hypertensive rats. Proc Natl Acad Sci USA. 1978;75:451–453.
3. Gaul et al. Strain mediated enzymatic degradation of arterial tissue. Acta Biomater. 2018;77:301–310.
4. Gaul et al. Pressure-induced collagen degradation in arterial tissue as a potential mechanism for degenerative arterial disease progression. J Mech Behav Biomed Mater. 2020;109:103,771.
5. Hamdani et al. Myocardial titin hypophosphorylation importantly contributes to heart failure with preserved ejection fraction in a rat metabolic risk model. Circ Heart Fail. 2013;6:1239–1249.

**Keywords**: Remoddeling strain collagen degradation

### P.024

### Effects of Nitroglycerin Induced Vasodilation on Elastic versus Muscular Artery Stiffness in Older Veterans

<u>**Ryan Pewowaruk**</u>[1], Amy Hein[1], Claudiq Korcarz[2], Adam Gepner.[1]

[1]William S. Middleton Memorial Veterans Hospital, Madison, United States, [2]University of Wisconsin School of Medicine and Public Health, Madison, United States

**Background:** Vascular smooth muscle tone may play an important role in the physiology of increased arterial stiffness that occurs with aging. This study evaluated the impact of smooth muscle tone on arterial stiffness in older individuals following nitroglycerin induced vasodilation in elastic and muscular arteries.

**Methods**: 40 older Veterans (> 60 years old), without known cardiovascular disease, were included in this study. 20 were hypertensive (70.8 ± 6.6 years, 10 female) and 20 were normotensive controls (72.0 ± 9.3 years, 8 female). Nitroglycerin (NTG) induced changes in arterial stiffness were measured locally with vascular ultrasound in the carotid and brachial arteries, and regionally by carotid-femoral pulse wave velocity (cfPWV) by tonometry.

**Results**: With NTG, both hypertensive and normotensive control Veterans showed increased carotid PWV (6.4 ± 1.3 m/s to 7.2 ± 1.4 m/s, Δ 0.8 ± 1.1 m/s, p = 0.007) and cfPWV (8.6 ± 1.9 m/s to 9.5 ± 2.4 m/s, Δ 0.9 ± 2.3 m/s, p = 0.020) but did not change brachial PWV (11.2 ± 2.4 m/s to 11.1 ± 2.2 m/s, Δ −0.2 ± 2.5 m/s, p = 0.72). The carotid artery dilated more in control participants than hypertensive Veterans (Δ 0.54 ± 0.19 mm vs 0.42 ± 0.12 mm, p = 0.022). Brachial artery dilation was similar, (Δ 0.55 ± 0.26 mm vs 0.51 ± 0.20 mm, p = 0.46).

**Conclusion**: In older Veterans, without known cardiovascular disease, NTG induced vasodilation increased elastic artery stiffness and did not change muscular artery stiffness. Increased central arterial stiffness and reduction in the arterial stiffness gradient could offset some of the benefits of lowering blood pressure in older patients who are prescribed vasodilators as an antihypertensive therapy. Elastic artery stiffening with vasodilation warrants further investigation as it may be important for antihypertensive medication selection and influence CVD development.

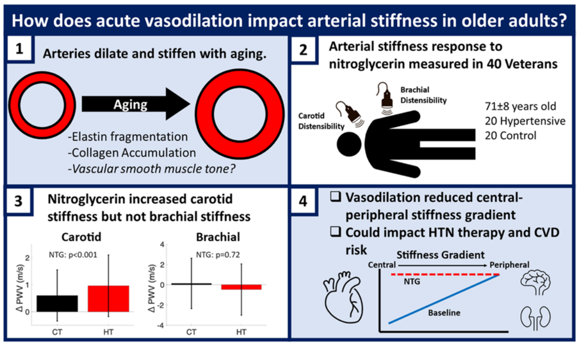

Graphic abstract overviewing 1. research question, 2. methods, 3. results, and 4. clinical implications of findings

**Reference**

Pewowaruk RJ, Gepner AD. Smooth muscle tone alters arterial stiffness: the importance of the extracellular matrix to vascular smooth muscle stiffness ratio. J Hypertens. 2022 Mar 1;40(3):512–519.

**Keywords**: Vascular stiffness, hypertension, vasodilation, smooth muscle

### P.025

**Performance of pOpmetre® versus SphygmoCor® to detect central arterial stiffness using central aortic pressures**

**Mathilde Laime**[1], Hasan Obeid[2], Imad Abi-Nasr[2], Hakim Khettab[3], Magid Hallab[2], Pierre Boutouyrie[3], Loukman Omarjee[4,5]

[1]University Bretagne Occidentale, Laboratoire ORPHY, Brest, France, [2]Clinique Bizet, Cardiology unit, Paris, France, [3]Pharmacology department, European Georges Pompidou Hospital and Assistance Publique Hôpitaux de Paris, Paris Cardiovascular Research Center (PARCC), Paris, France, [4]Univ Rennes, CHU Rennes, INSERM CIC1414, Vascular Medicine Department, Rennes, France, [5]Vascular Medicine Department, Redon Hospital, 35600, Redon, Redon, France

Central arterial stiffness is an independent predictor of cardiovascular and total mortality. It can be diagnose directly by aortic pulse wave velocity (aPWV) or indirectly by central aortic pressures (CAP). SphygmoCor®, a non-invasive device using applanation tonometry, is the gold standard to measure CAP. However, its complexity limits its use in clinical practice.

The aim of this study is to evaluate the accuracy of a novel non-invasive device, pOpmètre® (Axelife SAS, Saint-Nicolas de Redon, France), to measure CAP in suspected peripheral arterial disease (PAD) patients.

Systolic, diastolic and central pulse pressures measured with pOpmètre® are compared with those measured with SphygmoCor®.

In this pilot, monocentric, prospective study, 53 suspected PAD patients were included. Among them: i) 26 patients (age: $69 \pm 10$y; 65% men) were diagnosed with peripheral arterial obstructive disease (PAOD) defined by a toe-brachial index $\leq 0.7$ and/or an ankle-brachial index (ABI) $\leq 0.9$; ii) 10 patients (age: $74 \pm 9$y; 70% men) were diagnosed with peripheral arterial stiffness (mediacalcosis) defined by an ABI $> 1.4$; iii) 20 patients (age: $55 \pm 16$; 40% men) without PAD diagnosis. There was a significant correlation between systolic, diastolic and central pulse pressures measured by pOpmètre® compared with those measured by SphygmoCor® in PAD patients (respectively, R2 = [0.94, 1.00, 0.84]; p = [10e − 16, 10e − 16, 10e − 11]), peripheral arterial stiffness patients (respectively, R2 = [0.96, 1.00, 0.84]; p = [10e − 8, 10e − 13, 10e − 7]) and no PAD patients (R2 = [0.98, 1.00, 0.97]]; p = [10e − 12, 10e − 16, 10e − 8]).

CAP assessed by pOpmètre® could be used in clinical practice to detect central arterial stiffness in suspected PAD patients.

**Reference**

McEniery CM, Cockcroft JR, Roman MJ, Franklin SS, Wilkinson IB. Central blood pressure: current evidence and clinical importance. Eur Heart J. 7 juill 2014;35(26):1719-25.

**Keywords**: Central aortic pressure, Peripheral arterial disease

### P.027

**Analyses of pulsatile pulmonary hemodynamics and right ventricular function during exercise**

**Christopher Lechuga**[1], Farhan Raza[1], Mitchel J. Colebank[1], Naomi C. Chesler.[1]

[1]University of California, Irvine, Irvine, United States

**Background:** Pulmonary hypertension (PH) leads to a mismatched right ventricular (RV)-pulmonary arterial (PA) relationship (uncoupling), which increases mortality [1]. Current diagnostic strategies utilize pulmonary vascular resistance (PVR) [2] but disregard the opposition to pulsatile flow as well as response to exercise [3,4]. We hypothesize that pulsatile pulmonary hemodynamics during exercise and their relationship to RV-PA coupling can differentiate PH phenotypes and refine diagnoses.

**Methods**: 13 adult subjects with precapillary PH (PAH; n = 5), isolated postcapillary PH (Ipc-PH; n = 5), or no PH (No PH; n = 1) performed invasive cardiopulmonary exercise testing with echocardiography-based pulmonary vascular pressure-flow and catheter-based RV pressure–volume data collection. Characteristic impedance ZC, effective arterial elastance, Ea, and end-systolic elastance, Ees were computed during rest, exercise, and recovery.

**Results**: At rest, subjects with Ipc-PH or No PH tended to have lower ZC and Ea than those with PAH (Fig. 1A&C); all Ees values were similar (Fig. 1b). During exercise, Zc decreased in the subject with No PH, whereas it increased in those with Ipc-PH and did not change in those with PAH. During exercise, both Ees and Ea increased for all subjects but the increase in Ea was larger than the increase in Ees for both the PAH and Ipc-PH groups, suggesting RV:PA uncoupling. Interestingly, the changes in ZC and Ees/Ea were inversely related in Ipc-PH during exercise.

**Conclusions**: With the limitation that the sample size is small, our findings suggest that analysis of pulsatile pulmonary hemodynamics and RV:PA coupling with exercise can reveal distinctive PH phenotypes and have diagnostic value.

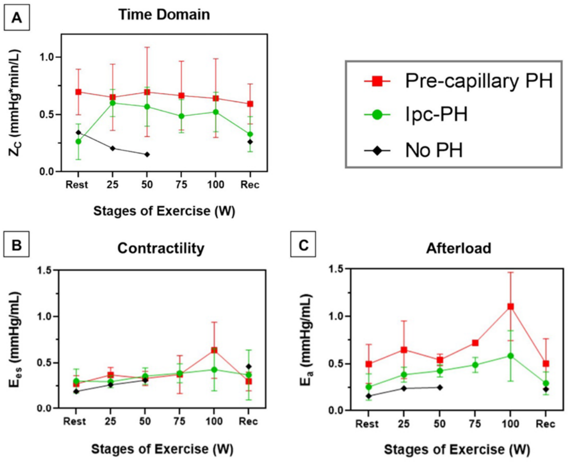

**Figure 1** *A. Average pulmonary vascular characteristic impedance ($Z_C$) data in the time domain, B. average end-systolic elastance ($E_{es}$; i.e. RV Contractility) data, and C. average effective arterial elastance ($E_a$; i.e. RV Afterload) data for No PH (n = 1), pre-capillary PH (n = 5), and Ipc-PH (n = 5) at rest, during exercise, and at recovery.*

**References:**

[1] Rosenkranz, S et al., Circulation, 141(8):678–693, 2020.
[2] Al-Omary, MS et al., Hypertension, 75(6):1397–1408, 2020.
[3] Vanderpool, RR et al., Annals of Biomedical Engineering, 38(5):1854–1861, 2010.
[4] Sabbahi, A et al., Expert Review of Respiratory Medicine, 14(3):317–327, 2020.

**Keywords**: Impedance, Coupling, Hypertension, Exercise.

### Obesity, metabolic disorders and cardiovascular disease

### P.030

**Relationship between aortic and carotid stiffness with measures of adiposity in adolescents. The maciste study**

**Giacomo Pucci**[1,2], Mariella Martina[3], Elisabetta Bianchini[3], Vincenzo Gemignani[3], Marco D'Abbondanza[1,2], Rosa Curcio[1,2], Francesca Battista[4], Fabio Anastasio[5], Gaetano Vaudo[1,2]

[1]Department of Medicine and Surgery—University of Perugia, Perugia, Italy, [2]"Santa Maria" University Hospital, Terni, Italy, [3]Institute of Clinical Physiology—National Council of Research, Pisa, Italy, [4]Sports Medicine—University of Padua, Padua, Italy, [5]Cardiology Division—Mondovì Hospital, Cuneo, Italy

**Background:** We evaluated the differential association between arterial stiffness, taken at different arterial segments (aortic and carotid), with global and local measures of adiposity, accounting for BP as a mediator of the relationship between fat accumulation and increased arterial stiffness.

**Methods**: 322 healthy Italian adolescents from the MACISTE Study (Metabolic and Cardiovascular Investigation at School, TErni), were evaluated. BMI, waist, hip and neck circumferences (NC) were taken as measures of adiposity. Laboratory measures of adiposity were also collected. Arterial stiffness was measured through carotid-femoral pulse wave velocity (applanation tonometry, SphygmoCor), and at the carotid level (Cardiovascular suite, Quipu, Italy).
**Results**: Mean age 17 ± 1.4 years, 56% boys, 40 (12%) with overweight. All central and peripheral measures of BP were higher in overweight vs normoweight (all $p < 0.01$) excluding peripheral and central DBPs, which were lower in overweight. The aortic-to-brachial pulse pressure amplification was reduced in overweight vs normoweight (1.51 ± 0.13 vs 1.58 ± 0,13, $p < 0.01$). Carotid and aortic stiffness were positively correlated with anthropometric and laboratory measures of adiposity. After adjustment for MAP, only NC remained associated with carotid ($\beta = 0.24$, $p < 0.01$) and aortic stiffness ($\beta = 0.16$, $p = 0.02$). After adjustment for central PP, only carotid ($\beta = 0.15$, $p = 0.04$), but not aortic stiffness ($\beta = 0.12$, $p = 0.07$) was associated with NC.
**Conclusions**: Arterial stiffness, when assessed at different levels of central arteries, showed site-specific associations with measures of body fat adiposity. NC was the only measure of adiposity to show a BP-independent association with carotid stiffness. Carotid stiffness is a promising marker of pressure-independent vascular damage promoted by overweight status.

**Keywords**: Adolescents, adiposity, carotid stiffness, blood pressure

### P.031

### Obesity and Cardiovascular Risk Factors in the Outcome of Arterial Stiffness

Gabriela Portugal[1], **Mariana Mendes**[1], Laysa Rebouças[1], Lucelia Magalhães

[1]Uniftc, Salvador, Brazil

**Obesity** is one of the biggest health problems in the world. It is constituted as the second most important risk factor for the development of chronic non-communicable diseases. In this sense, it is assumed that the increase in arterial stiffness is on the path between obesity and cardiovascular diseases. The objective of this study was to evaluate the correlation between obesity and cardiovascular risk factors with arterial stiffness in patients treated at a teaching clinic in the university center, Salvador-Ba, in 2022. This is an observational, cross-sectional and analytical study. The studied population comprised individuals residing in the Valley of Ogunjá neighborhood, Acupe in Brotas, both sexes, over 18 years of age and obese. The indicators of obesity were: waist circumference (women > 88 cm and men > 90 cm), cervical (> 34 cm in women and > 37 cm in men), waist-hip ratio (> 0.80 in women and > 0.95 in men), body mass index (BMI) (> 30 kg/m$^2$) and evaluation of the carotid-femoral pulse wave velocity (PWV) (> 10 m/s). The results obtained denote a direct to statistically significant linear correlation: waist circumference indicator associated with PWV (p value 0.055), waist-hip ratio (p-value 0.003), cervical circumference (p-value 0.004). Only the BMI indicator associated with PWV (p value 0.584) was not statistically significant. It is concluded that the indicators of abdominal and cervical circumference and waist-hip ratio obtained statistical significance when attributed to PWV and can be used as indicators of arterial stiffness.

#### References

Associação Brasileira para o Estudo da Obesidade e Síndrome Metabólica. Mapa da Obesidade [internet]. Higienópolis: 2021 [citied: Out 24 2021]. Available from: https://abeso.org.br/obesidade-e-sindrome-metabolica/mapa da-obesidade/.

Brasil. Saúde prepara ações para controle do excesso de peso e da obesidade [Internet]. Brasília: Ministério da Saúde,2020 [cited: Out 24 2021]. Available from: https://aps.saude.gov.br/noticia/10137#:~:text=O%20Minist%C3%A9rio%20da%20Sa%C3%BAde%20ir%C3%A1,de%20promo%C3%A7%C3%A3o%20de%20da%20sa%C3%BAde.

Bessa LR, Cruz LAB, Lima RLS, Presta MCLF, Alves Filho AAO, Cunha, RCA, et al. Correlation between Neck Circumference and Pulse Wave Velocity: A Population-based Study. Rev Artery Research 2020.

Instituto Brasileiro de Geografia e Estatística (IBGE). CensoIBGE 2010. Brazil: IBGE; 2010.

**Keywords**: Obesity, Pulse wave speed, Arterial stiffness

### P.032

### In vivo measurement of blood pressure and pulse wave velocity in streptozotocin-induced type 1 diabetes in mice

**Margarita G. Pencheva**[1,2], Eline Berends[1,3], Peter Leenders[3,4], Myrthe M. van der Bruggen[1,2], Koen W.F. van der Laan[2], Alessandro Giudici[2,5], Koen D. Reesink[2], Sébastien Foulquier[3], Bart Spronck[2,6], Casper G. Schalkwijk[1]

[1]Depatrment of Internal Medicine, CARIM School for Cardiovascular Diseases, Maastricht University, Maastricht, The Netherlands, [2]Dept. of Biomedical Engineering, CARIM School for Cardiovascular Diseases, Maastricht University, Maastricht, The Netherlands, [3]Dept. of Pharmacology and Toxicology, CARIM School for Cardiovascular Diseases, Maastricht University, Maastricht, The Netherlands, [4]Dept. of Biochemistry, CARIM School for Cardiovascular Diseases, Maastricht University, Maastricht, The Netherlands, [5]GROW School for Oncology and Reproduction, Maastricht University, Maastricht, The Netherlands, [6]Macquarie Medical School, Faculty of Medicine, Health and Human Sciences, Macquarie University, Sydney, Australia

**Background:** In humans, type 1 diabetes (T1D) is associated with arterial stiffening as assessed by carotid-femoral pulse wave velocity (cfPWV) [1]. To experimentally study the underlying mechanism of this stiffening, we investigated blood pressure (BP) and cfPWV in streptozotocin (STZ)-induced diabetes in mice.
**Methods**: Twenty-four 9-week-old male C57BL/6 J mice were divided equally among diabetic (induced through once-daily 50 mg/kg STZ injections for five days) and control (sham injections using citrate buffer) groups, and were kept to an age of 24 weeks. Fasting glucose was measured every 4–5 weeks via tail blood collection with levels of 15 mmol/L and higher considered diabetic. Non-invasive tonometric cfPWV was measured in anaesthetised animals (1% isoflurane) 24 h prior to euthanasia; tail-cuff BP was measured directly prior to euthanasia.
**Results**: Diabetic mice exhibited higher fasting glucose than controls ($p < 0.0001$, two-way ANOVA with Tukey post-hoc test; Fig. A). There was no difference in systolic BP (110 ± 4 vs. 104 ± 3 mmHg, $p = 0.26$, mean ± SE, unpaired t-test) and cfPWV (2.60 ± 0.14 vs. 2.55 ± 0.11 m/s, $p = 0.80$) between diabetic and control mice (Fig. B-C).
**Discussion**: In the popular animal model of STZ-induced T1D, existing literature on systolic BP is not consistent [2,3]. Literature about cfPWV is limited: in contrast to our data, one report showed an STZ-induced increase in ultrasound-derived cfPWV [4]. Discrepancies between studies could be due to different methods of measuring BP and cfPWV [5]; the choice of measurement methods therefore needs critical appraisal.
**Conclusion:** In the murine model of STZ-induced T1D, we did not find elevated BP or increased arterial stiffness.

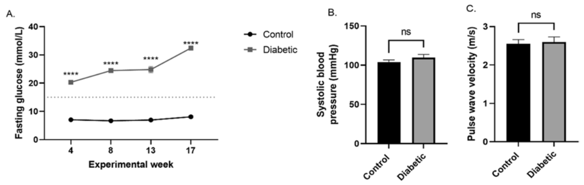

**Despite** clearly increased fasting blood glucose (A), streptozotocin-induced diabetic mice did not show increased blood pressure (B) or arterial stiffness (C). Shown are mean ± SE; ****$p < 0.0001$; ns, not significant.

**References:**

1. Tougaard NH, Theilade S, Winther SA, Tofte N, Ahluwalia TS, Hansen TW, et al. J Am Heart Assoc. 2020;9:e017165.
2. Gurley SB, Clare SE, Snow KP, Hu A, Meyer TW, Coffman TM. Am J Physiol Renal Physiol. 2010;298:F788-795.
3. Zhong F, Chen H, Wei C, Zhang W, Li Z, Jain MK, et al. Kidney Int. 2015;87:382–395.
4. Heath JM, Sun Y, Yuan K, Bradley WE, Litovsky S, Dell'Italia LJ, et al. Circ Res. 2014;114:1094–1102.
5. Leloup AJ, Fransen P, Van Hove CE, Demolder M, De Keulenaer GW, Schrijvers DM. Hypertension. 2014;64:195–200.

**Keywords**: Diabetes, streptozotocin, blood pressure, pulse wave velocity

### P.033

### Pulse waveform analysis for monitoring of left ventricular function in patients with severely reduced ejection fraction

Stefan Orter[1,4], **Bernhard Hametner**[1], Siegfried Wassertheurer[1], Kathrin Danninger[2], Antonis Argyris[3], Athanase Protogerou[3], Eugenijus Kaniusas[4], Ronald Binder[2], Thomas Weber[2]

[1]Center for Health & Bioresources, AIT Austrian Institute of Technology, Vienna, Austria, [2]Cardiology Department, Klinikum Wels-Grieskirchen, Wels, Austria, [3]Cardiovascular Prevention and Research Unit, Laiko Hospital, Athens, Greece, [4]Institute of Biomedical Electronics, Vienna University of Technology, Vienna, Austria

**Background:** Impaired systolic function of the left ventricle leads to shortening of the left ventricular ejection time (LVET) and heart rate adjusted LVET (iLVET)[1]. The aim of this study is the investigation of the improvement in left ventricular function using radial pulse waveforms compared to improvement assessed by ejection fraction (EF).

**Methods**: 37 patients (7 females) with heart failure (HF) with reduced ejection fraction (HFrEF) were treated according to HF guidelines. EF and its changes under treatment were monitored with echocardiography (EPIQ, Philips, Simpson method with apical 4-chamber view), and LVET was monitored with tonometry (SphygmoCor, AtCor Medical, method based on numerical derivatives)[2]. Furthermore, LVET was adjusted for heart rate[3]. Visualization of differences between first and second visit was done by 4-quadrant plots (Fig. 1) and concordance rate was calculated.

**Results**: Patients mean age and body height were 54 years, and 174 cm respectively. Their average weight decreased from 89 to 88 kg. Measured basic parameters on first and second visit were HR (68BPM vs. 60BPM), SBP (128 mmHg vs. 128 mmHg), DBP (81 mmHg vs. 76 mmHg), EF (25% vs. 42%), LVET (0.266 s vs. 0.289 s), iLVET (0.380 vs. 0.389), percentage of patients with betablocker intake (76% vs. 97%), and percentage of patients with ACE-I/ARB/ARNI intake (92% vs. 97%). The mean timespan between first and second measurements was 100 days. A concordance rate of 0.84 for LVET and 0.65 for iLVET was observed.

**Conclusions**: Automatically measured LVET and iLVET from radial pressure waveforms is suitable for monitoring the improvement of EF with medical treatment in HFrEF.

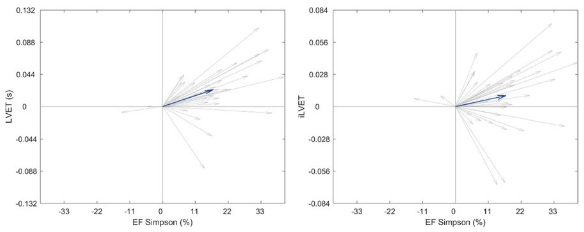

LVET (left) and iLVET (right) against EF Simpson, effect direction between second and first visit. Gray arrows show individual measurements, the blue arrow shows the mean effect over 37 subjects.

**References**

[1] A. Haiden, B. Eber, and T. Weber, 'U-Shaped Relationship of Left Ventricular Ejection Time Index and All-Cause Mortality', American Journal of Hypertension, vol. 27, no. 5, pp. 702–709, Mai 2014, https://doi.org/10.1093/ajh/hpt185.

[2] A. Bauer, B. Hametner, T. Weber, and S. Wassertheurer, 'Method Comparison and Validation of the Determination of Ejection Duration from Oscillometric Measurements', IFAC-PapersOnLine, vol. 51, no. 2, pp. 343–348, Jan. 2018, https://doi.org/10.1016/j.ifacol.2018.03.059.

[3] Lewis R P, Rittogers S E, Froester W F, and Boudoulas H, 'A critical review of the systolic time intervals', Circulation, vol. 56, no. 2, pp. 146–158, Aug. 1977, https://doi.org/10.1161/01.CIR.56.2.146.

### P.034

### Sex-specific association between the metabolic score for insulin resistance and arterial stiffness in middle-aged adults with metabolic syndrome

**Jurgita Mikolaitytė**[1], Agnė Laučytė-Cibulskienė[2,3], Ligita Ryliškytė[3], Jolita Badarienė[1,3]

[1]State Research Institute Centre for Innovative Medicine, Vilnius, Lithuania, [2]Department of Nephrology, Lund University, Malmö, Sweden, [3]Faculty of Medicine, Vilnius University, Vilnius, Lithuania

**Background:** The Metabolic Score for Insulin Resistance (METS-IR) is a non-insulin-based metabolic index used as a substitution marker of insulin resistance and cardiometabolic risk. Previous studies have suggested that insulin resistance is significantly related to the development and progression of coronary atherosclerosis and adverse plaque characteristics. The main objective of this study was to evaluate the sex-specific relationship between METS-IR and arterial parameters in the middle-aged Lithuanian population with metabolic syndrome.

**Methods**: A total of 2064 subjects (1136 women and 928 men). Anthropometric, laboratory testing, and cardiovascular risk factors along with arterial parameters (carotid-radial pulse wave velocity (crPWV), carotid-femoral pulse wave velocity (cfPWV), carotid intima-media thickness (CIMT), ankle-brachial index (ABI), cardio-ankle vascular index (CAVI) and atherosclerotic plaques) were evaluated.

**Results**: After stratifying subjects into sex-specific METS-IR quartiles, we observed statistically significant differences in all arterial parameters among METS-IR quartiles, except for crPWV in men ($p = 0.533$). Differences between men and women in the METS-IR quartiles were observed only in cfPWV ($p < 0.05$), CAVI ($p < 0.05$), and CIMT ($p < 0.001$). In a fully adjusted linear regression analysis, METS-IR was associated with CAVI in both men ($p = 0.005$) and women ($p < 0.001$). However, ABI—only in men ($p = 0.040$), and CIMT – in women ($p = 0.025$).

**Conclusion**: Insulin resistance measured by METS-IR is associated with CAVI in both men and women in the middle-aged Lithuanian population with metabolic syndrome. Additionally, in men, it is also associated with ABI, whereas in women – with CIMT.

**Keywords**: Arterial stiffness, cardio-ankle vascular index, carotid intima-media thickness, ankle-brachial index, Metabolic Score for Insulin Resistance

### P.036

### The effects of different types of calorie restriction on atherosclerosis-related miRNAs in mice

**Dilara Buse Durdabak**[1], Nazim Arda Keles[2], Soner Dogan[2], Bilge Guvenc Tuna[1]

[1]Department of Biophysics, School of Medicine, Yeditepe University, Istanbul, Turkey, [2]Department of Medical Biology, School of Medicine, Yeditepe University, Istanbul, Turkey

**Atherosclerosis** is a chronic inflammatory blood vessel disease.Studies highlight the importance of epigenetic modifications specifically miRNAs in the development and progression of atherosclerosis.Calorie restriction(CR) is one of the best-known interventions to prolong lifespan and impact lowering the risk of atherosclerosis.In the present study, the effects of different types of CR on atherosclerosis-related miRNA were studied.

Female mice were enrolled into three groups; ad-libitum (AL), Chronic-CR (CCR, 15% CR), and Intermittent-CR (ICR) which 60% CR was applied for one week (ICR-R, restricted) followed by three weeks of AL feeding (ICR-RF, refeed).Blood and brain samples were collected at week 49/50 to measure miRNA expression levels using Affymetrix GeneChip miRNA 4.1 Array.The targets of differentially expressed(DE) miRNAs that are enriched in atherosclerosis-related molecular pathways were analyzed.

In blood, a total of 12 miRNAs were DE among dietary groups.There were common miRNAs that differ in dietary regimes when compared to the AL group; miR-709(17,09-fold higher), miR-30b-5p(7,12-fold lower), and miR-19b-3p(5,72-fold lower) in CCR.The overexpression of miR-709 is shown to have a cardioprotective effect[1], while miR-30b-5p[2] and miR-19b-3p[3] are considered pro-atherosclerotic.GO-KEGG analyses revealed that targets of atherosclerosis-related miRNAs that were affected with CR were also enriched in aging and cancer-related molecular pathways.In the brain, a total of 6 miRNAs were differentially expressed.Interestingly, there was no significant change in atherosclerosis-related miRNAs between blood and brain.

In conclusion, even though CR has different effects on blood and brain tissues, some common miRNAs might have protective effects on atherosclerosis, suggesting the link between the brain and vascular axis.

**References**

1. Li M, Chen H, Chen L, Chen Y, Liu X, Mo D. miR-709 modulates LPS-induced inflammatory response through targeting GSK-3β. Int Immunopharmacol. 2016 Jul;36:333–8.
2. Qi X, Wang H, Xia L, Lin R, Li T, Guan C, et al. miR-30b-5p releases HMGB1 via UBE2D2/KAT2B/HMGB1 pathway to promote pro-inflammatory polarization and recruitment of macrophages. Atherosclerosis. 2021 May;324:38–45.
3. Wang J, Xu X, Li P, Zhang B, Zhang J. HDAC3 protects against atherosclerosis through inhibition of inflammation via the microRNA-19b/PPARγ/NF-κB axis. Atherosclerosis. 2021 Apr;323:1–12.

**Keywords**: Atherosclerosis, calorie restrictions, miRNA

### P.037

### Diabetes Mellitus is associated with relatively higher arterial stiffness when compared to hypertension or hyperlipidemia post-recovery from COVID-19

Rinkoo Dalan[1,2], Barnaby Young[2,3], David Lye[2,3], Bernhard Boehm.[2]

[1]Tan Tock Seng Hospital, Singapore, Singapore, [2]Lee Kong Chian School of Medicine, Nanyang Technological University, Singapore, Singapore, [3]National Centre for infectious diseases, Singapore, Singapore

**Background:** COVID-19, is associated with vascular dysfunction, arterial stiffness and worse outcomes in diabetes mellitus (DM) (1). We hypothesise that longer-term impact in COVID-19 DM patients will be worse when compared to other metabolic conditions, 1 year after recovery from COVID-19.

**Methods**: We recruited 63 patients belonging to the three groups: group 1 – recovered COVID-19 DM-type 2, n = 14; Group 2- recovered COVID-19, non-diabetes hypertension or hyperlipidemia, n = 29 and Group 3- non-COVID-19 DM, n = 14. Data collected: 1. Demographics, 2. Anthropometry, 3. Metabolic profile, 4. Reactive Hyperaemia index (LnRHI), Augmentation index (AI@75), Heart Rate variability (HRV) (RHI-EndoPAT), 5. Carotid-femoral Pulse-Wave-velocity (cfPWV), central pulse pressure (CPP) (Sphygmocor); 6. Autofluorescence- Advanced glycation end-products (AGE-Reader); 7. Carotid intima-media thickness (CIMT). We performed one-way ANOVA to compare differences in the three groups.

**Results**: Demographics and anthropometry (BMI and waist circumference) were comparable in all three groups. When compared to Groups 2 and 3, In Group 1: Troponin and the red cell distribution width (RDW) was higher ($p < 0.05$); Sphygmocor based cfPWV & CPP, was higher ($p < 0.001$); RHI-EndoPAT based AI (@75) was higher and HRV(SDNN) was lower ($p < 0.05$); AGE was higher ($p < 0.01$) (see Fig. 1).

**Conclusion**: Measures of arterial-stiffness (cf-PWV & AI@75) and HRV are more significantly impaired in DM when compared to hypertension/hyperlipidemia, one year post COVID-19 recovery. These measures are higher when compared to similar matched diabetes patients with no history of COVID-19 infection. COVID-19 DM patients need to be followed up to study long-term impact on vascular complications and autonomic neuropathy.

Table 1: Vascular Function Measurements in the 3 groups

| | Group 1 | Group 2 | Group 3 | One-Way ANOVA P -values |
|---|---|---|---|---|
| N=63 | 14 | 29 | 14 | |
| Troponin, | 4.6(2.8) | 2.6(1.1) | 3.6(3.1) | 0.0323* |
| RDW % | 15.0(1.9) | 13.7(0.9) | 13.5(0.5) | 0.0021* |
| CIMT mm, | 0.69(0.13) | 0.54(0.13) | 0.67(0.12) | 0.0007* |
| Ln-RHI | 0.69(0.25) | 0.71(0.27) | 0.73(0.31) | 0.9369 |
| AI (75/min) | 18.8(15.5) | 2.9(15.2) | 5.9(15.3) | 0.0086* |
| Heart Rate | 76.5(11.0) | 66.3(6.8) | 65.1(12.3) | 0.0025* |
| SDNN | 31.6(16.6) | 47.9(15.9) | 38.9(18.0) | 0.0120* |
| PWV (Sphygmocor) | 9.0(1.4) | 6.7(1.4) | 7.5(0.9) | 0.0000* |
| AGE reader | 3.8(1.4) | 3.1(0.7) | 2.6(0.5) | 0.0041* |

Mean (SD) values reported. *<0.05;

Table showing various vascular measures and figure showing box plot on the pulse-wave-velocity in the three groups

Figure 1: Box-Plot showing the pulse wave velocity in the three groups

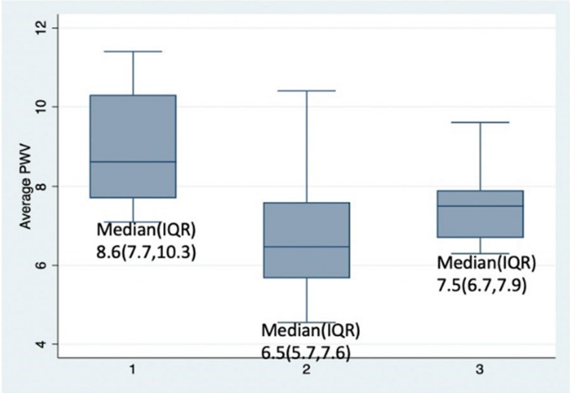

Group 1: Diabetes and COVID; Group 2: HTN/HLD with COVID;
Group 3: Diabetes with no COVID

**Reference**

1. Zota IM, Stătescu C, Sascău RA, et al. Acute and Long-Term Consequences of COVID-19 on Arterial Stiffness-A Narrative Review. Life (Basel). 2022;12(6):781.

**Keywords**: COVID-19; arterial-stiffness, pulse-wave-velocity, diabetes

### P.038

### Large artery stiffness using Sphgymocor technology shows higher augmentation index in pre-diabetes and diabetes in multi-ethnic Singapore

**Ying Jie Chee**[1], Rinkoo Dalan

[1]Tan Tock Seng Hospital, Singapore, Singapore

**Background:** The Sphygmocor technology uses applanation tonometry to measure large artery stiffness. Although the carotid-femoral pulse wave velocity (cfPWV) is higher in diabetes, an association with pre-diabetes has not been observed.(1,2) There is limited data on augmentation index (AIx). We aimed to study the correlation of arterial stiffness among healthy, pre-diabetes, or diabetes in multi-ethnic Singapore.

**Methods**: Population: n = 130; Age = 44.8 (9.6) years; Male = 41 (31%), Chinese = 93, Indians = 12, Malays = 15, Others = 10. All participants underwent a standard 75 g oral glucose tolerance test and applanation tonometry to assess cfPWV, central pulse pressure (CPP) and AIx. One-way ANOVA was done to study the differences in the arterial measurements based on diabetes status.

**Results**: Healthy (n = 81), Prediabetes (n = 27), Diabetes (n = 22). While cfPWV was higher in diabetes (mean (SD): 7.2(1.6)) compared to absence of diabetes (6.5(1.0)); p < 0.01), there was no difference between healthy 6.5(1.1) and pre-diabetes (6.4(1.0)); p > 0.01. An increasing trend was seen in AIx, healthy (mean (SD): 9.5(4.3)) < pre-diabetes (mean (SD): 11.1(5.4)) < diabetes (mean (SD):13.1(7.0)); p < 0.01. No statistically significant difference was seen in CPP (p > 0.01).

**Discussion**: AIx may reflect early markers of impaired glucose tolerance or pre-diabetes. Moreover, as AIx is determined by the properties of the distal vasculature, it may be used as an early marker of distal circulatory dysfunction involving the small arterioles, which precede abnormalities in pulse wave velocities. AIx can be a valuable marker of early vascular dysfunction, especially among individuals with pre-diabetes. Further studies are needed to understand the mechanistic basis of this trend.

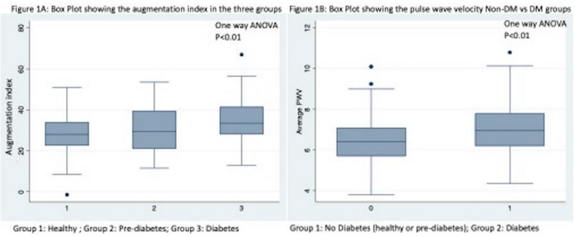

**Fig. 1** (A) augmentation index and (B) pulse wave velocity in healthy, pre-diabetes and diabetes

### References

1. Henry RM, Kostense PJ, Spijkerman AM, Dekker JM, Nijpels G, Heine RJ, et al. Arterial stiffness increases with deteriorating glucose tolerance status: the Hoorn Study. Circulation. 2003 Apr;107(16):2089–95. https://doi.org/10.1161/01.CIR.0000065222.34933.FC.
2. Prenner SB, Chirinos JA. Arterial stiffness in diabetes mellitus. Atherosclerosis. 2015 Feb;238(2):370–9. https://doi.org/10.1016/j.atherosclerosis.2014.12.023.

**Keywords:** Arterial stiffness, augmentation index, pre-diabetes, diabetes

### Clinical aspects

### P.042

### Estimating pulse wave velocity in Community Pharmacies improves CV-risk stratification compared to SCORE

**Enrique Rodilla**[1,2], Manuel Adell[3], Vicente Baixauli[3], Otón Bellver[3], Lidón Castillo[3], Santiago Centelles[3], Edelmira Córcoles[3], Rosario Hernández[3], Sara Martínez[3], Zeneida Perseguer[3], Rosa Prats[3], Javier Reig[3], Desiré Ruiz[3], Fanny Ruiz[3], Luis Salar[2,3], José-Antonio Costa[2], José Chordá[2], Julio Vicente[1], Ana Gómez[1], Maite Climent[3]

[1]Hospital Universitario de Sagunto, Puerto De Sagunto, Spain, [2]Universidad Cardenal Herrera-CEU, CEU Universities, Moncada (Valencia), Spain, [3]Sociedad Española de Farmacia Familiar y Comunitaria (SEFAC-Valencia), Valencia, Spain

**Background:** Arterial stiffness is considered to be an intermediate marker of CV risk with independent prognostic value. The objective of this study is to assess whether the estimation of arterial stiffness can improve CV risk stratification compared to SCORE in patients at Community Pharmacies.

**Methods**: Observational prospective epidemiological study in which consecutive individuals entering a participating Community Pharmacy are offered a voluntary measurement of blood pressure and estimation of pulse wave velocity by oscillometry (AGEDIO, IEM®) to stratify their CV risk according to SCORE compared to the use of arterial stiffness.

**Results**: After nine months of recruitment, data from 923 patients (173 women, 102 men) were collected. 16/122 (13.1%) patients under 40 years and 72/364 (19.8%) over 65 years of age presented pathological stiffness and could be classified as high-risk, even though being out of the age-range of SCORE. Of the 437 (47.3%) patients who were susceptible to calculating SCORE, 42/437 patients (9.6%) presented pathological arterial stiffness. Cholesterol values were available in 281 patients (64.3%). Among them, according to SCORE, only 6 (2.1%) fell into the high-risk category.

**Conclusions**: More than half of the subjects who randomly enter a community pharmacy had ages that make it impossible to calculate the CV risk by SCORE. Among them, arterial damage was detected in 18.1%. Of the other half, 9.6% presented arterial damage and, therefore, high CV risk, when SCORE only detected it in 2.1%. Therefore, estimating arterial stiffness in community pharmacies markedly improves detection of high CV risk compared to SCORE.

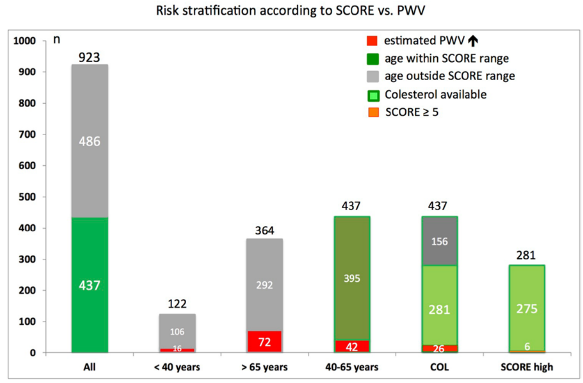

**References**
- Rodilla Sala E, Adell Alegre M, Giner Galvañ V, Perseguer Torregrosa Z, Pascual Izuel JM, Climent Catalá MT, et al. Arterial stiffness in normotensive and hypertensive subjects: frequency in community pharmacies. Med Clin (Barc) 2017;149:469–76 [Article in English, Spanish].
- Nunan D, Fleming S, Hametner B, Wassertheurer S. Performance of pulse wave velocity measured using a brachial cuff in a community setting. Blood Press Monit. 2014;19:315–9.

**Keywords**: Pulse wave velocity, brachial oscillometry, risk stratification, pharmacies

### P.043

### Evaluation of office and ambulatory central blood pressure by two methods and their changes after lifestyle or medical interventions in hypertension

**János Nemcsik**[1], Helga Gyöngyösi[2], Dóra Batta[1], Andrea László[3], Dr. Péter Torzsa[1], Beáta Kőrösi[1], Zsófia Nemcsik-Bencze[4], Orsolya Cseprekál[5], András Tislér[6]

[1]Semmelweis University, Department Of Family Medicine, Budapest, Hungary, [2]Semmelweis University, Faculty of Medicine, Budapest, Hungary, [3]Norisana- MVZ Rosenau, Nuremberg, Germany, [4]Semmelweis University, Department of Neuroradiology, Budapest, Hungary, [5]Semmelweis University, Department of Transplantation and Surgery, Budapest, Hungary, [6]Semmelweis University, Department of Internal Medicine and Oncology, Budapest, Hungary

**Background:** Central systolic blood pressure (cSBP) can be evaluated in office and also in ambulatory condition, during 24-h monitoring. The aim of our study was to measure office brachial systolic BP (bSBP) and cSBP in the office and brachial SBP and cSBP in 24-h setting.
**Methods**: Office cSBP was measured with PulsePen (PP cSBP), while 24-h ambulatory brachial SBP (24 h bSBP), and cSBP were evaluated with Mobil-O-Graph. For the calculation of 24-h cSBP both systolic/diastolic and systolic/mean BP calibration methods were considered (24 h cSBPC1 and 24 h cSBPC2, respectively). In new hypertensive patients (HT) the measurements were repeated 3 months after the initiation of antihypertensive medication. In white-coat hypertensive patients (WhHT) after lifestyle modifications the measurements were repeated at 12 months.
**Results**: 105 patients were involved with 22 HT and 22 WhHT subjects. bSBP (140.8 ± 17 mmHg) was higher than PP cSBP (128.2 ± 13.1 mmHg, $p < 0.05$). 24 h bSBP (128.3 ± 10.3 mmHg) was higher than 24 h cSBPC1 (117.8 ± 9.3 mmHg, $p < 0.05$), but equal with 24 h cSBPC2 (131.1 ± 11.1 mmHg). For medical intervention bSBP (Δ20.4 mmHg) and PP cSBP (Δ16 mmHg) decreased markedly, and 24 h bSBP (Δ10.9 mmHg), 24 h cSBPC1 (Δ10.1 mmHg) and 24 h cSBPC2 (Δ9 mmHg) decreased equally (all $p < 0.05$). For lifestyle changes only bSBP changed significantly (Δ6.2 mmHg).
**Conclusions**: These results suggest differences in absolute values of cSBP in office and 24 h with different calibrations, but similarities in the changes of the magnitude of cSBP in office and 24-h with bSBP in the similar settings.

**Keywords**: Central systolic blood pressure

### P.044

### The effects of RIPC on metabolome in patients undergoing vascular surgery: a randomized controlled trial

**Kadri Eerik**[1,2,3], Teele Kasepalu[1,2,3], Jaan Eha[1,2,3], Aigar Ottas[4], Jaak Kals[1,5,6]

[1]Endothelial Research Centre, University of Tartu, Tartu, Estonia, [2]Department of Cardiology, Institute of Clinical Medicine, University of Tartu, Tartu, Estonia, [3]Heart Clinic, Tartu University Hospital, Tartu, Estonia, [4]Department of Biochemistry, Institute of Biomedicine and Translational Medicine, Centre of Excellence for Genomics and Translational Medicine, University of Tartu, Tartu, Estonia, [5]Department of Surgery, Institute of Clinical Medicine, University of Tartu, Tartu, Estonia, [6]Surgery Clinic, Tartu University Hospital, Tartu, Estonia

**Background:** Remote ischemic preconditioning (RIPC) is a phenomenon in which short episodes of ischemia are applied to distant organs to prepare target organs for more prolonged ischemia and induce protection against ischemia–reperfusion injury [1]. The aim of this study was to evaluate whether preoperatively performed RIPC affects metabolome following vascular surgery and assess if metabolomic changes correlate with heart and kidney injury markers.
**Methods**: A randomized-controlled, double-blinded trial was conducted in the Tartu University Hospital. Patients undergoing open surgical repair of abdominal aortic aneurysm, surgical lower limb revascularization, and carotid endarterectomy were recruited. A RIPC consisting of four cycles of 5 min of ischemia followed by 5 min of reperfusion was applied before the operation. The blood was collected preoperatively and approximately 24 h postoperatively. The metabolome was analyzed with the AbsoluteIDQ p180 Kit.
**Results**: The final analysis included 45 patients from the RIPC and 47 from the sham group. Baseline characteristics and values of metabolites were statistically similar between groups. RIPC did not cause statistically significant changes in metabolites 24 h postoperatively. There was a significant positive correlation between the change Kynurenine/Tryptophan ratio and the changes of hs-Troponin T ($r = 0.570$, $p < 0.001$), NT-proBNP ($r = 0.552$, $p < 0.001$), Cystatin C ($r = 0.534$, $p < 0.001$) and Beta-2-Microglobulin ($r = 0.504$, $p < 0.001$).
**Conclusions**: Preoperatively performed RIPC did not significantly affect metabolome 24 h after vascular surgery. The positive linear correlation between Kynurenine/Tryptophan ratio and heart and kidney injury markers suggests that the Kynurenine-Tryptophan pathway can play a role in RIPC-associated cardio- and nephroprotective effects.

**Reference**
1. Anttila V, Haapanen H, Yannopoulos F, Herajärvi J, Anttila T, Juvonen T. Review of remote ischemic preconditioning: from laboratory studies to clinical trials. Scand Cardiovasc J. 2016 Oct-Dec;50(5–6):355–361.

**Keywords**: Metabolomics, remote ischemic preconditioning, vascular surgery

### P.045

### Repeated SBP Measurements during a Single Visit and Cardiovascular Prediction: Analysis of CARTaGENE

**Louis-Charles Desbiens**[1,2], Annie-Claire Nadeau-Fredette[1,2], François Madore[2,3], Mohsen Agharazii[4,5], Rémi Goupil[2,3]

[1]Hopital Maisonneuve-Rosemont, Montreal, Canada, [2]Université de Montréal, Montréal, Canada, [3]Hôpital du Sacré-Coeur de Montréal, Montréal, Canada, [4]CHU de Québec, Québec, Canada, [5]Université Laval, Québec, Canada

**Background:** Blood pressure (BP) has high intra-individual variability. Several guidelines recommend averaging BP measurements to monitor hypertension as it correlates more closely with ambulatory BP. However, whether these averages improve cardiovascular prediction has never been evaluated yet.
**Methods**: We studied individuals aged between 40 and 69 from the CARTaGENE cohort (Canada). Three SBP measurements ($SBP_1$, $SBP_2$, $SBP_3$) at two-minute intervals were taken with an Omron 907L device. These values were averaged to generate $SBP_{12}$ (mean of $SBP_1$ and $SBP_2$), $SBP_{23}$ ($SBP_2$ and $SBP_3$), and $SBP_{123}$ ($SBP_1$, $SBP_2$ and $SBP_3$). Major adverse atherosclerotic events (MACE: cardiovascular death, stroke, myocardial infarction) during a 10-year follow-up were obtained using medico-administrative databases. Associations of SBP parameters with MACE were obtained using fully adjusted Cox models. Predictive performance was assessed with 10-year atherosclerotic cardiovascular disease scores (ASCVD; using pooled cohort equations) for each SBP parameter and associated C-statistics.

**Results**: From 17,966 individuals, 2,378 had a MACE during the follow-up. SBP values at baseline were 126.5 mmHg ($SBP_1$), 123.2 ($SBP_2$) and 122.5 ($SBP_2$). After adjustment, $SBP_3$ had the strongest association with MACE. This association was significantly greater than that observed for $SBP_1$, $SBP_{12}$, or $SBP_{123}$. In comparison to $SBP_1$, $SBP_2$ and $SBP_3$ increased the risk attributable to SBP by up to two times. When included in ASCVD scores, $SBP_3$ yielded the highest C-statistic, which was significantly higher than all other SBP parameters except $SBP_{23}$.
**Conclusion**: Averaging SBP measurements during a single visit improves cardiovascular prediction compared to a single measurement. Discarding the first SBP value maximises predictive performance.

Table

| Parameter | Hazard ratio | | C-Statistic | | Attributable risk ratio (compared to $SBP_1$) | |
|---|---|---|---|---|---|---|
| | Fully adjusted value (95% CI) | Comparison with $SBP_3$ (*p*-value) | Value | Difference with $SBP_3$ (95% CI) | Men | Women |
| **Crude values** | | | | | | |
| $SBP_1$ | 1.06 (1.01, 1.10) | 0.042 | 67.56 | -0.35 (-0.19, -0.52) | Ref | Ref |
| $SBP_2$ | 1.08 (1.03, 1.12) | 0.065 | 67.75 | -0.17 (-0.30, -0.03) | 1.74 | 1.29 |
| $SBP_3$ | 1.10 (1.05, 1.15) | Ref | 67.92 | Ref | 2.06 | 1.82 |
| **Mean values** | | | | | | |
| $SBP_{1-2}$ | 1.07 (1.03, 1.12) | 0.033 | 67.69 | -0.23 (-0.36, -0.10) | 1.43 | 1.21 |
| $SBP_{2-3}$ | 1.09 (1.05, 1.14) | 0.064 | 67.86 | -0.05 (-0.12, 0.02) | 2.01 | 1.63 |
| $SBP_{1-2-3}$ | 1.08 (1.04, 1.13) | 0.025 | 67.79 | -0.13 (-0.22, -0.04) | 1.71 | 1.46 |

*Hazard ratios (95th confidence interval*) are displayed for one standard deviation increase. P-values for the comparison with the $SBP_3$ hazard ratio were computed using non-nested likelihood ratio tests.
Fully adjusted models include age, sex, self-reported race, BMI, active smoking, total cholesterol, HDL cholesterol, eGFR, statin use, antihypertensive use, prior cardiovascular disease, and mean heart rate.
*C-Statistics* were computed using predicted 10-year MACE risk from the ASCVD score (revised pooled cohort equations). Each C-Statistic was compared to the maximal one ($SBP_3$) to generate *C-Statistic differences (95% confidence interval)*.
*Attributable risk ratios* were obtained by dividing the excess risk for a given SBP value (defined as the difference between the predicted risk at the SBP value minus the predicted risk at a reference SBP of 120 mmHg) by the excess risk at the corresponding SBP value for $SBP_1$ and averaging these ratios over all SBP values. Fully adjusted Cox models were used to generate predicted risks.

**Keywords**: Hypertension Prediction Monitoring

### P.046

### Flow-mediated vasodilation and endothelial function in Mexican patients with type 2 diabetes mellitus, a cross-sectional study in western Mexico

**Luis Ricardo Balleza Alejandri**[1,2,3], Fernando Grover Páez[1,2,3], Erick González Campos[1,2,3], Carlos Gerardo Becerra Ramos[1,2,3], German Cardona Gutierrez[1,2,3], Sara Pascoe Gonzalez[1,2,3], Claudia Yanette Galan Ruiz[1,2,3], Javier Esparza Pimentel[1], David Cardona Müller[1,2,3]

[1]Universidad de Guadalajara, Guadalajara, Mexico, [2]Departamento de Fisiología, Guadalajara, México, [3]Instituto de investigación clínica y terpeútica (INTEC), Guadalajara, México

**Flow-mediated** vasodilation and endothelial function in Mexican patients with type 2 diabetes mellitus, a cross-sectional study in western Mexico.

**Introduction**: Mexico is a country with high mortality due to diabetes complications (1); constant hyperglycemia in patients with diabetes leads to endothelial damage, which is the main risk factor for the development of macro and microvascular complications, leading to an increased risk of mortality (2). Flow-mediated vasodilation (FMD) is one of the most widely used techniques for the evaluation of endothelial function and can be used as predictor of cardiovascular risk (3,4).
**Objective**: The objective of this study is to determine the FMD values and hemodynamic characteristics in Mexican patients with type 2 diabetes mellitus in western Mexico.
**Methods**: FMD were measured with a high-resolution semi-automatic ultrasound UNEX-EF 38G (UNEX Co. Ltd Nagoya Japan). Measurement of arterial tension were made with an OMRON electronic digital sphygmomanometer (HEM 907 XL).
**Results**: 65 patients, (28 men and 37 women) with a mean age of $52.46 \pm 11.71$, we found a difference between the basal and final diameters and blood flow between men and women ($4.67 \pm 0.76$ vs $3.42 \pm 0.69$ p 0.001; $4.96 \pm 0.76$ vs $3.66 \pm 0.70$ p 0.001; ($10.5 \pm 8.05$ vs $4.59 \pm 3.56$ p 0.020); an inverse correlation was found between FMD and SBP ($r = -0.265$ p 0.33).
**Conclusion**: We found a lower FMD in women than in men, it is also noteworthy the differences found in the flow and bIMT between the individuals in the study, the above can be explained by the inherent hormonal effects of each sex.

**References**
1. Romero-Martínez M, Shamah-Levy T, Vielma-Orozco E, Heredia-Hernández O, Mojica-Cuevas J, et al. Encuesta Nacional de Salud y Nutrición 2018–19:Resultados nacionales. salud publica mex.2020 Jan 13;61(6).
2. Zoungas S, Arima H, Gerstein HC, Holman RR, Woodward M et al. Effects of intensive glucose control on microvascular outcomes in patients with type 2 diabetes: a meta-analysis of individual participant data from randomised controlled trials. The Lancet Diabetes and Endocrinology.2017;5(6):431–7.
3. Korkmaz H, Onalan O. Evaluation of endothelial dysfunction: Flow-mediated dilation. Endothelium: Journal of Endothelial Cell Research. 2008;15(4):157–63.
4. Poredos P, Jezovnik MK. Testing Endothelial Function and its Clinical Relevance. Journal of Atherosclerosis and Thrombosis. 2012;20(1):1–8.

**Keywords**: FMD, Type 2 Diabetes, Endothelial function

### Epidemiology

### P.051

### The association of measures of hyperglycemia and the different frequency domains of microvascular flowmotion: the Maastricht Study

**Xiaofei Zhao**[1], Casper Schalkwijk[1], Bram Kroon[1], Coen Stehouwer[1], Dr. Boy Houben.[1]

[1]CARIM School for Cardiovascular Disease, Department of Internal Medicine, Maastricht University Medical Center +, Maastricht, Netherlands

**Introduction:** MVD may develop early and contribute to impaired insulin-mediated glucose uptake and subsequent metabolic insulin resistance, characterized by hyperglycemia. However, apart from hyperglycemia being a consequence of MVD, hyperglycemia can also (further) impair microvascular function, constituting a vicious cycle. Our aim was to study whether measures of hyperglycemia are associated with different components of skin microvascular flowmotion (SMF).
**Methods**: SMF was measured using laser-Doppler flowmetry (LDF). The relative contribution (percentage of the total) of the SMF components was used as outcome measures. We investigate the associations of measures of hyperglycemia (Fasting plasma glucose[FPG], 2-h plasma glucose[2-h PG], HbA1c, advanced glycation end-products[AGEs] assessed as skin autofluorescence [SAF]), and indices of glucose variability (incremental glucose peak [IGP] and continuous glucose monitoring [CGM] -assessed as standard deviation [SD]) with total SMF and the relative contribution of five different components.
**Results**: Greater FPG, 2-h PG, and HbA1c were statistically significant associated with lower Endothelial Power component(%) (per SD, respectively $-0.035$ [$-0.066$; $-0.004$]; $-0.047$ [$-0.079$; $-0.015$]; and $-0.030$ [$-0.061$; $-0.001$]). Greater FPG, and 2-h PG were statistically significant associated with higher Neurogenic Power component(%) (per SD, respectively 0.031 [0.000; 0.062]; and 0.048 [0.016; 0.079]). Greater FPG, 2-h PG, and HbA1c were statistically significant associated with higher Myogenic Power component(%) (per SD, respectively 0.039 [0.008; 0.071]; 0.040 [0.008; 0.072]; and 0.039 [0.008; 0.070]).
**Conclusion**: Higher levels of hyperglycemia and daily glucose variability were associated with a lower relative contribution of endothelial skin flowmotion component and, a higher relative contribution of neurogenic and myogenic components.

**References**
Houben, A.J.H.M. and C.D.A. Stehouwer, Microvascular dysfunction: Determinants and treatment, with a focus on hyperglycemia. Endocrine and Metabolic Science, 2021. 2.
Stefanovska, A., M. Bracic, and H.D. Kvernmo, Wavelet analysis of oscillations in the peripheral blood circulation measured by laser Doppler technique. IEEE Transactions on Biomedical Engineering, 1999. 46(10): p. 1230–1239.

**Keywords**: Microvascular dysfunction (MVD), Skin microvascular flowmotion (SMF), Type 2 diabetes (T2D)

### P.053

### Agreement between cuff device versus radial tonometry to measure central blood pressure and pulse wave analysis in adolescents

**Alun Hughes**[1]

[1]UCL, London, United Kingdom

**Background:** Cuff-based devices are increasingly used to measure central blood pressure (BP) and perform pulse wave analysis. Cuff devices have been compared to arterial tonometry in adults, [1] but information in adolescents is limited. Brachial amplification is large in young people and may influence agreement between methods.
**Methods**: Participants were recruited from the Avon Longitudinal Study of Parents and Children (ALSPAC) (http://www.alspac.bris.ac.uk). Ethical approval was obtained from the ALSPAC Law and Ethics Committee and the Local Research Ethics Committee. Participants provided written informed consent. 136 participants (age 17.6y (SD = 0.4); 68 (50%) male) had measurements of brachial suprasystolic waveforms (Pulsecor; BP +) and radial artery tonometry (Sphygmocor; SP) at the same clinic visit. Waveforms were calibrated to sitting BP according to manufacturer recommendations. Agreement was tested using Bland–Altman analysis and summarised as the mean differences with limits of agreement. Coefficient of variation (CV) and Pearson's correlation coefficient (r) were also calculated.
**Results**: Central systolic BP (cSBP) and maximum dp/dt were on average higher for BP +, but BP + and SP were closely correlated. Peripheral augmentation index (pAI) was on average lower for BP + than SP, and there was no bias for ejection duration (ED), while correlations were moderate for both. CV was ≤ 10% for all parameters except pAI (Table 1).
**Conclusions**: The two devices showed acceptable agreement. Differences in some parameters may reflect the influence of calibration, waveform morphology, or the use of different algorithms by devices.

**Table 1. Method comparison (Sphygmocor - Pulsecor)**

| Variable | Mean difference | Lower limit of agreement | Upper limit of agreement | CV, % | r |
|---|---|---|---|---|---|
| cSBP, mmHg | -5.4 | -16.2 | 5.3 | 5 | 0.84 |
| pAI, % | 16.0 | -13.0 | 45.0 | 27 | 0.54 |
| Maximum dp/dt, mmHg/s | -92.7 | -334.8 | 149.4 | 10 | 0.78 |
| Heart rate, bpm | 4.6 | -6.1 | 15.4 | 1 | 0.83 |
| Ejection_duration, s | 0.00 | -0.10 | 0.00 | 6 | 0.47 |

Abbreviations defined in text.

#### Reference

Park CM, Korolkova O, Davies JE, Parker KH, Siggers JH, March K, et al. Arterial pressure: agreement between a brachial cuff-based device and radial tonometry. J Hypertens. 2014;32(4):865–72.

**Keywords**: Central blood pressure, pulse wave analysis, method comparison.

### P.054

### Reproducibility of pulse wave analysis in adolescents.

**Alun Hughes**[1], Alicja Rapala[1], Nish Chaturvedi.[1]

[1]UCL, London, United Kingdom

**Background:** Previous studies have examined the reproducibility of central blood pressure (BP) and pulse wave analysis (PWA) in adults, but information in adolescents is limited. This is particularly relevant since central to brachial amplification is large in young people [1].
**Methods**: Participants were recruited from the Avon Longitudinal Study of Parents and Children (ALSPAC) (http://www.alspac.bris.ac.uk). Ethical approval was obtained from the ALSPAC Law and Ethics Committee and the Local Research Ethics Committee. Participants provided written informed consent. 14 participants (age 17.8y (SD = 0.2); 7 male) underwent 2 repeated measurements of sitting BP using an Omron 705-IT and radial artery tonometry (Sphygmocor). Radial waveforms were calibrated to systolic and diastolic pressure according to manufacturer recommendations. Bland–Altman analysis was performed, repeatability was assessed as the mean difference with limits of agreement, and Lin's concordance coefficient (CCC) was calculated as a measure of reliability.
**Results**: Reproducibility results are shown in Table 1.
**Conclusions**: Most parameters showed acceptable reliability, although AIx and T1 were poorly reproducible, possibly due to the large central to brachial amplification.

**Table 1** Reproducibility of pulse wave analysis measures.

| Variable | Mean difference | Lower limit of agreement | Upper limit of agreement | CCC |
|---|---|---|---|---|
| bSBP, mmHg | -2.4 | -13.0 | 8.1 | 0.84 |
| bDBP, mmHg | -1.4 | -13.2 | 10.4 | 0.66 |
| HR, bpm | 4.4 | -15.9 | 24.6 | 0.70 |
| cSBP, mmHg | -2.9 | -16.2 | 10.4 | 0.53 |
| cPP, mmHg | -1.6 | -10.5 | 7.3 | 0.78 |
| AIx, % | -3.4 | -41.2 | 34.3 | 0.09 |
| T1, ms | -5.1 | -52.9 | 42.7 | 0.16 |
| P1, mmHg | -2.1 | -10.5 | 6.2 | 0.81 |
| Buckberg index, % | -17.7 | -71.9 | 36.4 | 0.64 |
| Ejection_duration, ms | -1.2 | -36.0 | 33.6 | 0.42 |
| Duration_diastole, ms | -54.3 | -291.9 | 183.3 | 0.63 |
| SBP amplification, mmHg | 0.5 | -9.7 | 10.7 | 0.54 |

Abbreviations: bSBP, brachial systolic BP; bDBP, brachial diastolic BP; HR, heart rate; cSBP, central BP; cPP, central pulse pressure; AIx, augmentation index; T1, time of first shoulder; P1, pressure at first shoulder.

#### Reference

1. Ferreira DL, Fraser A, Howe LD, Jones S, Smith GD, Lawlor DA, et al. Associations of Central and Peripheral Blood Pressure With Cardiac Structure and Function in an Adolescent Birth Cohort: The Avon Longitudinal Study of Parents and Children. J Am Coll Cardiol. 2015;65(18):2048–50.

**Keywords**: Blood pressure, Pulse wave analysis, Adolescence, Reproducibility

### Interventions

### P.055

### The role of RIPC in preventing organ damage, inflammation and oxidative stress during lower limb DSA: a randomised controlled trial.

**Karl Kuusik**[1,2,3], Teele Kasepalu[1,2,3], Mihkel Zilmer[3], Jaan Eha[1,2], Mare Vähi[4], Liisi Anette Torop[5], Jüri Lieberg[6], Jaak Kals[3,6,7]

[1]Department of Cardiology, Institute of Clinical Medicine, University of Tartu, Tartu, Estonia, [2]Heart Clinic, Tartu University Hospital, Tartu, Estonia, [3]Department of Biochemistry, Institute of Biomedicine and Translational Medicine, Centre of Excellence for Genomics and Translational Medicine,University of Tartu, Tartu, Estonia, [4]Institute of Mathematics and Statistics, University of Tartu, Tartu, Estonia, [5]Pathology Service, Tartu University Hospital, Tartu, Estonia, [6]Department of Vascular Surgery, Surgery Clinic, Tartu University Hospital, Tartu, Estonia, [7]Department of Surgery, Institute of Clinical Medicine, University of Tartu, Tartu, Estonia

**Background:** Digital subtraction angiography (DSA) and percutaneous transluminal angioplasty (PTA) are common procedures for diagnosing and treating symptomatic lower extremity arterial disease (LEAD)[1]. However, organ damage following DSA and PTA is often under-recognised and hence undiagnosed[2,3]. To reduce the risk induced by invasive procedures in symptomatic LEAD patients, the method of remote ischemic preconditioning (RIPC) has been suggested[4,5]. The aim of the current study was to assess the effect of RIPC intervention on the organ damage markers profile, oxidative stress and inflammation biomarkers in LEAD patients undergoing DSA and PTA procedure.

**Methods**: The RIPC intervention was performed by inflating a standard blood pressure cuff on the patient's upper arm to 200 mmHg for 5 min four times with 5-min perfusion between each cycle. The sham intervention was performed similarly, but the cuff was inflated to 20 mmHg. Changes in the cardiac and renal damage biomarkers' profile, oxidative stress and inflammation biomarkers were recorded before and 24 h after DSA or DSA-PTA.

**Results**: RIPC significantly limited the increase of adiponectine levels after DSA/PTA procedure, compared to sham intervention ($p = 0.020$), but CK-MB levels were markedly lower in the sham group ($p = 0.047$) after DSA procedure. There was no significant difference between the RIPC and the sham group in mean changes in hs-Troponin-T ($p = 0.25$), NT-proBNP ($p = 0.24$), creatinine ($p = 0.76$), eGFR ($p = 0.61$), urea ($p = 0.95$), beta-2-microglobuline ($p = 0.34$) or cystatine C ($p = 0.24$) levels.

**Conclusions**: In this controlled clinical study RIPC failed to improve the profile of renal and cardiac biomarkers in patients with LEAD periprocedurally.

**References**

1—Gerhard-Herman MD et al. 2016 AHA/ACC Guideline on the Management of Patients With Lower Extremity Peripheral Artery Disease. Circulation 2017;135(12):e726–79.
2—Schillinger M et al. Balloon angioplasty and stent implantation induce a vascular inflammatory reaction. J Endovasc Ther. 2002;9(1):59–66.
3—Geenen RWF et al. Contrast-induced nephropathy: pharmacology, pathophysiology and prevention. Insights Imaging. 2013;4(6):811–20.
4—Totzeck M et al. Concepts of hypoxic NO signaling in remote ischemic preconditioning. World J Cardiol. 2015;7(10):645–51.
5—Yang J et al. Peripheral Mechanisms of Remote Ischemic Conditioning. Cond Med. 2019;2(2):61–8.l

**Keywords**: Remote Ischaemic Preconditioning. Lower extremity arterial disease. Digital Subtraction Angiography

### Kidney

### P.057

### Dapagliflozin does not influence arterial stiffness or other bio-markers of arterial ageing in people with type 2 diabetes and kidney disease

**Nikolaos Fountoulakis**[1], Dimitra Stathi[1], Maria Flaquer[1], Antonella Corcillo[1], Angeliki Panagiotou[1], Anastasios Mangelis[1], Salma Ayis[1], Luigi Gnudi[1], Janaka Karalliedde.[1]

[1]King's College London, London, United Kingdom

**Background:** Sodium glucose co-transporter 2 (SGLT-2) inhibitors have demonstrated renal benefits in people with type 2 diabetes (T2DM)[1]. Arterial stiffness as measured by aortic pulse wave velocity (Ao-PWV) is an index of arterial ageing and predicts cardio-renal outcomes[2]. The effect of SGLT-2 inhibitors on Ao-PWV and other markers of arterial ageing is unknown.

**Methods**: We performed a 24-week single center randomized controlled trial comparing dapagliflozin and ramipril (D + R) versus ramipril (R) on markers of arterial ageing in people with T2DM with residual albuminuria despite maximum tolerated renin angiotensin system (RAS) inhibition. Primary endpoint was change in urine albumin excretion rate (AER).Secondary endpoints included Ao-PWV (by applanation tonometry),central aortic blood pressure, mediators of the RAS (plasma renin activity,aldosterone,ACE-2 and angiotensin 1–7/1–9 levels) and biomarkers of arterial ageing [soluble Klotho (sKlotho) and fibroblast growth factor 23 (FGF-23)].

**Results**: 33 participants (male 72.7%) were randomized to Dapagliflozin and Ramipril ($n = 17$) or Ramipril ($n = 16$). After 24 weeks of treatment AER fell significantly [mean (95% CI)] only in D + R by 43.5% ($-57.4$ to $-29.6\%$) ($p < 0.01$) as compared to 5% ($-48.3$ to 38.3%) ($p = 0.36$) in R. Ao-PWV did not change significantly from baseline (D + R $9.06 \pm 1.91$ m/s to $9.13 \pm 2.03$ m/s vs R $9.88 \pm 2.12$ m/s to $10.0 \pm 1.84$ m/s).No significant changes were noted in central aortic blood pressure, augmentation index, sKlotho or FGF-23.

**Conclusion**: The combination of Dapagliflozin and Ramipril for 24 weeks significantly reduces albuminuria but does not impact on Ao-PWV or other mediators of arterial ageing in people with T2DM.

**References**

1. C. Wanner et al., Empagliflozin and Progression of Kidney disease in Type 2 Diabetes, N. Eng. J. Med. 2016.
2. C. Vlachopoulos et al., Prediction of cardiovascular events and all-cause mortality with arterial stiffness: a systematic review and meta-analysis, J Am Coll Cardiol. 2010.

**Keywords**: Arterial ageing;SGLT-2is;Diabetic kidney disease

### P.058

### Preliminary findings of the VALIDATion of central blood pressure Estimation in advanced Chronic Kidney Disease study (VALIDATE-CKD).

**Nadège Côté**[1,2], Yasmine Abbaoui[1], Théo Spanneut[1], Mohsen Agharazii[2], Rémi Goupil[1]

[1]Hôpital du Sacré-Cœur de Montréal, Université de Montréal, Montréal, Canada, [2]CHU de Québec Research Center, L'Hôtel-Dieu de Québec Hospital, Université Laval, Québec, Canada

**Background:** Brachial cuff blood pressure (BP) is used as a convenient surrogate to aortic BP, the true determinant of blood perfusion to central organs. In the general population, brachial cuff systolic BP (SBP) has an acceptable accuracy towards aortic BP but less is known in populations with high aortic stiffness, such as in advanced CKD. Central BP devices were designed to directly estimate the aortic BP through pulse wave analysis to a greater accuracy than brachial cuff BP, but these were never validated in the advanced CKD population. The aim of the ongoing VALIDATE-CKD study is to compare the accuracy of brachial and central BP readings towards the intraarterial aortic SBP, in patients with and without advanced CKD (eGFR < 30 ml/min/1.73 $m^2$ and dialysis).

**Methods**: In patients with and without CKD stage G4-G5 undergoing non-urgent coronary angiograms, invasive aortic and non-invasive (WatchBP and Mobil-o-graph devices) BPs were measured simultaneously in accordance to the ARTERY Society protocol. Accuracy was defined by the mean difference (± SD) between the aortic SBP and the simultaneously measured non-invasive SBP.

**Results**: To date, we enrolled 18 individuals with advanced CKD and 69 control subjects, with an aim to enroll 85 subjects in each group.

**Conclusions**: These early preliminary results suggest that brachial cuff SBP significantly underestimates aortic SBP in patients with advanced CKD. Furthermore, central BP devices may provide a better accuracy in this population. The ongoing VALIDATE-CKD study could support the use of central BP devices to enhance BP management in advanced CKD.

| | Advanced CKD (n=18) | Control (n=69) |
|---|---|---|
| Age (years) | 71 ± 11 | 67 ± 11 |
| Female sex (%) | 28 | 32 |
| eGFR range (ml/min/1.73 m²) | <10 to 29 | 33 to 106 |
| BMI | 26 ± 4 | 27 ± 5 |
| Brachial cuff SBP (mmHg) | 141 ± 27 | 140 ± 24 |
| Invasive aortic SBP (mmHg) | 146 ± 34 | 140 ± 26 |
| Pulse wave velocity with pOpmètre (m/s) | 11.5 ± 10.6 | 8.8 ± 5.5 |
| **Accuracy** | | |
| Brachial Cuff SBP (mmHg) | -5.2 ± 14.7 | -0.1 ± 13.5 |
| Mobil-o-graph Central SBP (mmHg) | 4.0 ± 13.8 | 5.6 ± 16.3 |
| WatchBP SBP (mmHg) | 4.4 ± 12.1 | 7.6 ± 15.9 |
| pOpmètre (mmHg) | -12.0 ± 12.3 | -10.9 ± 14.8 |

Minimal acceptable accuracy recommended by the ARTERY society is 5 ± 8 mmHg.

**Table 1** Clinical characteristics and SBPs accuracies in patients with and without advanced CKD

**Keywords**: Central blood pressure, brachial blood pressure, chronic kidney disease

### Models and methodologies

### P.061

### A clinically applicable model of active arterial mechanics accounting for the length dependency of smooth muscle cell contraction

Cindy Van Loo[1], Ryan Pewowaruk[2,3], Alessandro Giudici[1,4], Koen Reesink[1], Leon Schurgers[5], Tammo Delhaas[1], Adam Gepner[2,3], Bart Spronck[1,6]

[1]Dept. of Biomedical Engineering, CARIM School for Cardiovascular Diseases, Maastricht University, Maastricht, The Netherlands, [2]William S. Middleton Memorial Veterans Hospital, Madison, USA, [3]Dept. of Medicine—Division of Cardiovascular Medicine, University of Wisconsin School of Medicine and Public Health, Madison, USA, [4]GROW School for Oncology and Reproduction, Maastricht University, Maastricht, The Netherlands, [5]Dept. of Biochemistry, CARIM School for Cardiovascular Diseases, Maastricht University, Maastricht, The Netherlands, [6]Macquarie Medical School, Faculty of Medicine, Health and Human Sciences, Macquarie University, Sydney, Australia

**Background:** The mechanical role of contraction of vascular smooth muscle cells (VSMCs) in large arteries is often overlooked. Recently, Pewowaruk and Gepner proposed a clinically applicable mathematical model separating the passive and active contributions to arterial mechanics [1]. Subsequently, they applied the model to<i>in vivo</i>data from human carotid arteries (<i>n</i> =40) at baseline and after nitroglycerin-mediated vasodilation (Fig. A) [2]. In two participants, the VSMCs' active contribution decreased with increasing pressure from diastolic to systolic, which could not be captured with the original model (Fig. B). VSMC tension, generated by actin-myosin interaction, is maximal at the length with optimal filament overlap and is lower at other lengths. We hypothesised that, in these two participants, VSMCs operated beyond their optimal length. Accordingly, we modelled active VSMC tension as a function of length using a Gaussian-shaped function [3].

**Methods**: The baseline tension–diameter data for the two participants were fitted using the combined exponential (passive) and Gaussian (active) expression in Fig. G [3].

**Results**: In participant 1, VSMC contribution to tension was nearly diameter-independent, indicating VSMC was near its maximum contraction length (Fig. C–D). In participant 2, the VSMC contribution showed a strong negative relation with diameter (Fig. E–F), indicating VSMCs were beyond their maximum contraction length.

**Conclusions**: Our proposed Gaussian function enables capturing VSMC active tension behaviour in patients with VSMCs operating beyond their optimal length, based on pressure-diameter data. We speculate that in such case, actin-myosin unit rearrangement may be impaired [4], as typically VSMCs operate below their optimal length.

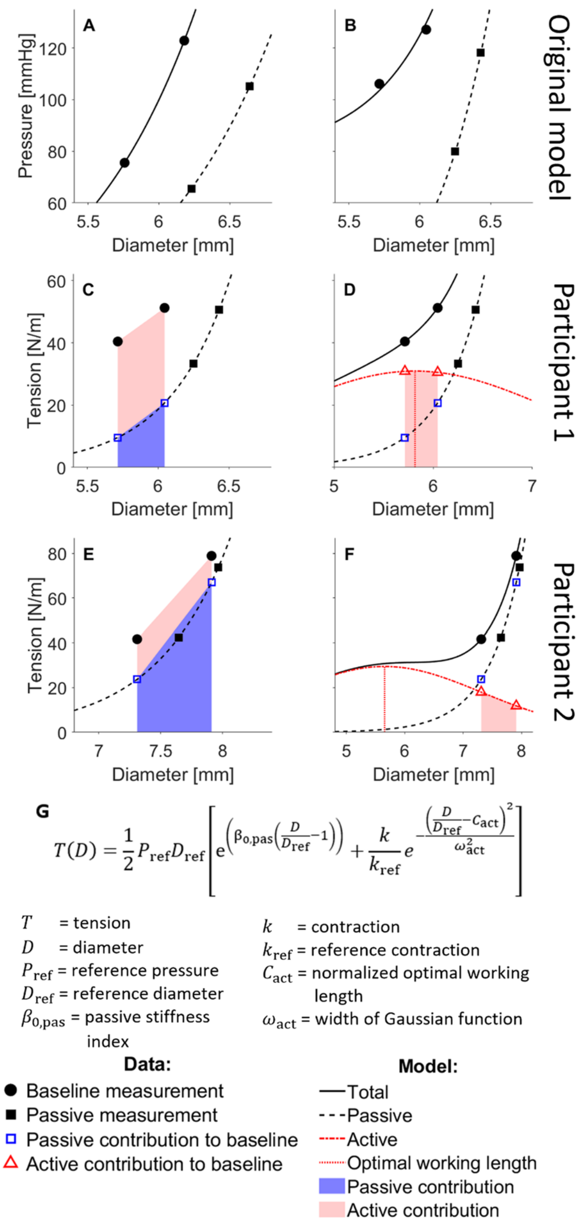

$$T(D) = \frac{1}{2} P_{\mathrm{ref}} D_{\mathrm{ref}} \left[ e^{\left(\beta_{0,\mathrm{pas}}\left(\frac{D}{D_{\mathrm{ref}}}-1\right)\right)} + \frac{k}{k_{\mathrm{ref}}} e^{-\frac{\left(\frac{D}{D_{\mathrm{ref}}}-C_{\mathrm{act}}\right)^2}{\omega_{\mathrm{act}}^2}} \right]$$

Example of original model fit without (<b>A</b>) and with (<b>B</b>) residual errors;<b>C</b>,<b>E</b>: Passive and active contributions to baseline data;<b>D</b>,<b>F</b>: Total and contraction model curves;<b>G</b>: Proposed model equation.

### References

[1] Pewowaruk RJ, Gepner AD. Smooth muscle tone alters arterial stiffness. J Hypertens 2022; 40: 512–519.

[2] Pewowaruk RJ, Hein AJ, Carlsson CM, Korcarz CE, Gepner AD. Effects of nitroglycerin Induced vasodilation on elastic versus muscular artery stiffness in older veterans. Hypertens Res 2022; [accepted].

[3] Carlson BE, Secomb TW. A theoretical model for the myogenic response based on the length–tension characteristics of vascular smooth muscle. Microcirculation 2005; 12: 327–338.

[4] Herrera AM, McParland BE, Bienkowska A, Tait R, Paré PD, Seow CY. `Sarcomeres' of smooth muscle: functional characteristics and ultrastructural evidence. J Cell Sci 2005; 118: 2381–2392.

**Keywords**: Biomechanics, Vasodilation, Mathematical model, Human carotid artery

### P.063

### Estimation of central aortic pressure waveform and hemodynamic parameters from finger photoplethysmography

**Ahmad Qasem**[1], James Cox[2], Mark Butlin[2], Alberto Avolio[2], Isabella Tan[2]

[1]Atcor Medical, Sydney, Australia, [2]Faculty of Medicine, Health & Human Sciences, Macquarie University, Sydney, Australia

**Background:** Non-invasive estimation of the central aortic pressure waveform is a valuable clinical tool to assess cardiovascular function. Current reliable methods require peripheral pulses from either the radial artery using tonometry or the brachial artery using the oscillometric signal from a pneumatic cuff. This study aimed to estimate and validate the central pressure waveform derived from the finger photoplethysmography (PPG) signal during controlled and altered haemodynamic conditions.

**Methods**: Continuous recordings of radial tonometry (SphygmoCor) and finger PPG signals were obtained during baseline conditions (2 min) and following an isometric hand-grip manoeuvre (3 min) from 34 participants (age: 19–82 years, 18 male, BMI: 18–35 kg/m2, seated systolic and diastolic pressures 95–169 mmHg and 52–106 mmHg, respectively). Central pressure parameters were estimated from PPG and radial signals using more than 300 averaged signals. Participants were divided into a system model estimation group (n = 5) and a test group (n = 29). Comparisons were made between PPG-derived and radial-derived central systolic blood pressure (cSBP), augmentation index (AIx), sub-endocardial viability ratio (SEVR) and pulse pressure amplification (PPamp).

**Results**: The PPG-derived cSBP, AIx, SEVR and PPamp average error was − 3.5 ± 1 mmHg, 3 ± 6%, 4 ± 7%, 1 ± 1.6%, respectively, for the total recordings. All correlations between the PP-derived and radial-derived parameters were > 0.85 for all parameters (p < 0.001). PPG-derived central parameters test results were similar under controlled and altered haemodynamic conditions.

Conclusion: Central hemodynamic parameters can be accurately derived from a finger PPG signal under controlled and altered haemodynamic conditions.

### References

1. Chen CH, Nevo E, Fetics B, Pak PH, Yin FC, Maughan WL, et al. Estimation of central aortic pressure waveform by mathematical transformation of radial tonometry pressure. Validation of generalized transfer function. Circulation. 1997;95:1827–1836.
2. Pauca AL, Wallenhaupt SL, Kon ND, Tucker WY. Does radial artery pressure accurately reflect aortic pressure? Chest. 1992 Oct;102(4):1193–8
3. Pauca A, O'Rourke M, Kon N. Prospective Evaluation of a method for estimating ascending aortic pressure from the radial artery pressure waveform. Hypertension. 2001;38:932–937

**Keywords**: Central aortic pressure waveform, finger photoplethysmography, central hemodynamic parameters, Augmentation Index, isometric hand-grip manoeuvre, central systolic blood pressure, sub-endocardial viability ratio, pulse pressure amplification, Transfer Function

### P.064

### Identification of constitutive arterial tissue parameters using inverse deformation analysis from passive inflation experiments

**Fethi Okyar**[1], Omer Faruk Buyukkaya[1], Cevat Volkan Karadag[1], Bilge Tuna[1]

[1]Yeditepe University, Istanbul, Turkey

**Background:** Changes in the mechanical properties of arteries due to growth, remodeling, or aging are related with cardiovascular diseases. These changes can quantitatively be assessed if a set of suitable biomaterial constitutive parameters could be fitted onto the in vitro response pressure-diameter curve(s). The aim of this study is to determine the multi-variable optimization and parameter identification by using inverse deformation mapping.

**Methods**: For this purpose, in vitro pressure-diameter relations were measured from published experimental results where the excised arterial sample was tethered between axially aligned canulae. An analytical continuum-based computational procedure is defined to pull the current state back to the excised (reference) state. Before applying the multi-variable optimization for parameter identification, the data is first pulled-back using this procedure, if required.

**Results**: In Simon et al. (1970) [1], a pressure-diameter dataset reflecting the internal pressurization (P) stage was provided. However, this data did not include the excised (E) state. When the above procedure was applied to the simple exponential model, a nearly tenfolds decrease in the shear modulus and about 60% increase in the exponential constant were observed as a result of the pull-back to the excised state (coefficient of determination was found as 0.991, for both cases of with and without pull-back). Similar observations were accounted for in the hyperelastic fibre-reinforced continuum model [2].

**Conclusions:** The parameter identification process may be hindered as a result of an incomplete or partial dataset. Inverse deformation mapping may be used to produce the missing data.

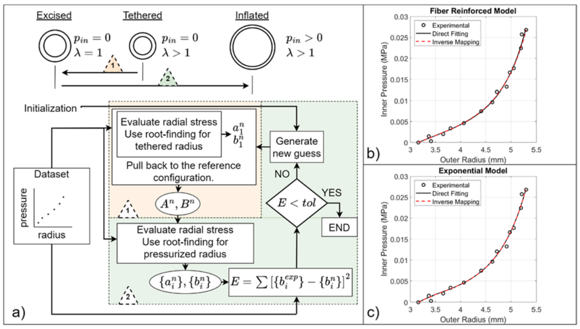

The procedure is depicted in the above diagram (a). Fitting of the exponential model is shown in (b) while the fiber-reinforced (HOG) model is fitted in (c).

### References

[1] Simon, B. R., Kobayashi, A. S., Strandness, D. E., and Wiederhielm, C. A. (June 1, 1971). "Large Deformation Analysis of the Arterial Cross Section." ASME. J. Basic Eng. June 1971; 93(2): 138–145.
[2] Holzapfel, G.A., Gasser, T.C. & Ogden, R.W. A New Constitutive Framework for Arterial Wall Mechanics and a Comparative Study of Material Models. Journal of Elasticity 61, 1–48 (2000).

**Keywords**: Constitutive models, arterial stiffness, inverse deformation, parameter identification

### P.065

### Assessment of large and smal l arteries stiffness in upper and lower limbs amputees: a numerical study

**Hasan Obeid**[1], Vasiliki Bikia[3], Patrick Segers[4], Pierre Boutouyrie[5], Nikos Stergiopulos[3], Mohsen Agharazii[1,2]

[1]Chu De Quebec, Quebec, Canada, [2]Division of Nephrology, Department of Medicine, Faculty of Medicine, Université Laval, Quebec, Canada, [3]Laboratory of Hemodynamics and Cardiovascular Technology, Swiss Federal Institute of Technology, Lausanne, Switzerland, [4]bioMMeda – Institute for Biomedical Engineering and Technology, Ghent University, Ghent, Belgium, [5]AP-HP, Pharmacology Unit, Hôpital Européen Georges Pompidou, University Paris Descartes, Sorbonne Paris Cité, Paris, France

**Arterial** stiffness, as assessed via pulse wave velocity (PWV), has been related to increased cardiovascular morbidity and mortality but has not been previously sufficiently evaluated in amputees. In the present study, we investigated the intrinsic effect of biomechanical alterations caused by limb-amputation on arterial stiffness.

We used a detailed 1D arterial network model coupled with heart model. The PWV was determined by measuring the foot-to-foot pulse transit time. We calculated arterial stiffness of large, medium, and small-sized vessels via carotid-femoral PWV (cfPWV), carotid-radial PWV (crPWV), and radial-digital PWV (rdPWV) in five different settings:1) healthy subject (complete model with upper and lower limbs present), 2) right leg amputee (right lower-limb arteries were removed from the model), 3) two legs amputee (right and left lower-limb arteries were removed from the model), 4) two legs and one-hand amputee (right and left lower-limb and right upper-limb arteries were removed from the model), 5) two legs and two hands amputee (right and left lower and upper limbs were removed from the model).

In this numerical model, output cfPWV's were 6, 6.9, 7.5, 8.2 and 9 m/s respectively for setting 1,2,3 and 4. The crPWV's were 6.3, 7.1 and 7.8 m/s respectively for setting 1,2 and 3. The rdPWV's were 10, 10.7 and 10.9 m/s respectively for setting 1,2 and 3.

These simulations suggest, that with incremental limb amputations, there is a stepwise increase in arterial stiffness, which is relatively more pronounced for aorta. Further analyses are needed to mimic more realistic setting.

**Reference**

Obeid H, Bikia V, Fortier C, Paré M, Segers P, Stergiopulos N, et al. Assessment of Stiffness of Large to Small Arteries in Multistage Renal Disease Model: A Numerical Study. Front Physiol. 2022;13:832,858.

**Keywords**: Arterial stiffness, 1-D modelling, limb amputees

### P.066

### Experimental arterial models (phantoms) that stiffen when distended: a structural design and direct 3D-print approach

**Bruce Guest**[1], Luis Arroyo[1], John Runciman[1]

[1]University Of Guelph, Guelph, Canada

**Background:** Despite the arterial pulse wave's pathophysiologic importance its basis is not fully elucidated[1]. Experimental cardiovascular modelling is useful in arterial mechanical and haemodynamic research[2]; however, current phantom construction techniques limit replication of arterial elastic and anatomic complexities[3]. The elastic-pressure response of phantoms incorporating longitudinal structural corrugations was investigated.

**Methods**: Polyester-polyurethane phantoms (160 mm length) were printed with a fused filament fabrication 3D printer. Five designs differing by corrugation number or magnitude (B-F) were compared to a traditional smooth wall phantom (A). Diametrical compliance behavior was observed under quasistatic hydraulic inflation and pulse wave velocities were measured over a range of mean pulse wave pressures.

**Results**: As luminal pressure increased (5–35 mmHg), corrugated phantom diametrical compliance decreased ($p<0.01$) whereas smooth wall phantom compliance did not. Compliance was axially anisotropic ($p<0.05$), increased in the axial mid-span and towards the upper build height. Corrugated phantom pulse wave velocities increased (1.7–4 m/s) as did pulse wave velocity slopes ($p<0.01$) with increased mean wave pressure (5–40 mmHg). Pulse wave velocity was lower in the 50 vs 100 mm axial mid-span region ($p<0.01$).

**Conclusions**: As determined by quasistatic diametrical compliance and pulse wave velocity, corrugated phantom circumferential elastic response was consistent with the physiologic behaviour of arteries, stiffening with increasing pressure. Elasticity varied significantly with wall design ($p<0.05$); however, height associated printing artefacts decreased while end fixation increased compliance. The functional structure wall approach is novel in arterial phantom construction and further development will improve the utility of phantoms in pulse wave behavior research.

### P.067

### Beat-to-beat variability of invasive pulse wave velocity: implication for the validation of non-invasive devices

**Alessandro Giudici**[1,2], Andra Grillo[3], Filippo Scalise[4], Koen D Reesink[1], Tammo Delhaas[1], Paolo Salvi[5], Gianfranco Parati[5,6], Bart Spronck[1,7]

[1]Department of Biomedical Engineering, CARIM School for Cardiovascular Diseases, Maastricht University, Maastricht, The Netherlands, [2]GROW School for Oncology and Reproduction, Maastricht University, Maastricht, The Netherlands, [3]Department of Medicine, Surgery and Health Sciences, University of Trieste, Trieste, Italy, [4]Department of Interventional Cardiology, Policlinico di Monza, Monza, Italy, [5]Department of Cardiology, Istituto Auxologico Italiano, IRCCS, Milan, Italy, [6]Department of Medicine and Surgery, University of Milano-Bicocca,, Italy, [7]Macquarie Medical School, Faculty of Medicine, Health and Human Sciences, Macquarie University, Sidney, Australia

**Background:** Invasive foot-to-foot pulse wave velocity (PWV) shows beat-to-beat variability due to acute changes in haemodynamic conditions and data processing issues. Though current device validation guidelines suggest averaging over $n\_beats \geq 3$ heartbeats to cope with variability [1], quantitative data on PWV's beat-to-beat variability is lacking. We aimed to quantify this variability and its impact on the confidence of the PWV estimate for intersecting tangent (PWVIT) and second derivative (PWV2nd) foot detection methods.

**Methods**: Pressure waveforms were simultaneously acquired in $n=40$ individuals in the ascending aorta and iliac bifurcation via intra-aortic catheters. We calculated PWV_IT and PWV_2nd over $m=40$ consecutive heartbeats and used Kernel density plots to visualise the variability and distribution of PWV values. Furthermore, we estimated how averaging over n_beats (with n_beats = 2 to 40) affects the standard deviation (SD) of such n_beats-averaged assessment of PWV.

**Results**: PWV_IT was significantly higher (10.40 ± 2.65 vs 10.00 ± 2.65 m/s, mean ± SD, $p=0.015$) and showed lower beat-to-beat SD than PWV_2nd (0.52 ± 0.33 vs 0.62 ± 0.32 m/s, $p=0.046$). This is also visible in subject-specific density plots of PWV_IT (Figure, Panel A) and PWV_2nd (Panel B), and in the average plots in Panel C. Increasing n_beats from 2 to 40 decreased the effective SD from 0.36 to 0.08 m/s for PWV_IT and from 0.44 to 0.10 m/s for PWV_2nd (Panel D). For n_beats = 3 (i.e., the guidelines' lower limit), SD = 0.29 (PWV_IT) and 0.36 m/s (PWV_2nd).

**Conclusions**: Although invasive PWV shows considerable beat-to-beat variability (~ 0.5–0.6 m/s), said variability is reduced by ~ 70% when n_beats = 10, providing a reliable measurement for validation studies.

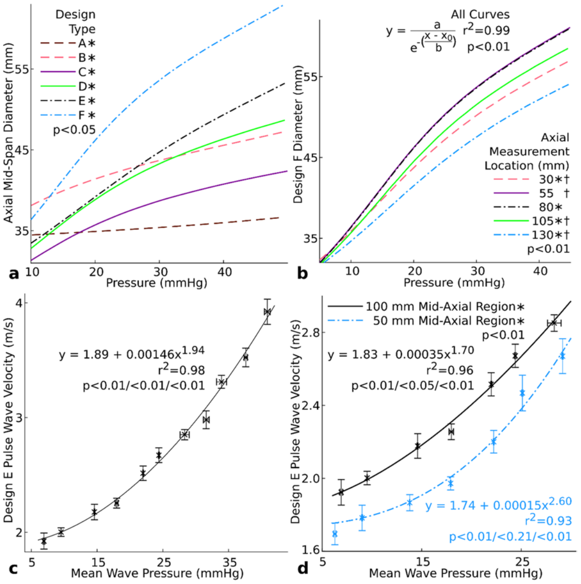

a) Corrugated design diametrical compliance (B-F) varies and decreases with pressure. b) Axial compliance anisotropy. c) Pulse wave velocity increases with pressure and d) is lower in more compliant region.

### References

1. Segers, P, O'Rourke, MF, Parker K, et al. Towards a consensus on the understanding and analysis of the pulse waveform: results from the 2016 workshop on arterial hemodynamics: past, present and future. Artery Res 2017; 18: 75–80. https://doi.org/10.1016%2Fj.artres.2017.03.004
2. Lillie, JS, Liberson, AS, Mix, D, et al. Pulse wave velocity prediction and compliance assessment in elastic arterial segments. Cardiovasc Eng Technol 2015; 6(1): 49–58. https://doi.org/10.1007/s13239-014-0202-x
3. Ionita, CN, Mokin, M, Varble, N, et al. Challenges and limitations of patient-specific vascular phantom fabrication using 3D Polyjet printing. Proc SPIE Int Soc Opt Eng 2014; 13: 9038:90380 M. https://doi.org/10.1117/12.2042266

**Keywords**: Arterial phantom, Arterial compliance, Pulse wave velocity, Pulse wave

### P.068

### Study protocol of a randomized control trial investigating the effects of exercise on endothelial function and pulse wave velocity in the prevention of cardiovascular disease in statin and non-statin users.

**Xela Dafauce Bouzo**[1], Jemima Benson[1], Eric Stöhr[3], James Coulson[2], Barry McDonnell[1], Christopher JA Pugh.[1]

[1]Cardiff Metropolitan University, Cardiff, United Kingdom, [2]Cardiff University, Cardiff, United Kingdom, [3]Leibneiz University, Hannover, Germany

**Background:** Regular exercise is widely recommended to reduce cardiovascular disease (CVD) risk. Recent healthcare guidelines for CVD primary prevention stipulates that individuals with a relatively low risk of CVD (10-year risk score ≥ 10%;) should take a statin. Exercise provides a variety of cardiovascular benefits, including improvements in vascular function. Moreover, statin therapy primarily reduces CVD risk by lowering cholesterol, however, may also improve vascular function. Whilst both therapies can independently reduce CVD risk, the interaction between exercise training and statin therapy on vascular function has never been directly compared in the primary prevention setting.

**Methods**: 80 sedentary male and female participants (40 statin naïve and 40 statin users) aged between 50 and 65 years, with a 10-year CVD-risk score ≥ 10% (estimated via QRISK3) and no established CVD will be recruited onto the study. The statin naïve and statin user groups will be further randomised into the exercise intervention or standard (no exercise) primary care comparator group. The intervention will consist in a 12-week supervised aerobic exercise programme of moderate-intensity. Both groups will complete baseline and 12-week (post intervention) vascular function and structure assessments. Changes in flow-mediated dilation (FMD) and aortic pulse wave velocity (aPWV) will serve as the primary outcome measures. Secondary outcome measures include changes in cardiorespiratory fitness (CPET), carotid intima-media thickness (CIMT), 24-h brachial and aortic blood pressure and lipid profiles.
**Conclusion**: Using a randomised controlled protocol, the study aims to evaluate the interaction between exercise training and statin therapy on vascular structure and function in the primary care setting.

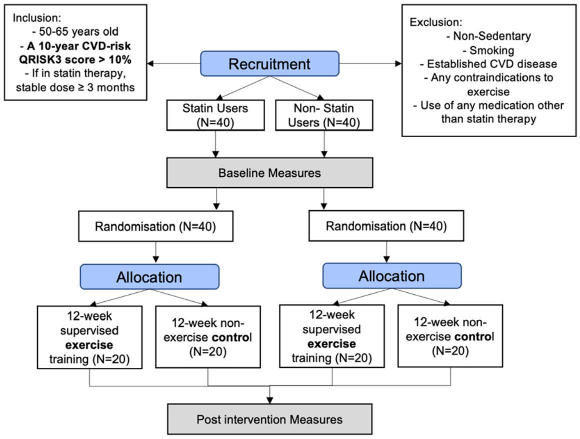

Study flow diagram

**Keywords**: Cardiovascular disease, Primary prevention, Exercise, Vascular function

### P.069

### Unique humanized mouse models of von Willebrand disease type 2A

Marco Heestermans[1], Geneviève Mc Cluskey[1], Ivan Peyron[1], Christelle Reperant[1], Olivier D Christophe[1], Cécile V Denis[1], Peter J Lenting[1], **Caterina Casari**[1]

[1]Inserm U1176, Le Kremlin Bicêtre, France

**Background:** Angiodysplasia is a vascular malformation associated with gastrointestinal bleeding, generally observed in the elderly. This condition is unexpectedly more frequent in patients with von Willebrand disease (VWD)-type 2A having low levels of VWF high-molecular-weight-multimers (HMWMs) and increased VWF-degradation fragments (1).
**Aim**: To develop an innovative murine model of VWD-type 2A and study the role of degraded-VWF in vascular processes.
**Methods**: Mice expressing human (h) VWF, carrying the type 2A (p.R1597W) variant or wild-type (as control) and human GPIbα, have been generated (hVWF(p.R1597W) + / + /hGP1BA + / + and hVWF + / + /hGP1BA + / +). Haemoglobin (Hb), VWF:Ag, propeptide, multimer pattern and factor VIII activity were analyzed. Tail-clip and tail-vein-transection (TVT) bleeding assays were assessed.

**Results**: Control hVWF+/+/hGP1BA+/+-mice expressed 15±4% VWF:Ag, 44±8% FVIII activity and normal VWF multimers. hVWF(p.R1597W)+/+/hGP1BA+/+-mice are viable and do not display spontaneous bleeding manifestations. These mice expressed 3±1% VWF:Ag and 7±1% FVIII activity combined with an abnormal multimer pattern, with only low multimers and few degradation bands visible. Despite the relatively low VWF:Ag levels, hVWF+/+/hGP1BA+/+-mice displayed normal haemostatic responses in both the severe- (tail-clip) and milder- (TVT) bleeding assays. In contrast, hVWF(p.R1597W)+/+/hGP1BA+/+-mice had a severe bleeding phenotype. Interestingly, in the TVT model, although the amount of blood shed was consistent with severe bleeding, 57% of type 2A mice were capable of forming an occlusive, although unstable clot within 15 min of the injury, differing from the bleeding profile of VWF-deficient mice.
**Conclusion**: We developed a unique humanized mouse models for VWD-type 2A. Experiments are ongoing to study the vasculature of these mice.

### Reference

Castaman G, Federici AB, Tosetto A, La Marca S, Stufano F, Mannucci PM, et al. Different bleeding risk in type 2A and 2 M von Willebrand disease: a 2-year prospective study in 107 patients. J Thromb Haemost. 2012 Apr;10(4):632–8.

**Keywords**: VWF, VWD, angiodysplasia, vascular malformation

### P.070

### Pulse waveform-based prediction of vascular calcifications in patients with end stage renal disease

**Urszula**[1], Małgorzata Dębowska[1], Lu Dai[2,3], Abdul Qureshi[3], Magnus Soderberg[4], Bengt Lindholm[3], Peter Stenvinkel[3], Jan Poleszczuk[1]

[1]Nalecz Institute of Biocybernetics and Biomedical Engineering, Polish Academy of Sciences, Warszawa, Poland, [2]Aging Research Center, Department of Neurobiology, Care Sciences and Society, Karolinska Institutet and Stockholm University, Stockholm, Sweden, [3]Renal Medicine and Baxter Novum, Department of Clinical Science, Intervention and Technology, Karolinska Institutet, Stockholm, Sweden, [4]Pathology, Clinical Pharmacology and Safety Sciences, AstraZeneca R&D, Gothenburg, Sweden

**Background:** Medial vascular calcification (VC) is associated with an increased risk of cardiovascular disease and it is particularly prevalent in patients with chronic kidney disease (CKD). Currently, computed tomography is a conventional method of VC assessment. However, exposure to radiation and high costs of the examination are potential concerns. Moreover, it cannot distinguish between medial and intimal VC. Therefore, we propose a novel, non-invasive technique of detecting medial VC in patients with CKD which utilizes brachial pulse wave measurements.
**Methods:** In 97 patients who underwent kidney transplant, medial VC presence was examined in epigastric artery. Additionally, the patients' brachial pulse waves were non-invasively measured (SphygmoCor, AtCor Medical, Australia). We analyzed the waveforms in the frequency domain and extracted features based on the first 20 frequencies. Additionally, patients' characteristics such as age, sex and diabetes were utilized as input variables. An ensemble of three logistic regression models in combination with different subsets of features was built to identify medial VC presence.
**Results**: Results show that the features derived from brachial pulse wave signal contribute to prediction of medial VC presence in CKD patients. The model, assessed using leave-one-out cross-validation, achieved accuracy = 0.91 and F-score = 0.94. Figure 1 shows receiver operating characteristic (ROC) curve of the proposed classifier.
**Conclusions**: In this proof-of-concept research, we showed that medial VC in CKD patients can be detected using the features derived from brachial pulse waveforms. The proposed method is easy to implement and may contribute to a higher accessibility of medial VC detection in CKD patients.

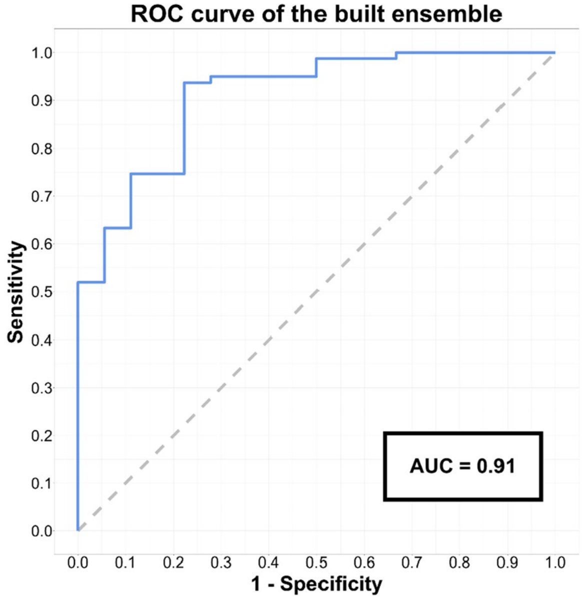

**Fig. 1** Receiver operating characteristic curve of the proposed ensemble

### Reference

Zhang L, Li L, Feng G, Fan T, Jiang H, Wang Z. Advances in CT Techniques in Vascular Calcification. Front Cardiovasc Med. 2021 Sep 29;8:716–822. https://doi.org/10.3389/fcvm.2021.716822. PMID: 34660718; PMCID: PMC8511450.

**Keywords**: Vascular calcifications, machine learning

### P.071

### Brachial pressure gradient as an alternative tool for assessment of endothelial function and cardiovascular disease severity

**Smriti Badhwar**[1,2], Dinu Chandran[1], Ashok Jaryal[1], Chetan Patel[1], Rajiv Narang[1], Kishore Kumar Deepak[1]

[1]All India Institute of Medical Sciences, New Delhi, India, [2]York University, Toronto, Canada

**Introduction:** Flow alteration can affect endothelial function, which is associated with cardiovascular disease (1). Peripheral arterial flow is determined by the pressure gradient between the proximal and distal points in the vessel. The pressure gradient could be a potential alternative for assessing vascular health.
**Methods**: Anterograde and retrograde brachial artery pressure gradients were estimated from the positive and negative components of the first derivative of the non-invasive beat-to-beat brachial pressure waveform and carotid-radial pulse wave velocity in 90 patients with ischemic heart disease. Retrograde flow was assessed using a pulsed-wave doppler. Cardiovascular disease severity was evaluated using Single Positron Emission Computerized Tomography imaging and quantified as %perfusion defect from the summed stress score, using a 20-segment cardiac model. Endothelial function was assessed by ultrasound-based measurement of flow-mediated dilation (FMD).
**Results**: A significant association was seen between retrograde flow velocity (RBFVAUC and RBFVpeak) and retrograde pressure gradient (Rt-dP/dxAUC and Rt-dP/dxpeak) ($r = 0.34$, $p = 0.003$ and $r = 0.38$, $p = 0.0006$ respectively). RBFVAUC and Rt-dP/dxpeak showed a significant negative correlation with %FMD ($r = -0.24$, $p = 0.026$ and $r = -0.22$, $p = 0.047$ respectively). Ratio of retrograde to total pressure gradient (Rt-dP/dxpeak/(At-dP/dxpeak + Rt-dP/dxpeak) showed a significant positive correlation with %perfusion defect ($r = 0.24$, p = 0.025). This association was independent of aortic systolic pressure, age, heart rate and total cholesterol.
**Conclusion**: Brachial pressure gradient is related to pathophysiological alterations in arterial flow and can be incorporated into developing an alternative method for assessing endothelial dysfunction and cardiovascular disease severity.

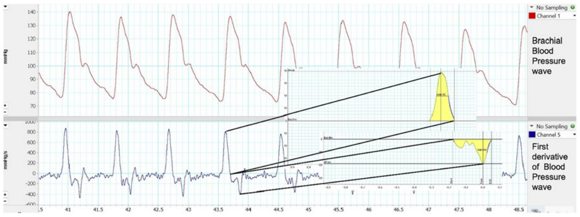

Anterograde and Retrograde brachial pressure gradients were calculated separately from the peak and mean of the first derivative of the brachial pressure waveform and the carotid-radial pulse wave velocity.

**References**
1. Bretón-Romero R, Wang N, Palmisano J, Larson MG, Vasan RS, Mitchell GF, et al. Cross-Sectional Associations of Flow Reversal, Vascular Function, and Arterial Stiffness in the Framingham Heart Study. Arterioscler Thromb Vasc Biol. 2016 Dec;36(12):2452–9.

**Keywords**: Endothelial Function, Retrograde flow, Cardiovascular disease, brachial pressure wave

### Special Populations

### P.081
### Aortic stiffness and systemic inflammation as therapeutic targets to intravitreal anti-vascular endothelial growth factor therapy in patients with age-related macular degeneration

Nikolaos Ioakeimidis[1], Ioanna Gourgouli[2], **Dimitrios Terentes-Printzios**[1], Danai-Magdalini Gourgouli[2], Christos Georgakopoulos[1], Konstantinos Aznaouridis[1], Sofia Spai[2], Dimitrios Tousoulis[1], Konstantinos Tsioufis[1], Charalambos Vlachopoulos.[1]

[1]First Department of Cardiology, Hippokration Hospital, Athens Medical, Athens, Ελλάδα, [2]Ophthalmology Department, General Hospital of Athens "Sismanoglio-Amalia Fleming", Athens, Greece, Athens, Greece

**Background:** Aortic stiffness and inflammation are predictors of cardiovascular risk. Anti-vascular endothelial growth factor agents (anti-VEGF), injected intravitreally, can reverse the course of exudate age-related macular degeneration (AMD). We investigated the association of changes in aortic stiffness and inflammation with response to anti-VEGF therapy.
**Methods**: 54 patients (mean age: 76 ± 10 years) with AMD received two consecutive monthly intravitreal injections of ranibizumab (0.5 mg). The primary outcome measure was change in carotid-femoral pulse wave velocity (PWV) from baseline to 1 month after the second injection.
**Results**: Ranibizumab caused a decrease in PWV after the first (by 0.36 ± 1.4 m/s) and the second injection (by 0.31 ± 1.4 m/s) and remained decreased 1 month after the second injection (overall $P < 0.05$). PWV decreased significantly in good responders (according to clinical criteria and fundus findings, $P = 0.004$), whereas it increased numerically in poor responders ($P = 0.21$) over the study period. In responders, hsIL-6 decreased after the first injection and remained decreased 1 month after the second injection (by 0.63 ± 0.35 pg/ml, overall $P = 0.02$). PWV ($P = 0.005$) and hsIL-6 ($P = 0.042$) were independent predictors of improvement after adjusting for age, hypertension and diabetes. The decrease in PWV throughout the study period correlated with the reduction in hsIL-6 ($r = 0.36$, $P < 0.01$).
**Conclusions**: Intravitreal ranibizumab injections lead to a decrease in PWV and hsIL-6. Both parameters predict clinical improvement and may aid in improving treatment targeting and therapeutic outcome in AMD patients.
**Keywords**: Arterial stiffness, inflammation, anti-VEGF, hypertension

### Vascular aging

### P.091
### Two age-related pathologies: the relation of arterial stiffness to aortic valve stenosis in men and women.

**Renske**[1], Jeannette Goudzwaard[1], Nicolas van Mieghem[1], Peter de Jaegere[1], Francesco Mattace-Raso.[1]

[1]Erasmus MC University Medical Center, Rotterdam, Netherlands

**Introduction:** There are similarities in etiology and pathophysiology between aortic valve stenosis and arterial stiffness. We studied whether arterial stiffness, measured as arterial pulse wave velocity (aPWV), was associated to aortic valve stenosis in both men and women.
**Method**: We included 333 patients (172 men and 161 women) with aortic valve stenosis and information on the aPWV who were included in an ongoing observational cohort study. The aPWV was measured with a brachial cuff-based oscillometric measurement (Mobil-O-Graph 24 h PWA Monitor, I.E.M. Gmbh, Stolberg, Germany). The median aPWV was used to differ between low and high arterial stiffness group. Aortic valve stenosis was assessed with use of an echocardiogram and multi-slice computed tomography. Results were stratified for sex.
**Results**: In men the peak aortic valve velocity and mean aortic valve pressure gradient were both higher in patients with a high aPWV (3,8 m/s vs 4,05 m/s ($P = 0.018$) and 32 mmHg vs 41 mmHg ($P = 0.010$), respectively). In women there were no differences found in diagnostic measurements of aortic valve stenosis between the low or high aPWV groups.
**Conclusion**: We found aortic valve peak velocity and the mean aortic valve pressure gradient was higher in men with a high aPWV. We found no relation between aortic valve stenosis and arterial stiffness in women.

**Figure 1.** Median values of aortic valve stenosis across low or high pulse wave velocity groups for male patients.

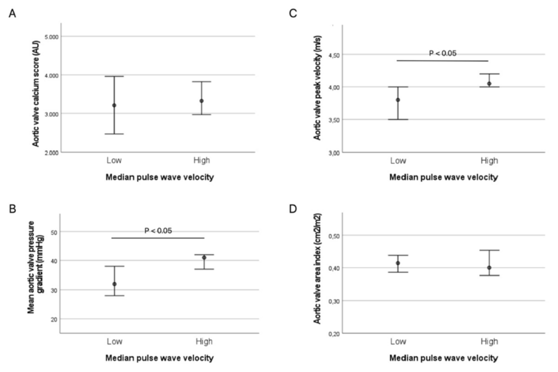

**Notes:** Variables were compared using the Mann Whitney U test. Significant level is 0.05. Dots represent median values and bars represent 95% CI.

**Keywords**: Aortic valve stenosis, Arterial stiffness, Vasculair aging

### P.092

### Carotid-femoral pulse wave velocity variability: Beyond errors in measurements

**Amira Tairi**[1], Hasan Obeid[1], Catherine Fortier[1], Alessandro Giudici[2,3], Bart Spronck[3,4], Mohsen Agharazii[1]

[1]CHU de Québec Research Center, L'Hôtel-Dieu de Québec Hospital, Quebec, Canada, [2]Department of Biomedical Engineering, CARIM School for Cardiovascular Diseases, Maastricht University, Maastricht, The Netherlands, [3]GROW School for Oncology and Reproduction, Maastricht University, Maastricht, The Netherlands, [4]Macquarie Medical School, Faculty of Medicine, Health and Human Sciences, Macquarie University, Sydney, Australia

**Background:** Variability of carotid-femoral pulse wave velocity (CFPWV) measurements may be related to measurement errors, but also to physiological beat-to-beat variations in pulse transit time (TT). We aimed to (1) evaluate beat-to-beat variability of CFPWV on simultaneous non-invasive carotid and femoral waveforms without signal artefacts, and (2) explore its clinical and hemodynamic determinants.
**Methods**: In 44 adult patients (47 ± 18 years; 50% men; 32% hypertensive, 27% with chronic kidney disease, 9% diabetic and 5% with cardiovascular disease), three 10 s-long acquisitions of carotid and femoral pressure waveforms were performed using Complior Analyse. Raw data of the three recordings were extracted, checked to be artefact-free, concatenated, and subjected to a custom 2nd derivative-based foot detection algorithm. Mean, beat-to-beat standard deviation (SD), and coefficient of variation (CV) of CFPWV (80% of direct distance) and heart rate were determined. Regression analysis was used to identify determinants of CV of CFPWV.
**Results**: 44 ± 3 (mean ± SD) beats per individual were analysed, and the mean CFPWV was 7.7 ± 2.6 m/s. The SD and CV of CFPWV were 1.2 ± 0.8 m/s and 13.9 ± 6.5%, respectively. In multivariable regression analysis, age (standardized ß = 0.470, p < 0.001) and intra-individual SD of heart rate (ß = 0.430, p < 0.001) explained 63% of changes in CV of CFPWV. Systolic/diastolic blood pressures were not significant determinants of CV of CFPWV.
**Conclusions**: There is a variability in beat-to-beat pulse transit time that is not explained by poor signal quality, but by higher physiological variations of beat-to-beat transit time, which is explained by advancing age and beat-to-beat heart rate variability.
**Keywords**: Signal analysis, pulse wave velocity algorithms

### P.093

### Pulse wave velocity: intersecting tangents versus second derivative on same pressure wave recordings.

**Amira Tairi**[1,2], Hasan Obeid[1,2], Catherine Fortier[1,2], Mathilde Paré[1,2], Nadège Côté[1,2], Emy Philibert[1,2], Charles-Antoine Garneau[1,2], Rémi Goupil[3,4], Mohsen Agharazii[1,2]

[1]CHU de Québec Research Center, L'Hôtel-Dieu de Québec Hospital, Quebec, Canada, [2]Division of Nephrology, Faculty of Medicine, Laval University, Quebec, Canada, [3]Department of Medicine, Université de Montréal, Montreal, Canada, [4]Hopital du Sacré-Cœur de Montréal Research Center, Montreal, Canada

**Background:** Aortic stiffness is assessed by determination of pulse wave velocity using pulse transit time and the distance between carotid and femoral arteries. Transit time is obtained by using intersecting tangents algorithm or the point of maximal upstroke during systole (2nd derivative). Millasseau et al. [1] have proposed a formula for converting transit time between methods using SphygmoCor (intersecting tangents) and the Complior SP (2nd derivative). The objective of the present study is to compare the two methods using the same pressure waveforms obtained by the newer generation of Complior and using Millasseau's formula.
**Methods**: In a cross-sectional study of heterogeneous group of subjects, aortic stiffness was assessed by the Complior Analyse device using 2nd derivative. The pulse waveforms were extracted and used for analysis by custom MATLAB algorithm for intersecting tangents, and the results were then compared to Millasseau's formula.
**Results**: The preliminary results of the first 44 patients (men: 50%; mean age: 47 ± 18 years) show that Millasseau's formula underestimates the transit times values by about 6% in comparison with the transit times obtained by the intersecting tangents method using MATLAB software (63 ± 23 ms vs 67 ± 21 ms; P < 0.001). This results in an overestimation of the pulse wave velocities values by about 10% (11.1 ± 5.2 m/s vs 10.2 ± 3.6 m/s; P < 0.001).
**Conclusions**: Based on these preliminary results, the values of pulse wave velocities obtained with Millasseau's formula overestimate values as compared to the values obtained by intersecting tangents method. Therefore, there is a need for a better conversion formula.

$$\Delta t_{\text{intersecting tangent}} = \frac{\Delta t_{\text{maxmal upstroke}} - 14.96}{0.8486}(\text{ms})$$

#### References

1. Millasseau SC, Stewart AD, Patel SJ, Redwood SR, Chowienczyk PJ. Evaluation of carotid-femoral pulse wave velocity: influence of timing algorithm and heart rate: Influence of timing algorithm and heart rate. Hypertension [Internet]. 2005;45(2):222–6. Available from: http://dx.doi.org/10.1161/01.HYP.0000154229.97341.d2

**Keywords**: Kidney, Hypertension, Pulse wave velocity measurements, Mathematical algorithms

### P.095

### Carotid artery stiffness in COVID-19 survivors and its relationship with baroreflex sensitivity

**Dinu Santha Chandran**[1], Prachi Srivastava[1], P. M. Nabeel[2], Kiran Raj[3], Jayaraj Joseph[2,3], Kishore Kumar Deepak[1]

[1]Department of Physiology, All India Institute of Medical Sciences, New Delhi, India, [2]Healthcare Technology Innovation Centre, Indian Institute of Technology, Madras, India, [3]Department of Electrical Engineering, Indian Institute of Technology, Madras, India

**Background:** Stiffening of the barosensitive regions of large arteries have been previously linked to baroreflex dysfunction in various patient populations (1). We evaluated the local stiffness of carotid artery in COVID-19 survivors and investigated its relationship with non-invasively assessed baroreflex sensitivity.
**Methods**: Sixty COVID-19 survivors (age range – 22 to 66 years; 27 females) participated in the study at 3–6 months of clinical recovery from RT-PCR positive mild COVID-19. Control group consisted of 53 healthy volunteers matched for age, gender, BMI, and blood pressure whose arterial stiffness data was acquired prior to the onset of COVID-19 pandemic. Stiffness of the common carotid artery was assessed using ARTSENS®—a clinically validated, image-free, ultrasound-based arterial wall tracking technology (2). Heart rate and beat-to-beat blood pressure was non-invasively acquired for 5 min to compute baroreflex sensitivity (BRS) using spontaneous sequence and spectral methods.
**Results**: Pressure-strain elastic modulus – Ep (87.18 ± 25.57 kPa vs 71.42 ± 26.79 kPa; p = 0.0002), and One point pulse wave velocity—PWVβ (5.706 ± 0.7876 m/s vs 5.139 ± 1.011 m/s; p = 0.0001) were significantly elevated in the COVID-19 survivor group in comparison to the historical control group. Spectral estimate of BRS in the high frequency band correlated negatively (r = -0.31; p = 0.016) with PWVβ in the COVID-19 survivor group.

**Conclusions**: Local stiffness of the carotid artery is significantly elevated in COVID-19 survivors at 3–6 months of clinical recovery. Deranged baroreflex function in COVID-19 survivors might be linked to the stiffening of barosensitive regions of central arteries.

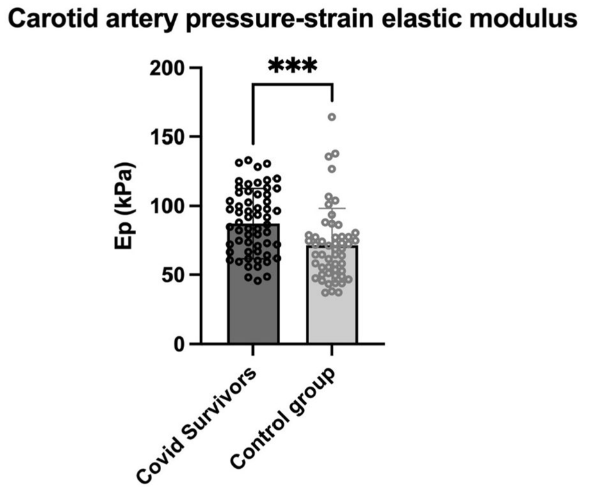

Comparison of Pressure-strain elastic modulus of common carotid artery in Covid survivors versus control group.

**References**

1. Okada Y, Galbreath MM, Shibata S, Jarvis SS, VanGundy TB, Meier RL, et al. Relationship between sympathetic baroreflex sensitivity and arterial stiffness in elderly men and women. Hypertension. 2012 Jan;59(1):98–104.
2. Nabeel PM, Chandran DS, Kaur P, Thanikachalam S, Sivaprakasam M, Joseph J. Association of incremental pulse wave velocity with cardiometabolic risk factors. Sci Rep. 2021 Jul 29;11(1):15,413.

**Keywords**: COVID-19, Arterial stiffness, Baroreflex sensitivity, Carotid artery

### P.097

### Relationship between microangiopathy and macroangiopathy in diabetic patients

**Kornelia Eveilleau**[1], Hester Wessels[2], Hasan Obeid[1], Georges Leftheriotis[4], Magid Hallab[1], Michel Marre[3], Imad Abi-Nasr[1], David Segal[2]

[1]Clinique Bizet, Department of Cardiology, Paris, France, [2]Center for Diabetes and Endicrinology, Houghton Estate, South Africa, [3]Clinique Ambroise Paré, Diabétologie-Endocrinologie, Paris, France, [4]Service de Médecine et d'Exploration Vasculaires, CHU de Nice, Nice, France

**Background:** Diabetes patients are known to produce diabetic retinopathy. Pulse wave velocity (PWV) provides arterial stiffness and hemodynamic parameters. We studied the effect of diabetic retinopathy in different stages on arterial stiffness in diabetics. We measured vascular aging with a new system (pOpmètre) that measures PWV, Ankle-Brachial Index (ABI) and Central Blood pressure.

**Methods**: 83 type 1 diabetes patients attending the clinic (59% male) aged (42 ± 1.54 years). Insulin dependent diabetic patients with retinopathy (N = 61) or without retinopathy (N = 22) with abnormal albuminuria ratio (17%), mean diabetes duration 26 years. All patients underwent vascular assessment with pOpmètre (Axelife—France). The following parameters were measured in each patient, PWV, ABI and central Blood Pressure, non-invasively and without cuff compression.

**Results**: PWV correlated with the degree of retinopathy (F = 13.80; p < 10^(− 4)) and with aging ($r^2$ = 0.25; p < 10^( − 4)), and with diabetes duration ($r^2$ = 0.12; p < 0.002) independently of gender, HbA1c, smoking, BMI, diabetes duration or lipid profile. Aging and diabetes duration were not associated (ANOVA, p = 0.5) to the degree of retinopathy unlike PWV.

**Conclusions**: There is a high interdependence between microvascular and macrovascular lesions in this population of well treated type I diabetic patients with or without retinopathy.

**Reference**

Obeid H, Khettab H, Marais L, Hallab M, Laurent S, Boutouyrie P. Evaluation of arterial stiffness by finger-toe pulse wave velocity: optimization of signal processing and clinical validation. J Hypertens. août 2017;35(8):1618-25.

**Keywords**: Arterial stiffness, diabetes, retinopathy

### P.099

### FRailty and Arterial stiffness – the role of oXidative stress and Inflammation (FRAXI study)

**Ekow Mensah**[1], Khalid Ali[1,2], Winston Banya[3], Frances Ann Kirkham[1], Manuella Mengozzi[2], Pietro Ghezzi[4], Chakravarthy Rajkumar[1,2]

[1]Brighton and Sussex Clinical Trials Unit, University Hospitals Sussex NHS Trust, Brighton, UK, Brighton—East Sussex, United Kingdom, [2]Department of Medicine, Brighton and Sussex Medical School, University of Sussex, Brighton, UK, Brighton, United Kingdom, [3]Research Office, Royal Brompton and Harefield Clinical Group, Guy's and St. Thomas' NHS Foundation Trust, London, UK, London, United Kingdom, [4]Università degli Studi di Urbino, Italy, Urbino, Italy

**Background:** There is an association between frailty and arterial stiffness1. However, arterial stiffness does not uniformly correlate with the spectrum of frailty states. Both oxidative stress and inflammation contribute to vascular aging2. There are no human studies exploring links between arterial stiffness, oxidative stress, inflammaging and frailty.

**Methods**: An observational longitudinal cohort study will be used to examine the association between arterial stiffness, oxidative stress, and inflammation in 50 older adults (≥ 70 years) with clinical frailty scores (CFS) ≤ 6 over six months. Frailty assessments include hand-grip strength, timed-up and go test, mini-mental state examination, geriatric depression scale and sarcopenia using body composition measurements. Arterial stiffness measurements includes carotid-femoral pulse wave velocity and carotid-radial pulse wave velocity using Complior. CAVI device will measure Cardio-ankle vascular index and ankle brachial index. Oxidative stress blood markers nitrotyrosine and 8-hydroxy-2'-deoxyguanosin and inflammation markers high-sensitive C-reactive protein and interlukin-6 will be measured at baseline and 6-months.

**Data Analysis**: Descriptive statistics for continuous data using means and standard deviations for normality distributed variables or medians and inter-quartile ranges for skewed variables will be used. Participants will be categorized into CFS 1–3, and CFS 4–6. Categorical data will use frequencies and comparison between groups. Change in frailty between the groups over 6 months will be compared using paired t-test. Simple linear regression will be done between frailty measures, and exposure variable with significance at p < 0.5.

**Conclusion**: This study data will inform a larger, multi-centre study exploring further the interplay between frailty, biomarkers, and arterial stiffness parameters.

**References**
1. Orkaby, A. R. et al. Cross-sectional association of frailty and arterial stiffness in community-dwelling older adults: The framingham heart study. Journals Gerontol.—Ser. A Biol. Sci. Med. Sci. 74, 373–379 (2019).
2. Inglés, M. et al. Oxidative stress is related to frailty, not to age or sex, in a geriatric population: Lipid and protein oxidation as biomarkers of frailty. J. Am. Geriatr. Soc. 62, 1324–1328 (2014).

**Keywords**: Frailty, arterial stiffness, oxidative stress, inflammaging.

### P.103

### Ideal Life's Simple 7 score relates to carotid intima-media thickness in the healthy population

Gilles Nève[1], Jonathan Wagner[1], Raphael Knaier[1], Denis Infanger[1], Christopher Klenk[1,2], Justin Carrard[1], Timo Hinrichs[1], Henner Hanssen[1], Arno Schmidt-Trucksaess[1], **Karsten Königstein**[1]

[1]University of Basel, Basel, Switzerland, [2]Technical University Munich, Munich, Germany

**Background:** Health scores such as the Life's Simple 7 (LS7) from the American Heart Association and the assessment of carotid intima-media thickness (cIMT) are independently used to predict future cardiovascular health burden. However, evidence of their association remains scarce, especially in healthy populations.
**Methods**: A community sample of the healthy Swiss population aged 50–91 years was included as part of the COmPLETE cohort study. CIMT was measured with a semiautomatic state-of-the-art ultrasound system. The LS7 cardiovascular health score was calculated from body-mass index, cholesterol, systolic blood pressure, hemoglobin A1c, smoking status, physical activity, and diet. For every biomarker two points were given for an ideal health metric level, intermediate scores 1 point, and poor scores 0 points. Intermediate health corresponded to a total of 5–9 points and ideal health to 10–14 points.
**Results**: 280 participants (50.7% male) were included in statistical analyses. Age- and sex-adjusted analyses showed an association of "ideal health" with lower cIMT (−0.038 mm, 95% CI −0.069 mm to −0.007 mm, p = 0.017) compared to "intermediate health".
**Conclusions**: Even in a healthy community-dwelling sample of middle-aged to older adults, individuals with an ideal cardiovascular health score showed more favorable carotid properties than those with an intermediate score. This stresses the relevance of promoting an optimal lifestyle, even among the healthy population, for optimal vascular aging.
**Keywords**: Life's simple 7, arterial stiffness, cardiovascular risk, carotid intima-media thickness

### P.104

### Vascular ageing in relation to chronological and self-perceived age: A Swedish population-based study

Madeleine Johansson[1,2], Peter M Nilsson[1]

[1]Lund University, Malmö, Sweden, [2]Dept. of Cardiology, Skåne University Hospital, Malmö, Sweden

**Background:** Chronological age is a key clinical determinant of aortic stiffness. Self-perceived age (SPA) is a strong predictor of well-being and long-term health[12]. We aimed to investigate the association between SPA, chronological age, and aortic stiffness (vascular ageing) in the general population.
**Methods**: Cross-sectional analysis of a population-based study, Malmö Offspring Study (n = 3563). Mean age 42 ± 14 years, age range 18–74, 53.4% women. Participants completed a self-administered questionnaire related to SPA compared to same-aged/sex peers graded: younger, no difference, older. Aortic stiffness was assessed by carotid-femoral pulse wave velocity (c-f PWV; SphygmoCor), defined as > 10 m/s. Logistic regression models were adjusted for chronological age and sex.
**Results**: Aortic stiffness occurred in 234 (6.6%) subjects. Mean age decreased gradually between all three SPA categories, with the highest mean age observed in subjects who perceived themselves as younger than same-aged/sex peers (49 ± 1 vs. 40 ± 1 vs. 32 ± 1 years, p < 0.001). In crude model, subjects with aortic stiffness perceived themselves as younger than same-aged/sex peers (OR: 0.40, p = 0.002). Adjustment for sex did not change this association (OR: 0.67, p = 0.003). Upon adjustment for sex and chronological older SPA was associated with almost twofold increased likelihood of aortic stiffness (OR: 1.97, p = 0.038). Sex-stratification demonstrated a stronger 2.5-fold likelihood of aortic stiffness in men (OR: 2.50, p = 0.042), but no significant association in women (OR: 1.46, p = 0.43).
**Conclusions**: A negative self-perceived age (feeling older than same-aged/sex peers) is associated with a 2.5-fold increased likelihood of aortic stiffness (vascular ageing) in men when adjusted for chronological age, but not in women.

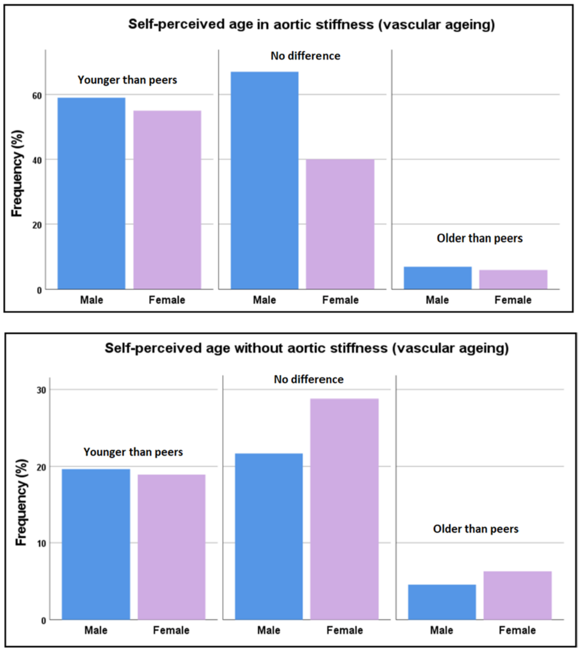

Comparison of self-perceived age with same-aged/sex peers between men and women with and without aortic stiffness (vascular ageing) in the general population.

**References**
1.Hughes ML., Touron DR. Aging in Context: Incorporating Everyday Experiences Into the Study of Subjective Age Front. Psychiatry. 2021.
2.Stephan Y, Demulier V, Terracciano A. Personality, self-rated health, and subjective age in a life-span sample: The moderating role of chronological age. Psychol Aging. 2012.

**Keywords**: Vascular ageing, Aortic stiffness, Epidemiology, Self-perception

### P.105

### Factors affecting short-term repeated measurements of central augmentation index: a randomized cross-over study with two devices

**Mario Podrug**[2], Borna Sunjic[2], Anamarija Bekavac[1], Pjero Koren[1], Varja Đogaš[1], Ivana Mudnic[1], Mladen Boban[1], Ana Jeroncic[1]

[1]University of Split School of Medicine, Split, Croatia, [2]University Department of Health Studies, University of Split, Split, Croatia

**Background:** Central augmentation index (cAIx) is predictive of future cardiovascular events in subjects free of overt cardiovascular disease. Although cAIx can provide clinically useful information beyond brachial blood pressures, its use in longitudinal studies is limited. Data on short-term repeated measurement error and factors affecting it are needed to determine the minimal detectable clinical change of cAIx and reduce this error.

**Methods**: We conducted a longitudinal, block-randomised cross-over study with two observers and two validated devices that use different measurement techniques: SphygmoCor CvMS and Arteriograph; to monitor cAIx changes over 2 weeks. Each participant was recorded 12 times over the course of three visits, separated by one week. During each visit, recordings were taken twice in the morning and twice in the afternoon. Experimental, metreorological and physiological factors affecting cAIx were identified using multilevel mixed-effect models.

**Results**: Participants (N = 35) were uniformly and widely distributed by age (range 20–60 years), BMI (19–39), sex, and hypertensive status. On average, within-subjects cAIx measurements differed by 5.9% (95% CI 5.1–6.8) and 4.7% (4.2–5.3) for SphygmoCor and Arteriograph, respectively. Older age, female sex, and morning recordings significantly increased SphygmoCor's cAIx values. Mean arterial pressure (MAP), outdoor temperature, and their interaction also significantly affected these values but due to interaction, main effects were not unambiguously interpretable ($P \leq 0.029$ for all). For Arteriograph, we found that older age, MAP, shorter height, morning recordings, and 1st visit significantly increased cAIx ($P < 0.001$ for all).

**Conclusion**: We evaluated measurement error of cAIx and made suggestions for reducing it in longitudinal studies.

#### Reference

Vlachopoulos C, Aznaouridis K, O'Rourke M Fet al. Prediction of cardiovascular events and all-cause mortality with central haemodynamics: a systematic review and meta-analysis. Eur Heart J 2010; 31(15): 1865–1871.

**Keywords**: cAIx, measurement error, factors

### P.106

### Validation of an oscillometric device for brachial-ankle PWV: preliminary results

**Maryam Jadoon**, Hakim Khettab, Justin Junior, Pierre Boutouyrie, Rosa-Maria Bruno

[1]Paris cardiovascular research center, Inserm U970,Université Paris Cité, Paris, France

**Introduction:** Arterial stiffness is independent and clinically relevant prognostic biomarker, which can be measured by different techniques, including carotid-femoral Pulse wave velocity (cfPWV), Branchial-ankle PWV baPWV. The aim of this study is to investigate if there is an agreement between ankle-branchial and Carotid-femoral pulse wave velocity.

**Methods**: The carotid- femoral and Branchio-ankle Pulse wave velocity of 30 patients coming for routine clinical checkup of cardiovascular risk evaluation were measured with SphygmoCor CVSM (Atcor Medical, Australia) and WatchBP Office Vascular (Microlife, Widnau, Switzerland). The mean of left and right leg measurements was used for calculating baPWV, whereas the mean of two measurements on the right side (median for 3) was used for cfPWV. The Bland–Altman Analysis was used for finding the agreement between the two types of pulse wave velocities.

**Results**: 30 patients (63% men, age 67 ± 12.75 years, BMI 25.73 ± 3.84 kg/m2, MBP 92.866 ± 10.13 mmHg). CfPWV was 12.25 ± 2.56 m/s and baPWV was 11.67 ± 1.90 m/s. cfPWV and baPWV were linearly correlated with each other ($r = 0.6079$, $p = 0.0004$, Intercept 2.6331, Slope 0.8255). Both were positively correlated with age (cfPWV: $r = 0.6135$, $p = 0.0004$, baPWV $r = 0.4688$, $p = 0.0103$). The two metrics showed a substantial agreement, with no significant bias (bias CI 95% −0.597, 95.0% lower confidence limit −1.367, 95.0% upper confidence limit 0.173).

**Conclusions**: In a population of individuals undergoing cardiovascular screening, a good agreement between cfPWV an baPWV was found. These preliminary results need to be confirmed in a larger cohort.

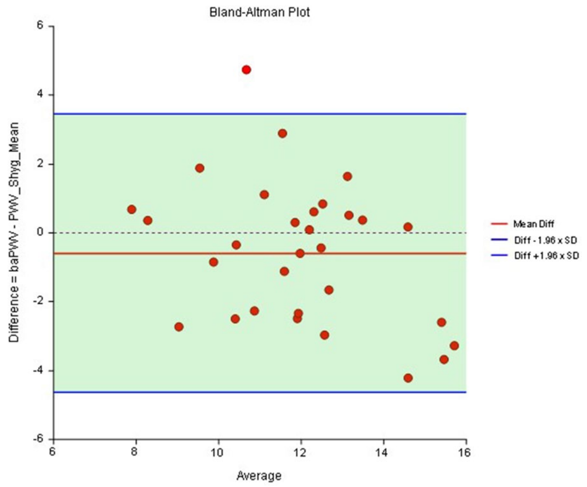

#### References

Kollias A, Kyriakoulis KG, Gravvani A, Anagnostopoulos I, Stergiou GS. Automated pulse wave velocity assessment using a professional oscillometric office blood pressure monitor. The Journal of Clinical Hypertension. 2020 Oct;22(10):1817–23.

Segers P, Rietzschel ER, Chirinos JA. How to measure arterial stiffness in humans. Arteriosclerosis, thrombosis, and vascular biology. 2020 May;40(5):1034–43.

**Keywords**: Vascular ageing device validation, vascular assessment, branchio-ankle pulse wave velocity, pulse wave velocity validation study

### P.110

### Beamforming LDV-data for carotid-femoral pulse-wave velocity estimation

**Simeon Beeckman**[1], Yanlu Li[3,4], Pierre Boutouyrie[5], Nilesh Madhu[2], Patrick Segers[1]

[1]IBiTech-bioMMeda, Ghent University, Ghent, Belgium, [2]IDlab, Ghent University—imec, Ghent, Belgium, [3]Photonics Research Group, Ghent University-imec, Ghent, Belgium, [4]Center for Nano- and Biophotonics, Ghent, Belgium, [5]INSERM U970, Université de Paris, Paris, France

**Background:** Carotid-femoral pulse-wave velocity (cfPWV) has been recognized as a biomarker for arterial stiffness. It is therefore valuable to be able to estimate this value easily and quickly for a wide range of potential patients [1]. We are developing a novel device based on multi-beam laser-doppler vibrometry. It can measure pulse-induced vibrations of high spatial and temporal resolution on bare skin the neck and groin. Two methods of estimating carotid-femoral pulse transit time (cfPTT) were tested.

**Methods**: We applied a dedicated beamforming algorithm to combine and improve the data from 6 parallel signals of simultaneous measurements at both carotid and femoral measurement sites. This was done on a subset of N = 54 high-quality carotid-femoral LDV measurements [2]. We then calculated cfPTT (1) using all pair-wise combinations of the raw signals from all channels (brute-force method) and (2) using the beamformed signals. The final cfPTT estimate, in each case, was computed as the average of all estimated cfPTT's per dataset. These cfPTT's were then compared to reference cfPTT's (Sphygmocor system).

**Results**: As the number of generated cfPTT's in a given measurement increased (> 75), so did the correspondence of the final cfPTT estimate with the reference (see Fig. 1). The same effect was observed with increasing number of timepoints (> 5) at which a cfPTT was able to be calculated. This held true for cfPTT's estimated using both beamforming and brute-force techniques.

**Conclusions**: Accurate cfPTT estimates are obtained for good-quality LDV-measurements, where sufficient discernable heartbeats were recognized using the beamforming and brute-force methods.

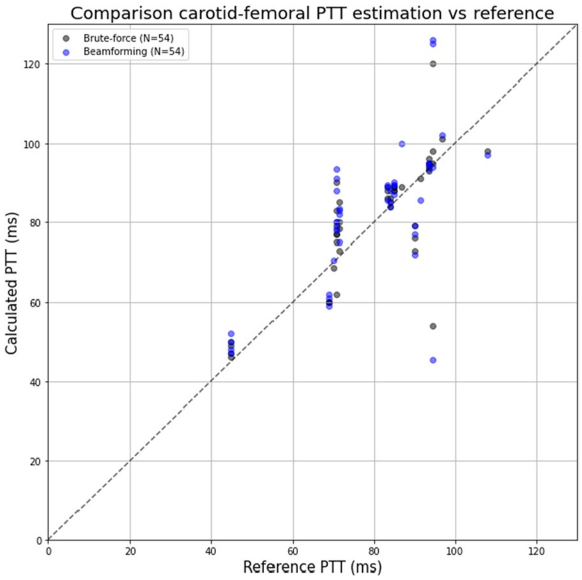

**Fig. 1** A comparison of PTT estimates in qualitative carotid-femoral LDV measurements with their reference PTT's. Estimates made via brute-force method are shown in black, via beamforming in blue

**References**

[1] Segers P, Rietzschel ER, Chirinos JA. How to Measure Arterial Stiffness in Humans. Arterioscler Thromb Vasc Biol. 2020;40(5):1034–1043. http://doi.org/10.1161/ATVBAHA.119.313132

[2] Seoni S, Beeckman S, Li Y, et al. Template Matching and Matrix Profile for Signal Quality Assessment of Carotid and Femoral Laser Doppler Vibrometer Signals. Front Physiol. 2022;12:775,052. Published 2022 Jan 11. http://doi.org/10.3389/fphys.2021.775052

**Keywords**: Laser Doppler Vibrometry (LDV), Carotid-femoral pulse-wave velocity (cfPWV), Pulse transit-time (PTT) estimation, Beamforming.

### Vascular biology and pathophysiology

### P.121

### Direct link between aortic displacement and carotid artery longitudinal wall behaviour: a cadaver case study

**Kailey Stevens**[1], Chloe Athaide[1], Cindy van Loo[2], Tamara Maciel[1], Bart Spronck[2,3], Jason Au[1]

[1]University Of Waterloo, Waterloo, Canada, [2]Maastricht University, Maastricht, Netherlands, [3]Macquarie University, Sydney, Australia

**Background:** Carotid artery longitudinal motion (CALM) describes movement of the arterial wall with (anterograde, forward) and against (retrograde, backward) blood flow[2]. Determinants of beat-to-beat regulation of CALM are not fully understood. Influences of blood flow and cardiac contraction on CALM have previously been investigated[1], although separation of these factors in vivo is challenging. Human cadaveric specimens allow the opportunity to view the isolated impact of central force application versus the influence of shear forces on longitudinal motion. Accordingly, we aimed to study the potential influences of central cardiovascular factors on longitudinal wall motion.

**Methods**: A thoracic dissection of a 19-year-old male donor was performed to reveal the ascending aorta. A clamp fastened in series to a load cell was attached to the aortic root, and caudal force was applied up to 11.5 and 13.2 N over 3.5 s. Longitudinal wall displacement was measured via vascular ultrasound at the left common carotid artery.

**Results**: Longitudinal wall displacement was measured as 1.44 mm and 1.27 mm, for each respective force application. The longitudinal pre-stretch value was 1.24 for the left common carotid artery (3.00 cm in situ, 2.41 cm excised)[3]. For comparison, the representative maximum displacement for a 19-year-old active male is 0.70 mm over a single cardiac cycle.

**Conclusion**: Caudal force application on the aorta generates a trace similar to the retrograde phase seen in CALM. This is the first evidence to suggest direct influence of cardiac contraction on retrograde motion, providing a plausible mechanistic theory on 2D arterial wall motion[1].

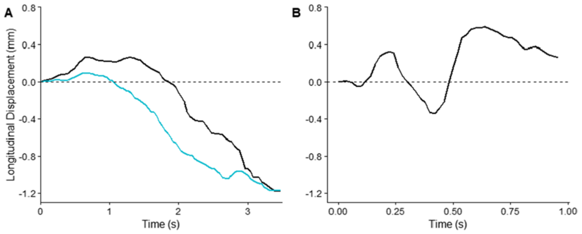

A: longitudinal displacement from 11.5 N (black) and 13.2 N (blue) force applications; B: representative CALM trace over a single cardiac cycle for a 19-year-old male.

**References**

1. Athaide CE, Spronck B, Au JS. Physiological basis for longitudinal motion of the arterial wall. American Journal of Physiology-Heart and Circulatory Physiology. 2022 May 1;322(5):H689-701.
2. Cinthio M, Ahlgren AR, Bergkvist J, Jansson T, Persson HW, Lindstrom K. Longitudinal movements and resulting shear strain of the arterial wall. American Journal of Physiology-Heart and Circulatory Physiology. 2006 Jul;291(1):H394-402.
3. Horný L, Netušil M, Voňavková T. Axial prestretch and circumferential distensibility in biomechanics of abdominal aorta. Biomechanics and modeling in mechanobiology. 2014 Aug;13(4):783–99.

**Keywords**: Wall behaviour; pre-stretch; stiffness

### P.122

### An increase in circulating Angiotensin 1–7 levels post-angiotensin-converting enzyme inhibition is associated with delayed vascular ageing and improvement in baroreflex function in type 2 diabetic patients with hypertension

**Prachi** [1], Dinu S Chandran[1], Viveka P Jyotsna[1], Ashok Kumar Jaryal[1], Kishore Kumar Deepak.[1]

[1]Department of Physiology, All India Institute of Medical Sciences, New Delhi, India

**Background:** Angiotensin 1–7 (Ang1-7) is a novel peptide which has a vaso-protective role, as reported in animal studies(1–3). However, it's role in human vascular ageing is not fully known. We evaluated association between the increment in Ang1-7 levels and vascular functions, post ACE inhibition.

**Methods**: Sixty diabetic hypertensive patients (mean age 46.2 ± 8.1 years) participated in the study. Beat-to-beat blood-pressure and electrocardiogram were recorded for 5-min to compute baroreflex sensitivity. Circulating levels of angiotensinII, angiotensin1-7, Angiotensin-Converting-Enzyme 2 (ACE2), hsCRP and Interleukin-10 (IL-10) were measured using ELISA. Flow-mediated-dilation (FMD) of Brachial artery and carotid-intima-media-thickness (CIMT) were measured by ultrasonography. Carotid-femoral Pulse-wave-velocity (cf-PWV) and Augmentation-Index (AIx) were measured using applanation tonometry.

**Results**: Patients were categorized into two groups based on the incremental changes in Ang1-7 from baseline to 3 months (3 M-BL) post ACE inhibition. The comparisons were made between the highest (Group-1) and the lowest (Group-2) quartiles. Greater improvement was observed in systolic, diastolic ALL-BRS and FMD in group-1 when compared to group-2 [SBP-ALL-BRS3M-BL(ms/mmHg):6.1 ± 4.1 Vs 1.6 ± 7.3, p = 0.02; DBP-ALL-BRS3M-BL(ms/mmHg):9 ± 9.6 Vs 1.2 ± 7.4, p = 0.007; FMD3M-BL(cm.):0.02 ± 0.01 Vs 0.01 ± 0.01, p = 0.006]. cf-PWV, CIMT and AIx showed greater decrement in group-1 in comparison to group-2 [cf-PWV3M-BL(m/s):(-3.3) ± 2.2 Vs (− 0.39) ± 2.9, p = 0.001; CIMT3M-BL(mm): (− 0.17 ± 0.18 Vs − 0.004 ± 0.13, p = 0.001; AIx3M-BL(%): (− 8.3) ± 8.5 Vs (− 2.4) ± 9.8, p = 0.04]. IL-10 showed a significant increase [IL-103 M-BL(pg/ml):{4.4(1.2–9.04) Vs 0.61 (− 12.2) − 4.09}, p = 0.03] while hsCRP decreased [hsCRP3M-BL(mg/L): (− 6.3) {(− 14.4)–(− 1.7)} Vs (− 0.19) {(− 2.5)–0.78}, p = 0.002] in group-1 compared to group-2.

**Conclusions**: Greater increment in Ang1-7 post-ACE inhibition is associated with improvement in BRS, endothelial function, arterial stiffness and inflammation. Ang1-7 may play a role in delaying vascular ageing.

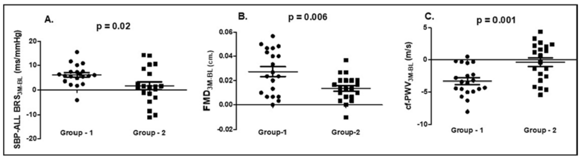

Showing greater improvement in A. SBP ALL-BRS (ms/mmHg), B. Flow-Mediated-Dilatation (cm.), and a decrease in C. cf-PWV (m/s) from baseline to 3 months (3 M-BL) post ACE inhibition.

#### References

1. Valencia I, L. Shamoon, A. Romero, F. De la Cuesta, C.F. Sanchez-Ferrer, C. Peiro. Angiotensin-(1 — 7), a protective peptide against vascular aging. Peptides 152 (2022) 170–775.
2. Fan Jiang, Jianmin Yang, Yongtao Zhang, Mei Dong, Shuangxi Wang, Qunye Zhang, et al. Angiotensin-converting enzyme 2 and angiotensin 1–7: novel therapeutic targets.

Nat Rev Cardiol. 2014 Jul;11(7):413–26.

3. Hossam A. Shaltout, James C. Rose, Mark C. Chappell and Debra I. ANG-(1–7) Deficiency and baroreflex impairment precede the antenatal betamethasone exposure induced elevation in blood pressure. Hypertension. 2012; 59(2): 453–458.

**Keywords**: Angiotensin 1–7, Baroreflex Sensitivity, Arterial Stiffness, Vascular ageing

### P.123

### Carotid arterial wall viscosity and stiffness are increased in type 2 diabetes patients

**Frédéric Roca**[1,2], Louise Zmuda[1,2], Michaela Iacob[1,2], Lucile Moreau-Grangé[3], Gaetan Prevost[3], Robinson Joannides[1,2], Jeremy Bellien[1,2]

[1]Pharmacology, Rouen University Hospital, Rouen, France, [2]INSERM U1096, Rouen, France, [3]Diabetology-Endocrinology, Rouen University Hospital, Rouen, France

**Background:** Type 2 diabetes (T2D) is associated with an increase in arterial stiffness. However, changes in arterial wall viscosity (AWV) during T2D have been little investigated (1,2). Moreover, despite many studies describing an increase in cfPWV, few studies investigated the change in local carotid stiffness during T2D (3). Our aim was to investigate changes in AWV and in local carotid stiffness, considering working conditions, in patients with T2D.

**Methods**: In this cross-sectional, monocentric study we compared 19 middle-aged patients (median age: 65[60–66] years) with T2D to 30 non-diabetic (ND) controls (median age: 56[52–61] years). The pressure-LCSA loop was obtained by carotid tonometry and contralateral carotid echo-tracking. The absolute viscosity (WV), corresponding to the area of the loop, and the relative viscosity (WV/WE), corresponding to the ratio between WV and the elastic energy stored within the arterial wall (WV/WE), were calculated. Carotid geometry, midwall stress, distensibility and elastic modulus were also compared between groups.

**Results**: T2D patients were older and had more frequently hypertension. Internal diameter, pulse and mean central blood pressure were higher in T2D patients but midwall stress was similar to ND. Carotid distensibility was lower and elastic modulus higher in T2D patients. WV (ND: 11[7–18] vs. T2D: 23[16–41] mmHg.mm2, p = 0.007) and WV/WE (ND: 12% [8–17] vs. T2D: 21% [17–25], p < 0.001) were higher in T2D patients even after adjusting for confounding factors such as age, hypertension or midwall stress.

**Conclusions**: Type 2 diabetes is associated with an increase in arterial wall viscosity and in local carotid arterial stiffness.

#### References

1. Giudici A, Palombo C, Kozakova M, Morizzo C, Penno G, Jamagidze G, Della Latta D, Chiappino D, Cruickshank JK, Khir AW. Noninvasive carotid pressure-diameter loops to identify viscoelastic properties in ageing, hypertension and type 2 diabetes. Journal of Hypertension. 2021;39(11):2307–2317.
2. Toutouzas K, Stefanadis C, Tsiamis E, Vlachopoulos C, Tousoulis D, Tsioufis C, Toutouzas P. Aortic pressure-diameter relation in patients with non-insulin dependent diabetes mellitus: new insights. Diabetologia. 2000;43(8):1070–1075.
3. Prenner SB, Chirinos JA. Arterial stiffness in diabetes mellitus. Atherosclerosis. 2015;238(2):370–379.

**Keywords**: Diabetes, arterial wall viscosity, local carotid stiffness

### P.124

### Optogenetic control of PI3K gamma reveals its role in smooth muscle cell contractility.

**Damien Ramel**[1], Marie-Kergulen Sarthou[1], Amandine Wahart[1], Nicole Malet[1], Stéphanie Gayral[1], Matthias Wymann, Muriel Laffargue[1]

[1]Inserm 1297, Toulouse, France

**Background:** PI3Kγ is a major signaling enzyme of the immune and cardiovascular compartments downstream of Gi-coupled GPCR. This kinase is composed of three subunits, one catalytic (p110γ) and one of the two adapters (p84 and p101) and forms two distinct complexes. To date, the molecular mechanism underlying PI3Kγ functions and the implication of the two PI3Kγ complexes is still unclear.
**Methods**: Here, using optogenetic manipulation of each PI3Kγ complexes, primary cells and original mice models, we investigate the selective functions of the p101 and p84 PI3Kγ complexes in order to modulate the cellular PI3Kγ-dependent processes.
**Results**: We demonstrate that the p84/p110γ but not p101/ p110γ complex control cell contractility through its kinase activity and calcium signaling in human vascular smooth muscle cells. Moreover, we demonstrated that p84 but not p101 is specifically engaged under angiotensin II stimulation, a typical regulator of VSMC function. Finally, new mouse models of p84 and p101 invalidation allowed us to demonstrate the p84/p110γ critical role in VSMC contractile phenotype maintenance in primary cells and characterize the in vivo consequences of p84 deletion in entire aortas.
**Conclusions**: Altogether, our study shed in light how a particular PI3K-adaptor module could differentially control key physiological responses according to its regulatory partner.

#### References

Lupieri, A., R. Blaise, A. Ghigo, N. Smirnova, M.K. Sarthou, N. Malet, I. Limon, P. Vincent, E. Hirsch, S. Gayral, D. Ramel, and M. Laffargue. 2020a. A non-catalytic function of PI3Kgamma drives smooth muscle cell proliferation after arterial damage. J Cell Sci. 133.

Lupieri, A., N.F. Smirnova, R. Solinhac, N. Malet, M. Benamar, A. Saoudi, I. Santos-Zas, L. Zeboudj, H. Ait-Oufella, E. Hirsch, P. Ohayon, T. Lhermusier, D. Carrie, J.F. Arnal, D. Ramel, S. Gayral, and M. Laffargue. 2020b. Smooth muscle cells-derived CXCL10 prevents endothelial healing through PI3Kgamma-dependent T cells response. Cardiovascular research. 116:438–449.

**Keywords**: PI3K, Smooth muscle cells

### P.126

### Effects of lower-extremity digital subtraction angiography on arterial stiffness and metabolome in patients with peripheral artery disease

**Holger Post**[1,2], Kaido Paapstel[1,3,4], Kalle Kilk[5,6], Aigar Ottas[5,6], Anneli Piir[5,6], Jaak Kals[1,2,5,6,7]

[1]Endothelial Research Centre, University of Tartu, Tartu, Estonia, [2]Department of Surgery, Institute of Clinical Medicine, University of Tartu, Tartu, Estonia, [3]Department of Cardiology, Institute of Clinical Medicine, University of Tartu, Tartu, Estonia, [4]Heart Clinic, Tartu University Hospital, Tartu, Estonia, [5]Department of Biochemistry, Institute of Biomedicine and Translational Medicine, University of Tartu, Tartu, Estonia, [6]Centre of Excellence for Genomics and Translational Medicine, Tartu, Estonia, [7]Surgery Clinic, Tartu University Hospital, Tartu, Estonia

**Background:** Arterial stiffness has been shown to predict future cardiovascular events and mortality [1,2]. Assessment of metabolites in biological systems has been increasingly applied to several diseases, leading to recent discoveries in disease-specific biomarkers and their mechanistic implications. The potential effect of angiographic studies with iodine contrast on arterial stiffness and metabolome has not yet been addressed in the literature. The aim of this study was to provide insight into the subacute effects of digital subtraction angiography (DSA) on arterial stiffness and metabolomic profiles of patients with peripheral artery disease (PAD).
**Methods**: 32 male patients with symptomatic PAD (aged $62 \pm 9$ years) undergoing DSA for the assessment and/or endovascular therapy of lower-extremity arteries were studied. Aortic pulse wave velocity, a gold standard indicator of arterial stiffness, was measured at baseline and 24 h after DSA. Venous blood samples were drawn from subjects at baseline, 2 h after DSA and 24 h after DSA, and analysed primarily for metabolic alterations.
**Results**: No statistically significant subacute influence on arterial stiffness was observed in our study. Various shifts in metabolome were observed 2 h and 24 h after DSA. The iodine contrast dose administered during DSA independently influenced the levels of two low-molecular metabolites at 2 h after DSA: lysophosphatidylcholine a C20:3 and putrescine.
**Conclusions:** The results appear to be reassuring for the general safety of DSA in patients with PAD and provide some novel insight into DSA-s effect on the metabolome of these patients.

#### References

1.Vlachopoulos C, Aznaouridis K, O'Rourke M, Safar ME, Baou K, & Stefanadis C. Prediction of cardiovascular events and all-cause mortality with central haemodynamics: A systematic review and meta-analysis. European Heart Journal 2010;31(15):1865–1871.

2.Ben-Shlomo Y, Spears M, Boustred C et al. Aortic pulse wave velocity improves cardiovascular event prediction: an individual participant meta-analysis of prospective observational data from 17,635 subjects. J Am Coll Cardiol 2014;63:636–646.

**Keywords**: Peripheral artery disease, Digital subtraction angiography, Metabolome, Arterial stiffness

### P.127

### Focal adhesions modulate aortic viscoelasticity under altered pulsatile conditions

**Cédric Neutel**[1], Callan Wesley[1], Guido De Meyer[1], Wim Martinet[1], Pieter-Jan Guns[1]

[1]University of Antwerp, Edegem, Belgium

**Introduction:** The aortic wall is composed of different functional elements, such as vascular smooth muscle cells (VSMCs), that together define its viscoelastic properties. This study aimed to investigate how VSMCs influence the viscous and elastic properties of aortic tissue and whether the focal adhesion – F-actin axis is involved.
**Methods**: Aortic segments from C57Bl6/J mice were mounted in a Rodent Oscillatory Set-up for Arterial Compliance (ROTSAC) and subjected to high frequency cyclic stretch. Diastolic and systolic diameter as well as the Peterson modulus (Ep), as a measure of aortic stiffness, were determined. Viscous modulus (Eη) was extracted from pressure-diameter tracings by eliminating loop hysteresis. Afterwards, the elastic modulus (EE) was calculated as the slope of the resulting pressure-diameter tracing. Phenylephrine (2 μM, PE) was used to elicit VSMC contraction. PP2 (10 μM) and cytochalasin D (10 μM, CytoD) were used to inhibit focal adhesion and F-actin function, respectively. The thoracic ascending aorta (ASC) and the abdominal infrarenal aorta (AIA) were investigated in parallel.
**Results**: PE increased both Eη and EE this effect was more pronounced in the AIA as compared to the ASC, indicating a larger impact of VSMC tonus in distal aortic regions. Moreover, increasing pulsatile load by increasing pulse frequency from 1 to 25 Hz decreased Eη. The effect of pulse frequency was attenuated by both PP2 and CytoD. High pulsatile load decreased the contractility of aortic segments.
**Conclusion**: The focal adhesion—F-actin axis responds to altered pulsatile conditions, modulating the viscous properties of aortic tissue.
**Keywords**: Viscoelasticity, Vascular Smooth Muscle Cells, Focal Adhesion, F-actin

### P.128

### Existing bias between vascular ultrasound echo-tracking systems: Switching devices warrants a comparison

**Afrah Malik**[1], Alessandro Giudici[1,5], Koen van der Laan[1], Jos op't Roodt[2], Werner Mess[3], Tammo Delhaas[1], Bart Spronck[1,4], Koen Reesink[1]

[1]Department of Biomedical Engineering, CARIM School for Cardiovascular Diseases, Maastricht University, Maastricht, Netherlands, [2]Department of Internal Medicine, CARIM School for Cardiovascular Diseases, Maastricht University, Maastricht, Netherlands, [3]Department of Clinical Neurophysiology, CARIM School for Cardiovascular Diseases, Maastricht University, Maastricht, Netherlands, [4]Macquarie Medical School, Faculty of Medicine, Health and Human Sciences, Macquarie University, Sydney, Australia, [5]GROW School for Oncology and Reproduction, Maastricht University, Maastricht, Netherlands

**Purpose:** The Esaote MyLab70 + ART.LAB ultrasound system has been extensively used to evaluate arterial properties. Since the system reached its end-of-service-life, ongoing studies are forced to switch devices, with some opting for the MyLabOne [1]. Bias might exist between the two systems, which, if uncorrected, potentially leads to misinterpretation of results. The present study aims to evaluate potential bias between these two devices. Moreover, we also aim to compare two identical MyLabOne systems.
**Methods**: Using a phantom set-up consisting of a silicone tube (for diameter and wall thickness measurements) and an eccentric wheel (for distension measurement), we performed $n = 60$ and $n = 40$ measurements for the inter- and intra-system model comparisons, respectively. Statistical significance was evaluated using independent t-tests.
**Results**: Both comparisons led to significant biases for diameter (relative bias: −0.27%, $p = 0.001$ and −0.30%, $p < 0.001$ for inter- and intra-system comparisons, respectively) and wall thickness (relative bias: 0.38%, $p = 0.004$ and −1.23%, $p < 0.001$ for inter- and intra-system comparisons, respectively), but not for distension (relative bias: 0.48%, $p = 0.333$ and −0.12%, $p = 0.892$ for inter- and intra-system comparisons, respectively).
**Conclusion**: Biases in diameter and wall thickness measurements were observed between ultrasound systems, regardless of whether these systems were (apparently) identical. Biases estimated here can therefore not be generalized to any other pair of similar systems. Therefore, longitudinal studies with large sample sizes that switch between systems should compare their devices to evaluate potential biases and to facilitate robust interpretation of outcomes.

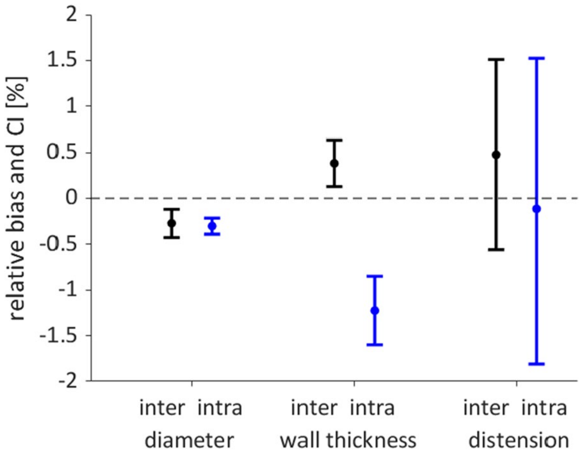

Relative bias and 95% confidence intervals (CI) obtained for inter- and intra-scanner model comparisons for diameter, wall thickness, and distension, normalized with respect to mean values of both devices.

**References**

1. Schram MT, Sep SJ, van der Kallen CJ, Dagnelie PC, Koster A, Schaper N, Henry RMA, Stehouwer CDA, The Maastricht Study: an extensive phenotyping study on determinants of type 2 diabetes, its complications and its comorbidities. Eur J Epidemiol. 2014; 29:439–451.

**Keywords**: Echo-tracking; vascular ultrasound; arterial properties; arterial stiffness

### P.130

### Endothelial glycocalyx degradation depends rather on inflammatory status than hemodynamic conditions

**Mohammad Jahangiri**[1], Nathalie Mercier[1], Simon Toupance[1], Arthur Thomas[1], Carlos Labas[1], Véronique Regnault[1], Athanase Benetos[1], Patrick Lacolley[1], Jeremy Lagrange[1]

[1]Inserm1116, Vandoeuvre-lès-nancy, France

**Background and Objectives**: Glycocalyx, a thin layer of carbohydrates covering endothelial cells, is important for interactions between blood components and the vascular wall. It is implicated in circulating cells adhesion, inflammation, and coagulation regulation and can be damaged in some diseases. The prevailing hypothesis is that hypertension is the primary factor involved in glycocalyx degradation. However, our preliminary data challenge this view and point to a more important role of inflammation. The objective of this study was to assess the respective roles of inflammation and hemodynamic on the endothelial glycocalyx degradation.
**Methods and Results**: Plasma concentrations of syndecan-1, a glycocalyx degradation marker, IL-6, IL-8, IL-10, ICAM-1 and VCAM-1 were quantified by ELISA in 327 participants (62 ± 14 years). Subjects were categorized as atherosclerotic cardio-vascular diseases (ASCVD) patients or controls and performed all a blood pressure and pulse wave velocity assessment. Syndecan-1 was positively associated with circulating IL-6 ($p < 0.001$), IL-8 ($p = 0.002$), and IL-10 concentrations ($p = 0.006$) and with adhesion molecules ICAM-1 and VCAM-1 ($p < 001$). No relation was observed between syndecan-1 and hemodynamic parameters, thus confirming the major role of inflammatory status in the degradation of endothelial glycocalyx. Interestingly, subjects with higher plasma concentration of syndecan-1 (third tertile) displayed more clinical manifestation of atherosclerosis (65 vs 42%; $p < 0.001$) than those with lower concentration (first tertile).
**Conclusions**: Endothelial glycocalyx degradation is rather associated with inflammatory status than hemodynamic parameters. Higher degradation of glycocalyx is associated with increased percentage of ASCVD suggesting a direct relation between glycocalyx degradation and increased risk of atherosclerotic diseases.
**Keywords**: Endothlium, glycocalyx, atherosclerotic cardio-vascular diseases

### P.131

### Role of platelets and von Willebrand factor in pro-coagulant state in inflammatory bowel disease

**Célia Schellenberg**[1], Véronique Regnault[1], Cécile Denis[2], Peter Lenting[2], Lacolley Patrick[1], Laurent Peyrin-Biroulet[3,4], Jeremy Lagrange[1]

[1]Inserm1116, Vandoeuvre-lès-nancy, France, [2]inserm1176, Le Kremlin-Bicêtre, France, [3]inserm1256, Vandoeuvre-lès-nancy, France, [4]Department of Gastroenterology, Vandoeuvre-lès-nancy, France

**Introduction:** Inflammatory bowel disease (IBD) represents an independent risk factor for thrombosis. However, the causes of this increased risk of thrombosis are still elusive.

**Objectives**: We aim to decipher the main players in the procoagulant phenotype associated with IBD.

**Methods and results**: Coagulation phenotype assessment was performed in IBD patients included in the "I-BANK project" (CHRU Nancy), a prospective monocentric study recruiting 1000 IBD patients and in a mouse model of IBD (dextran sulphate sodium: DSS). We found an increase in platelet count in active IBD patients and an increased thrombin generation (TG) in platelet-rich plasma. Similar results were obtained in mice treated with DSS. In platelet-poor plasma, TG was not increased, highlighting the role of platelets in this phenotype. In addition, both mice and active patients showed platelet agglutination on blood smears. As circulating von Willebrand factor (VWF), which has a procoagulant function and may be involved in platelet agglutination, is elevated in IBD patients, we used VWF-deficient mice. In these mice, TG in platelet-rich plasma was not increased in response to DSS treatment. In contrast, VWF-deficient mice receiving DSS showed worsened colonic tissue damage, highlighting the importance of maintaining a normal coagulation balance in IBD.
**Conclusion**: The procoagulant phenotype in IBD depends on platelet agglutination via VWF. Further studies are needed to assess the possible beneficial effect of VWF inhibition in IBD patients at high risk of thrombosis without aggravating tissue damage.

**Keywords**: Thrombosis, Platelets, Inflammatory bowel disease

### P.132
### Differential involvement of smooth muscle cells in pro- and antithrombotic activities of abdominal versus ascending aorta aneurysms in human

**Jeremy Lagrange**[1], Mélusine Didelot[1], Véronique Olivier[2], Aurélie Ruch[1], Serguei Malikov[1,3], Patrick Lacolley[1], Jean-Baptiste Michel[1], Véronique Regnault[1]

[1]Inserm1116, Vandoeuvre-lès-nancy, France, [2]inserm1148, Paris, France, [3]Vascular surgery department CHRU Nancy, Vandœuvre-lès-Nancy, France

**Introduction:** Aneurysms of the ascending (TAA) and the abdominal aorta (AAA) share the common feature of dilation of the aorta but differ by their respective physiopathology and tissue environment in human. AAA is characterized by associated thrombosis forming an intraluminal clot, whereas thrombotic events are extremely rare in TAA, suggesting different coagulant properties between AAA and TAA.

**Objectives**: To compare coagulation capacities at tissue and cellular levels, derived from both AAA and TAA.
**Methods and results**: Human healthy aorta, AAA or TAA tissues and primary cultures of aortic smooth muscle cells (SMCs) were used. Thrombin generation was monitored by thrombography in the presence of healthy plasma. AAA tissues and SMCs have a higher ability to promote fibrin formation, to activate prothrombin, and to mobilize the tissue factor (TF) pathway, whereas TAA tissues and derived SMCs express an anti-thrombotic phenotype. Activation of the TF pathway in AAA tissue and SMCs is provoked by oxidative stress, protease-activated receptor 2 (PAR-2) overexpression and nuclear factor-kappa B (NF-κB) mobilization which could be reproduced by SMC efferocytosis of senescent red blood cells. Moreover, the high coherence between what was observed ex vivo in tissue and in passaged SMCs in vitro, demonstrated a procogulant phenotype shift in AAA SMCs, potentially as an imprinting of environmental pro-oxidative conditions of AAA.
**Conclusion**: Our data indicate that oxidative stress-induced activation of the PAR-2 – NF-κB axis and leads to an increase in TF activity and prothrombotic properties of SMCs from AAA.

**Keywords**: Vascular smooth muscl cells, Aneurysm, thrombotic properties

### P.133
### Impact of β3-adrenergic receptor modulation on vascular function during experimental septic shock

**Eugénie Hagimont**[1], Manon Durand, Antoine Kimmoun

[1]UMR_S1116 Acute and chronic cardiovascular failure, Vandoeuvre-Lès-Nancy, France

**Rationale:** Dysautonomia, an adverse event in septic shock (SC) associated with loss of cardiovascular variability, is due to an excess of catecholamines. Blockade of β1-adrenergic receptors (AR) is associated with vasoreactivity benefits however this receptor is not expressed at the vascular level (1). Unlike β1-AR, β3-ARs are widely expressed on endothelial cells and, when stimulated, cause vasorelaxation (2,3). We hypothesize that during SC, vascular function could also be mediated by β3-ARs.

**Objective**: To evaluate the impact of the modulation of β3-ARs at the level of endothelial cells on vascular function during SC.
**Methods and results**: The modulation of β3-ARs is studied in vitro with an endotoxin-induced inflammatory model on human microvascular endothelial cells (HMVECs).
First, we demonstrate that endotoxine induce an increase in the expression of endothelial surface inflammatory markers (VCAM1, ICAM1) and an activation of NFκB. Moreover, a decrease of eNOS was found, while iNOS was increased. Second, the transcriptional expression of β1 and 2-ARs decrease. At the same time, protein expression of β3-AR was unchanged. These in vitro results correlate with those found in clinical and experimental studies. Thus, we now focus on evaluating the modulation of β3-ARs.
**Conclusion**: The concept of decatecholamines emerged in order to minimize the use of catecholamines in SC. Blockade of β-AR receptors is a therapeutic approach aimed at downregulating adrenergic stimulation during SC. While β1-ARs have been widely studied, there are no data on β3-ARs. The characteristics of this AR could make it a major player in vasoreactivity.

#### References
1. Annane D, Trabold F, Sharshar T, Jarrin I, Blanc AS, Raphael JC, et al. Inappropriate sympathetic activation at onset of septic shock: a spectral analysis approach. Am J Respir Crit Care Med. août 1999;160(2):458-65.
2. Van Loon LM, van der Hoeven JG, Lemson J. Hemodynamic response to β-blockers in severe sepsis and septic shock: A review of current literature. J Crit Care. avr 2019;50:138-43.
3. Dessy C, Saliez J, Ghisdal P, Daneau G, Lobysheva II, Frérart F, et al. Endothelial beta3-adrenoreceptors mediate nitric oxide-dependent vasorelaxation of coronary microvessels in response to the third-generation beta-blocker nebivolol. Circulation. 23 août 2005;112(8):1198-205.

**Keywords**: Septic shock, β-blockers, β3-adrenergic receptor, Vascular

### P.134
### Arterial stiffness and obstruction in western Mexican healthy population and patients with type 2 diabetes, a cross-sectional study

**Erick Gonzalez Campos**[1], Luis Ricardo Balleza Alejandri[1], Fernando Grover Paez[1], Carlos Gerardo Ramos Becerra[1], David Cardona Müller[1], Ernesto German Cardona Munoz[1]

[1]Universidad De Guadalajara, Guadalajara, Mexico

**Background:** Nowadays does not exist enough studies related with cutoff points of arterial stiffness and obstruction in Mexican population compared with patients with T2DM [1]; also, is important to have a landmark on these clinical assessments to identify arterial stiffness and obstruction timely to provide also an early treatment [2–3].

**Aim**: To identify cutoff points, differences and correlations of arterial stiffness and obstruction between healthy/T2DM population.
**Methods**: In a cross-sectional study, a total of 296 western Mexican individuals (163 healthy, 133 with T2DM according with ADA 2022 [4])

were enrolled, aged 40–65 years (mean 52.7 ± 6.6). Variables like sex, BMI, baPWV and ankle-brachial index (ABI) were measured. T-student was used for equality of means and Pearson's test for correlation.
**Results**: When comparing groups (healthy/T2DM), there was not a significative difference on age (52.07 ± 6.30, 53.59 ± 0.09, p:0.052) and ABI (1.12 ± 0.07, 1.13 ± 0.13, p:0.55); we found a significative difference on BMI (25.96 ± 3.29, 29.41 ± 5.08, p: < 0.01), beats/min (62.17 ± 10.63, 70.5 ± 13.3, p: < 0.01) and baPWV (1310.31 ± 186.75, 1595.33 ± 321.99, p: < 0.01); additionally there were a significative correlation between age & baPWV (r = 0.358, p: < 0.01) and baPWV & ABI (r = 0.188, p: < 0.01).
**Conclusion**: We found a greater arterial stiffness in T2DM patients, BMI and beats/min; also, a correlation between age, ABI with arterial stiffness; and there were not differences between healthy/T2DM on ABI, and provide cutoff points for further studies.

**References**

1. Díaz-Cruz C, Patiño-Laguna ADJ, et al. Ambulatory arterial stiffness index in diabetic patients. Rev Médica Chile. 2020 Apr;148(4):496–9.
2. Gómez-Sánchez M, Patino-Alonso MC, et al. Reference values of arterial stiffness parameters and their association with cardiovascular risk factors in the Spanish population. The EVA Study. Rev Esp Cardiol. 2020 Jan;73(1):43–52.
3. Ohkuma T, Ninomiya T, et al. Brachial-Ankle Pulse Wave Velocity and the Risk Prediction of Cardiovascular Disease. Hypertension. 2017 Jun;69(6):1045–52.
4. ADA Professional Practice Committee. Classification and Diagnosis of Diabetes: Standards of Medical Care in Diabetes—2022. Diabetes Care. 2021 Dec 16;45:S17–38.

**Keywords**: Arterial stiffness, Arterial obstruction, Type 2 Diabetes Mellitus, brachial-ankle PWV

### P.136

### Characterization of an ANGPTL6 variant predisposing to intracranial aneurysm formation

**Milene Freneau**[1], Celine Baron-Menguy[1], Vincent L'Allinec[1], Marc Rio[1], Anne-Clemence Vion[1], Gervaise Loirand[1]

[1]Nantes Université, CHU Nantes, CNRS, INSERM, l'institut du thorax, F-44000 Nantes, France, Nantes, France

**Background:** Intracranial aneurysms (IA) are abnormal dilations of cerebral artery arising at bifurcations of the circle of Willis that can rupture and cause subarachnoid hemorrhage (1). By whole exome sequencing in familial IA, we identified a rare variant of the ANGPTL6 gene that predisposes to IA. This variant leads to the expression of a non-secreted truncated angiopoietin-like 6 (p.Lys460Ter-ANGPTL6) protein (2). Our aim is to understand why this variant predisposes to IA.
**Methods**: We generated Angptl6-knock in (Angptl6-KI) mice expressing the identified variant. Morphology of cerebral arteries was assessed by micro-computed tomography and confocal imaging on thick slices of adult cerebral arteries and on mouse pup retinas Functional analyses were done ex vivo on cerebral arteries and in vitro on smooth muscle cells (SMC).
**Results**: Mean diameter of linear parts of cerebral arteries was significantly larger in Angplt6-KI mice than in controls. Mutant mice also displayed hyperdensities corresponding to focal dilations and local wall deformations adjoining the center of arterial bifurcations. During retinal angiogenesis, arterial coverage by SMC was delayed in Angplt6-KI mice compared to controls. Consistently, in vitro, SMC adhered faster on ANGPTL6-coated plates than on uncoated surface. Ex vivo, cerebral arteries of mutant mice exhibited a reduced flow-mediated dilation resulting from a decreased endothelial NO production.
**Conclusions**: These data suggest that the expression of the IA-predisposing ANGPTL6 variant is sufficient to induce alterations of the cerebral arteries and impact both vascular SMC and endothelial cell functions. How these alterations favor IA formation now need to be understood.

**References**

1.Vlak MH, Algra A, Brandenburg R, Rinkel GJ. Prevalence of unruptured intracranial aneurysms, with emphasis on sex, age, comorbidity, country, and time period: a systematic review and meta-analysis. Lancet Neurol. juill 2011;10(7):626–36.
2.Bourcier R, Le Scouarnec S, Bonnaud S, Karakachoff M, Bourcereau E, Heurtebise-Chrétien S, et al. Rare Coding Variants in ANGPTL6 Are Associated with Familial Forms of Intracranial Aneurysm. Am J Hum Genet. 4 janv 2018;102(1):133-41.

**Keywords**: Angiopoietin-like 6, intracranial aneurysm, mouse

### P.138

### Chronic dopamine receptor stimulation improves endothelial function and hemodynamics in autosomal dominant polycystic kidney disease

**Audrey Dumont**[1], Mouad Hamzaoui[1], Dominique Guerrot[1], Jeremy Bellien.[1]

[1]Rouen University Hospital and INSERM U1096, Rouen, France

**Background:** Altered polycystin-mediated endothelial flow mechanosensitivity contribute to the development of hypertension and cardiovascular complications in patients with autosomal dominant polycystic kidney disease (ADPKD). Stimulation of dopamine receptors may compensate polycystin deficiency but the chronic impact of this approach has to be evaluated in patients with ADPKD.
**Methods and Results**: ADPKD patients on standard care therapy were randomized to receive during 2 months the dopamine receptor agonist rotigotine using transdermal patches at 2 mg/24 h (n = 10) and 4 mg/24 (n = 9) h or were not treated (n = 10). Rotigotine at the dose of 4 mg/24 h increased radial artery endothelium-dependent flow-mediated dilatation, measured by high-resolution echotracking, and NO release in response to hand skin heating. Systemic hemodynamics were not significantly modified but aplanation tonometry showed that rotigotine at 4 mg/24 h reduced aortic augmentation index and pulse pressure without affecting carotid-to-femoral pulse wave velocity. Plasma creatinemia and urea levels, the urinary levels of copeptin, a surrogate marker of vasopressin, and cAMP that contribute to the growth of kidney cysts in ADPKD, were not affected by rotigotine. Furthermore, chronic infusion of fenoldopam, a dopamine receptor agonist that does not cross the blood–brain barrier in contrast to rotigotine, also improved mesenteric artery flow-mediated dilatation and reduced blood pressure in mice with a specific deletion of polycystin-1 in endothelial cells.
**Conclusion**: Chronic stimulation of dopamine receptors improves conduit artery endothelial function through the increase in flow-induced NO release as well as hemodynamics in ADPKD, representing thus a promising pharmacological approach to prevent the cardiovascular complications of this disease.

**Keywords**: Endothelium, ADPKD, dopamin

### P.139

### Vascular smooth muscle cells as platelet cleaner and role of extracellular macromolecular crowding

**Rümeyza Bascetin**[1], Gabrielle Van De Velde[1], Patrick Lacolley[2], Véronique Regnault[2]

[1]Université De Lorraine, Vandœuvre-lès-nancy, France, [2]DCAC UMR s-1116, Vandœuvre-lès-nancy, France

**Background:** With aging and atherosclerosis plaque development, endothelial permeability increases leading to blood and platelets (PLT) infiltration into the vascular wall. In the media, vascular smooth muscle cells (VSMCs) are crucial for clearance of infiltrated molecules and cells including senescent red blood cells (1). Moreover, blood has a high concentration of macromolecules making it a macromolecularly crowded environment (MMC).
The objective is to decipher the clearance mechanisms of PLT by VSMCs and the influence of MMC on it.
**Methods**: Human VSMCs were cultured with either human: (i) fresh PLT, (ii) ADP-activated PLT, (iii) senescent PLT. PLT and VSMCs were stained with fluorescent tracers prior their co-culture. We also cultured VSMC in media supplemented with crowders to mimic MMC.
**Results**: After three or seven days of co-culture, we observed that activated and/or senescent PLT, which are characterized by phosphatidylserine exposure, were localized within VSMCs. In contrast to fresh red blood cells that are not phagocytosed by VSMCs, fresh PLT were also entrapped within VSMCs. We then stained VSMCs with phalloidin, an actin filament dye, revealing that PLT are surrounded by an actin shell within the VSMC. In addition, we observed that MMC modified the deposition of extracellular matrix (fibronectin, laminin and sugar moieties) by VSMCs.
**Conclusions**: VSMCs engulf PLT with an actin-dependent endocytosis process and MMC modifies the secretory phenotype of VSMC. PLT engulfment could be an inducible pathogenic event that is responsible for VSMC phenotypic switching in atherosclerosis and their procoagulant status.

**Reference**

1. Delbosc S, Bayles RG, Laschet J, Ollivier V, Ho-Tin-Noé B, Touat Z, et al. Erythrocyte Efferocytosis by the Arterial Wall Promotes Oxidation in Early-Stage Atheroma in Humans. Front Cardiovasc Med. 2017 Aug 2;4:43.

**Keywords**: Vascular smooth muscle cell, platelet, phagocytosis, macromolecular crowding

### P.140

### A single-domain antibody enhancing protein S activity reduces vaso-occlusion in a murine model of sickle cell disease

**Claire Auditeau**[1,4], Thiago Trovati Maciel[2], Josépha-Clara Sedzro[1], Camille Roussel[3,4], Aurélie Fricot-Monsinjon[3], Mariem Khamari[1], Olivier Christophe[1], Peter Lenting[1], Cécile Denis[1], François Saller[1], Delphine Borgel[1,4]

[1]Inserm U1176, Université Paris-Saclay, Le Kremlin-Bicêtre, France, [2]Institut IMAGINE INSERM U1163, Hôpital Necker Enfants Malades, Assistance Publique-Hôpitaux de Paris (AP-HP), Paris, France, [3]Biologie Intégrée du Globule Rouge, Université de Paris Cité, Université des Antilles, Paris, France, [4]Service d'Hématologie Biologique, Hôpital Necker Enfants Malades, Assistance Publique-Hôpitaux de Paris (AP-HP), Paris, France

**Background:** Protein S (PS) is a natural anticoagulant acting as a cofactor for activated protein C (APC) for the inactivation of activated factors V and VIII(1). We identified an anti-PS single-domain antibody (PS003biv) that surprisingly enhanced the APC-cofactor activity of PS and exerted an in vivo antithrombotic effect(2). A moderate decrease in PS plasma levels is frequently observed in sickle cell disease (SCD) patients, with a further reduction during vaso-occlusive crises (VOC) (3). We hypothesized that PS003biv might limit VOC in SCD.
**Methods**: HbSS-Townes mice were intravenously injected with 10 mg/kg PS003biv or vehicle (control). Mice were placed in a hypoxic atmosphere chamber (8% O2) for 3 h, after which they were returned to room air. Spleen, liver and plasma were collected. The intensity of vaso-occlusion was quantified by Ter-119 immunofluorecence staining of red blood cells (RBC) clogging the vessels in the liver and spleen. Bilirubin, free heme, and thrombin-antithrombin complexes were measured in plasma.
**Results**: Quantifying Ter-119 staining density in liver and spleen showed that PS003biv decreased RBC accumulation and VOC (liver control $13.57 \pm 1.46 \times 10^6$ versus PS003biv $7.28 \pm 0.38 \times 10^6$ AU; and spleen control $5.64 \pm 0.21 \times 10^7$ vs PS003biv $5.03 \pm 0.03 \times 10^7$ AU, $p < 0.05$). Biomarkers for hemolysis were lower in PS003biv-treated mice: bilirubin control $6.87 \pm 0.21$ vs PS003biv $4.88 \pm 0.60$ mg/dl; and free heme control $133.0 \pm 10.4$ vs PS003biv $77.5 \pm 5.3$ μM. PS003biv decreased thrombin-antithrombin complexes (control: $10.14 \pm 0.72$ vs PS003biv $7.47 \pm 0.33$ ng/mL, $p < 0.05$).
**Conclusions**: PS003biv showed beneficial properties in the context of a hypoxia/reoxygenation murine model of SCD, with reduced vaso-occlusion, hemolysis, and coagulation activation. The mechanism of action of PS003biv needs to be determined.

**References**

1. Gierula M, Ahnström J. Anticoagulant protein S-New insights on interactions and functions. J Thromb Haemost. November 2020;18(11):2801–11.
2. Sedzro JC, Adam F, Auditeau C, Bianchini E, De Carvalho A, Peyron I, et al. Antithrombotic potential of a single-domain antibody enhancing the activated protein C-cofactor activity of protein S. J Thromb Haemost. 21 April 2022;
3. Whelihan MF, Lim MY, Mooberry MJ, Piegore MG, Ilich A, Wogu A, et al. Thrombin generation and cell-dependent hypercoagulability in sickle cell disease. J Thromb Haemost. Oktober 2016;14(10):1941–52.

**Keywords**: Sickle Cell Disease, Vaso-occlusive crisis, Protein S, Single-domain antibody

### P.141

### The impact of isolated proximal limb heating on arterial wave reflection

**Chloe Athaide**[1], Jeremy Cohen[1], Kailey Stevens[1], Andrew Robertson[1,2], Jason Au[1]

[1]Department of Kinesiology and Health Sciences, University Of Waterloo, Waterloo, Canada, [2]Schlegel-University of Waterloo Research Institute for aging, University of Waterloo, Waterloo, Canada

**Background:** Arterial wave reflection occurs in the peripheral circulation subsequent to each contraction of the left ventricle (1). Mathematical models and comparative physiology (2,3) suggest that the lower limbs are the primary source of reflected waves; however, in vivo human evidence corroborating these observations is lacking. This study was designed to determine whether the lower or upper limbs contribute more to the summated reflected wave. We hypothesized that heating of the lower limb will result in larger changes in central wave reflection compared to heating of the upper limb.
**Methods**: Fifteen healthy adults (8 females, $24 \pm 3.6$ years) completed a within-subjects experimental crossover protocol with a washout period. The right arm and leg were warmed in a randomized order using 38 °C water-perfused tubing with a 30-min break between protocols. Wave reflection was estimated at baseline and after 30 min of heating from pressure-flow relationships derived from aortic blood flow and carotid blood pressure.
**Results**: There was a main effect of time for reflected wave magnitude ($12.8 \pm 2.7$ to $12.2 \pm 2.6$ mmHg) and augmentation index ($-7.49 \pm 8.92$ to $-4.45 \pm 9.07$%) ($p = 0.029$ and 0.034, respectively), but no significant differences for condition or interactions.
**Conclusion**: Proximal limb heating reduced central wave reflection magnitude; however, the lack of a difference between conditions does not support the hypothesis that the lower limbs are the primary source of wave reflection. Further research is required to confirm the role of the limbs in generating central wave reflections, with recommendations for future studies to consider the role of the gastrointestinal vasculature.

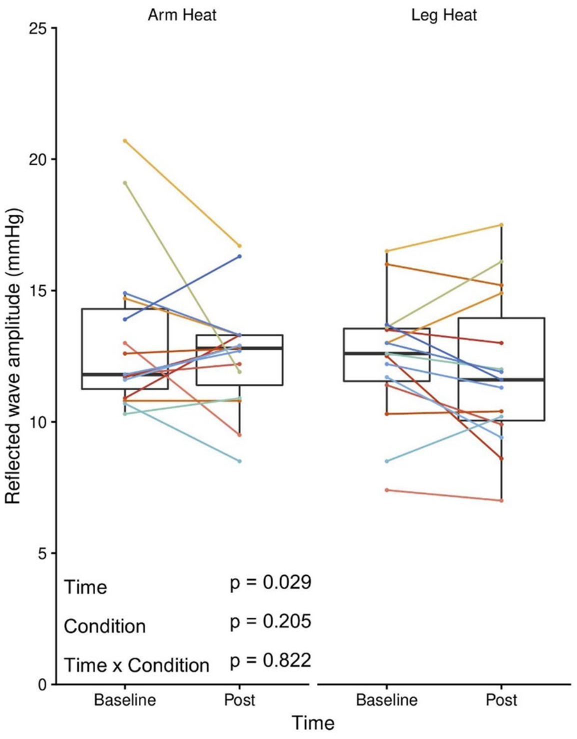

**Fig. 1** Reflected wave amplitude (mmHg) before and after 30 min of peripheral heating to the upper arm (left) and leg (right)

### References

1. Nichols WW, O'Rourke M, Vlachopoulos C. McDonald's Blood Flow in Arteries. 6th ed. McDonald's Blood Flow in Arteries. London: Hodder Arnold; 2011.
2. Avolio AP, Nichols WW, O'Rourke MF. Exaggerated wave reflection in the kangaroo simulates arterial counterpulsation. Am J Physiol—Regul Integr Comp Physiol. 1984;15(2).
3. Avolio AP, Nichols WW, O'Rourke MF. Propagation of pressure pulse in kangaroo arterial system. Am J Physiol—Regul Integr Comp Physiol. 1985;18(3):335–40.

**Keywords**: hemodynamics, heating, wave reflection

### Other

### P.151
### Development of a tool to assess knowledge and perceptions of the regulatory framework applied to medical devices for vascular ageing evaluation

**Maria Raffaella Martina**[1], Chloe Park[2], Rosa Maria Bruno[3], Areti Triantafyllou[4], Andrie G. Panayiotou[5], Dimitrios Terentes-Printzios[6], Soner Dogan[7], Marjan Manouchehri[8], Marisa Testa[9], Valentina Calderai[10], Bilge Guvenc Tuna[11], Rachel Climie[12], Christopher C. Mayer[13], Elisabetta Bianchini[1]

[1]Institute of Clinical Physiology (IFC), Italian National Research Council (CNR), Pisa, Italy, [2]University College London (UCL), London, United Kingdom, [3]Université Paris Cité, Inserm, PARCC, F-75015, Paris, France, [4]Aristotle University of Thessaloniki, Thessaloniki, Greece, [5]Cyprus University of Technology, Limassol, Cyprus, [6]First Department of Cardiology, National and Kapodistrian University of Athens, Medical School, Hippokration Hospital, Athens, Greece, [7]Yeditepe University, School of Medicine, Department of Medical Biology, Istanbul, Turkey, [8]University of Complutense; School of Pharmacy; Department of Pharmacology, Pharmacognosy and Botany, Madrid, Spain, [9]Thema srl, Imola (BO), Italy, [10]Pisa University, Pisa, Italy, [11]Yeditepe University, School of Medicine, Department of Biophysics, Istanbul, Turkey, [12]Menzies Institute for Medical Research, University of Tasmania, Hobart, Australia, [13]AIT Austrian Institute of Technology GmbH, Center for Health & Bioresources, Medical Signal Analysis, Vienna, Austria

**Background:** Regulation is part of the technology innovation process and has a key role within the lifecycle of a medical device aiming at ensuring effectiveness and safety for users (1). Ever-increasing regulatory requirements (e.g., the Regulation MDR (EU) 2017/745) impact on the development of novel and already approved systems (2, 3). Debating and raising awareness is important since it can improve the transfer of knowledge and expertise among the relevant stakeholders. Thus, the implementation of a survey to explore knowledge and perception of the regulatory framework of medical devices for the assessment of vascular age can be helpful for the involved community.
**Methods**: A multidisciplinary team including clinicians, researchers and developers from VascAgeNet was established to design and implement a digital questionnaire. A secure, open-source, two-factor authentication system for generation, distribution, and data collection of the survey supported by the European Commission (EUSurvey, https://ec.europa.eu/eusurvey) was adopted.
**Results**: The questionnaire has received ethical clearance by National Council of Research, Italy (Notification 0063984/2021) and University College London (17999/002) Research Ethics Committees and is being distributed digitally (e.g., within VascAgeNet, Artery Society etc.). The anonymous questionnaire is available at https://ec.europa.eu/eusurvey/runner/REGULATORYSurvey2022 or by scanning the QR code below, it takes approximately 10 min to complete.
**Conclusions**: A survey related to regulation and medical devices for vascular ageing assessment has been developed and is available for the community. The results could inspire concrete actions reducing gap between research and clinical practice.
The survey was developed by the COST Action CA18216, VascAgeNet supported by COST (www.cost.eu).

### References

1. Bianchini E, Mayer CC. Medical Device Regulation: Should We Care About It?. Artery Res. 2022;28:55–60. https://doi.org/10.1007/s44200-022-00014-0
2. Fraser AG, Byrne RA, Kautzner J, Butchart EG, Szymanski P, Leggeri I, et al. Implementing the new European regulations on medical devices-clinical responsibilities for evidence-based practice: A report from the regulatory affairs committee of the European society of cardiology. Eur Heart J. 2020;41(27):2589–2596. https://doi.org/10.1093/eurheartj/ehaa382

3. Mayer CC, Francesconi M, Grandi C, Mozos I, Tagliaferri S, Terentes-Printzios D, et al. Regulatory Requirements For Medical Devices And Vascular Ageing: An Overview. Hear Lung Circ. 2021;30(11):1658–66. https://doi.org/10.1016/j.hlc.2021.06.517

**Keywords**: Regulation, medical-devices, vascular-ageing, safety

### P.152

### Comparative study of diagnostic accuracy for detect peripheral artery disease among individuals with diabetes mellitus

Monique Cerqueira[1], Magno Mercês[1], **Lucélia Magalhães**[1], Amália Santana[1], Cecília Araújo[1], Daniele Brustolim[1]

[1]State University of Bahia, Salvador, Brazil

**Background:** Ankle-brachial index (ABI) is the gold standard for the noninvasive diagnosis of peripheral arterial disease (PAD), however its accuracy among people with diabetes mellitus (DM) is limited. This study aims to compare the accuracy of pulse wave velocity (PWV) measurement against ABI to diagnose PAD among individuals with DM.

**Methods**: A cross-sectional study of diagnostic accuracy will be carried out on a population of diabetics residing in Salvador, Bahia, Brazil. To evaluate the measurement of ABI, the standard technique will be used. PWV and the Augmentation Index (Aix) will be calculated using pulse tonometry with the SphygmoCor® device. Measurements in the carotid-femoral (PWVc-f) and brachial-ankle (PWVb-a) territories will be accessed. ABI values $\leq$ 0.9 mmHg or $\geq$ 1.3 mmHg, will be definers of PAD and arterial stiffness, respectively; the value of PWVc-f < 1000 cm/s and PWVb-t < 1700 cm/s will define arterial stiffness. For Aix, an increase of $\geq$ 10% will be considered significant. For the analysis of accuracy measures, sensitivity, specificity, positive predictive value and negative predictive value will be calculated, as well as the positive probability ratio and negative probability ratio of ABI and PWV, considering Doppler as the gold standard.

**Results**: It will be possible to develop some protocols and some important scientific papers, being: the prevalence of PAD among individuals with diabetes; factors associated with arterial stiffness; accuracy of PWV in diagnosing PAD.

**Conclusions**: The findings of the study will enable better attention and assistance to individuals with diabetes and PAD.

#### References

1. Boutouyrie P et al. Assessment of pulse wave velocity. Artery Res. 2009;3(1):3–8.
2. Butlin M, Qasem A. Large artery stiffness assessment using sphygmocor technology. Pulse. 2017;4(4):180–92.
3. Davies, J. H.; Lewis, J. E. A.; Williams, E. M. The Utility of Pulse Volume Waveforms in the Identification of Lower Limb Arterial Insufficiency. v. 14, n. 2, p. 5, 2014.
4. Normahani, P. et al. Study Protocol for a Comparative Diagnostic Accuracy Study of Bedside Tests Used to Detect Arterial Disease in Diabetes: TEsting for Arterial Disease in Diabetes (TrEAD) Study. BMJ Open, v. 10, n. 2, p. e033753, fev. 2020.

**Keywords**: Peripheral arterial disease, diabetes, tonometry, pulse wave analysis

### P.153

### No differences in FBN1 genotype between men with and without abdominal aortic aneurysm.

**IDA Åström Malm**[1], Peter Blomstrand[1,2], Rachel De Basso.[1]

[1]Department of Natural Science and biomedicine School of Health and welfare Jönköping University, Jönköping, Sweden, [2]Department of Clinical Physiology, County Hospital Ryhov, Jönköping, Sweden, Jönköping, Sweden

**Background:** Abdominal aortic aneurysm (AAA) is an aortic enlargement with a diameter $\geq$ 30 mm. The modifiable risk factors, such as age, male gender, and smoking, are well-known, however, there is less knowledge about the genetic factors (1–3). Fibrillin-1 (FBN1) is a protein that coordinates the deposition of elastin fibers in the extracellular matrix. Studies have found associations between the FBN1-2/3 genotype and arterial stiffness (4). Nevertheless, how FBN1 genotype, AAA, and arterial stiffness are related is less investigated. This study aims to investigate if there is a difference in FBN1 genotype between men with AAA and controls. A further aim is to study if the FBN1 genotype affects arterial wall stiffness differently in men with AAA compared to a control group.

**Methods**: Pulse wave velocity and FBN1-genotyping were performed on 229 men (159 AAA, 70 controls). The participants were recruited from ultrasound surveillance programs of known AAA or ongoing ultrasound screening programs.

**Results**: The distribution of FBN1-genotype in the AAA and control-group, were as followed, FBN1-2/2; 99 vs 45, FBN1-2/3; 13 vs 10, FBN1-2/4; 47 vs 15. However, men with AAA and FBN1-2/2 had increased central pulse wave velocity ($p < 0.005$) compared to men without AAA and FBN1-2/2 genotype.

**Conclusion**: No differences were found regarding FBN1-genotypes between men with and without AAA. Thus, the development of AAA in men seems not to be related to a specific FBN1-genotype. However, men with FBN1-2/2 and AAA have increased central arterial stiffness compared to men with the same FBN1 genotype but without AAA.

#### References

1. Wanhainen A, Bergqvist D, Boman K, Nilsson TK, Rutegard J, Bjorck M. Risk factors associated with abdominal aortic aneurysm: a population-based study with historical and current data. Journal of vascular surgery. 2005;41(3):390-6.
2. Nordon IM, Hinchliffe RJ, Loftus IM, Thompson MM. Pathophysiology and epidemiology of abdominal aortic aneurysms. Nature Reviews Cardiology. 2010;8:92.
3. Golledge J, Muller J, Daugherty A, Norman P. Abdominal Aortic Aneurysm. Arteriosclerosis, thrombosis, and vascular biology. 2006;26(12):2605-13.
4. Powell JT, Turner RJ, Sian M, Debasso R, Lanne T. Influence of fibrillin-1 genotype on the aortic stiffness in men. J Appl Physiol. 2005;99(3):1036-40.

**Keywords**: AAA, FBN1, arterial stiffness

**Author Index**

Abbaoui, Yasmine P.058
Abi-Nasr, Imad P.025, P.097
Accord, Ryan P.135
Acherar, Samir P.006
Adell, Manuel P.042
Agbulut, Onnik P.003
Agharazii, Mohsen O.10, O.17, O.5, P.045, P.058, P.065, P.092, P.093
Aizawa, Kunihiko O.19
Akbulut, Asim Cengiz P.135
Akhtar, Riaz O.9
Alastruey, Jordi O.15
Ali, Khalid P.099
Allanore, Yannick P.013
Anastasio, Fabio P.030
Argyris, Antonis P.033
Arnardottir, Hildur O.8
Arrieta, Vanessa O.21
Arroyo, Luis P.066
Artiach, Gonzalo O.8
Athaide, Chloe P.121, P.141
Au, Jason P.121, P.137, P.141
Auditeau, Claire P.140
Avolio, Alberto P.063
Avril, Stephane O.3
Ayis, Salma P.057
Aznaouridis, Konstantinos P.081
Alvarez, Virginia O.21
Astrom Malm, IDA P.153
Back, Magnus O.8
Badarienė, Jolita P.034
Badhwar, Smriti P.071
Baixauli, Vicente P.042
Balleza Alejandri, Luis Ricardo P.046, P.134
Banya, Winston P.099
Barinas-Mitchell, Emma O.18
Baron-Menguy, Celine P.136
Bascetin, Rumeyza P.139
Batta, Dora P.043
Battista, Francesca P.030
Baulmann, Johannes P.001, P.002
Becerra Ramos, Carlos Gerardo P.046
Beeckman, Simeon P.110
Bekavac, Anamarija P.105
Bellien, Jeremy O.26, P.123, P.138
Bellver, Oton P.042
Belozertseva, Ekaterina P.004
Ben Hassine, Amira O.3
Benetos, Athanase P.130
Benson, Jemima P.068
Berends, Eline P.032
Białończyk, Urszula P.070
Bianchini, Elisabetta P.014, P.030, P.151
Bidar , Elham P.135
Bikia, Vasiliki P.065
Binder, Ronald P.033
Bitsch, Nicole P.022
Blanc, Jocelyne P.003, P.004
Blomstrand, Peter P.153
Boban, Mladen P.105
Boehm, Bernhard P.037
Boleto, Goncalo P.013
Bollache, Emilie P.021
Borgel, Delphine P.140
Boutouyrie, Pierre P.013, P.025, P.065, P.106, P.110
Brett, Sally O.2
Breyer, Marie-Kathrin O.16
Breyer-Kohansal, Robab O.16
Bruno, Rosa Maria P.013, P.014, P.106, P.151
Burghuber, Otto O.16
Buschges, Julia O.13
Butlin, Mark P.063
Buyukkaya, Omer Faruk P.064
Calderai, Valentina P.151
Canales, Sergio O.22
Cardona Gutierrez, German P.046
Cardona Muller, David P.046, P.134
Cardona Munoz, Ernesto German P.134
Carracedo, Miguel O.8
Carrard, Justin P.103
Casal, Diogo P.011
Casanova, Francesco O.19
Casari, Caterina P.069
Castillo, Lidon P.042
Catalan, Marta O.22
Caulk, Alexander W.O.27
Cavinato, Cristina O.1
Centelles, Santiago P.042
Challande, Pascal P.004
Chandran, Dinu P.071, P.122
Charrier, Lise O.26
Chaturvedi, Nish P.054
Chee, Ying Jie P.038
Chen, Minghao O.1
Chesler, Naomi C.P.027
Chorda, Jose O.22, P.042
Chowienczyk, Philip J O.2
Christophe, Olivier D P.069, P.140
Cleary, Sarah O.4
Climent, Maite P.042
Climie, Rachel O.23, P.151
Cohen, Jeremy N 0.6, P.141
Colebank, Mitchel J.P.027
Coletti, Dario P.004
Colhoun, Helen M O.19
Colin, Melissa P.006
Corcillo, Antonella P.057
Corcoles, Edelmira P.042
Costa, Jose-Antonio O.22, P.042
Cote, Nadege O.5, P.058, P.093
Coulson, James P.068
Cox, James P.063
Cseprekal, Orsolya P.043
Cunha, Michelle Rabello O.25
Curcio, Rosa P.030
D'Abbondanza, Marco P.030
Dafauce Bouzo, Xela P.068
Dai, Lu P.070
Dalan, Rinkoo P.037, P.038
Danninger, Kathrin O.16, P.033
De Meyer, Guido P.127
Dębowska, Małgorzata P.070
Deepak, Kishore Kumar P.071, P.095, P.122
Delaitre, Celine P.006
Delhaas, Tammo P.022, P.061, P.067, P.128
Denis, Cecile P.069, P.131, P.140
Desbiens, Louis-Charles O.10, P.045
Di Lascio, Nicole P.014
Didelot, Melusine P.132
Dogan, Soner P.151
Dogan, Soner P.036
Dolgyras, Panagiotis P.015
Doumas, Michael O.14
Dumont, Audrey P.138
Dupuis, Francois P.006
Durand, Manon P.133
Durdabak, Dilara Buse P.036
Duval, Karine O.5
de Jaegere, Peter P.091
de Souza, Dailson N.O.17
Đogaš, Varja P.105
Eckert, Siegfried P.001, P.002

Eerik, Kadri P.044
Eha, Jaan P.044, P.055
El Omar, Reine O.7
Esparza Pimentel, Javier P.046
Eveilleau, Kornelia P.097
Faconti, Luca O.2
Farikh, Bushra O.2
Fayon, Adrien O.7
Feigerlova, Eva P.005
Fernandez-Celis, Amaya O.21
Figg, Nichola O.4
Fisher, Robert O.9
Flaquer, Maria P.057
Fortier, Catherine O.10, O.5, P.092, P.093
Foulquier, Sebastien P.006, P.022, P.032
Fountoulakis, Nikolaos P.057
Freneau, Milene P.136
Fricot-Monsinjon, Aurelie P.140
G. Schalkwijk, Casper P.032
Gainza, Alicia O.21
Galan Ruiz, Claudia Yanette P.046
Ganizada, Berta 0.11
Gao-Li, Jacqueline P.003
Garaikoetxea Zubillaga, Mattie O.21
Garcia-Pena, Amaia O.21
Garneau, Charles-Antoine O.5, P.093
Gates, Phillip E O.19
Gaucher, Caroline O.7
Gayral, Stephanie P.124
Gellert, Kapuaola O.18
Gemignani, Vincenzo P.030
Gencer, Umit P.021
Georgakopoulos, Christos P.081
Gepner, Adam P.024, P.061
Ghezzi, Pietro P.099
Giudici, Alessandro P.001, P.002, P.032, P.061, P.067, P.092, P.128
Gkaliagkousi, Eugenia O.14, P.015
Gnudi, Luigi P.057
Gomez, Ana P.042
Goncalves, Isabel O.19
Gonzalez Campos, Erick P.046, P.134
Gooding, Kim M O.19
Goudzwaard, Jeannette P.091
Goupil, Remi O.10, O.5, P.045, P.058, P.093
Gourgouli, Ioanna P.081
Gourgouli, Danai-Magdalini P.081
Greaves, Danielle K 0.6
Grillo, Andrea P.067
Grover Paez, Fernando P.046, P.134
Guerrot, Dominique P.138
Guest, Bruce P.066
Guignandon, Alain O.3
Guns, Pieter-Jan P.127
Guvenc Tuna, Bilge P.036
Gyongyosi, Helga P.043
Hagimont, Eugenie P.133
Hallab, Magid P.025, P.097
Hametner, Bernhard O.10, P.033
Hamm, Rachael O.18
Hamrouche, Marina O.26
Hamzaoui, Mouad P.138
Hanssen, Henner P.103
Hartl, Sylvia O.16
Hedge, Eric T 0.6
Heestermans, Marco P.069
Hein, Amy P.024
Helle, Deborah O.7
Henrion, Daniel P.004
Hernandez, Rosario P.042
Hinrichs, Timo P.103
Hong, Jingyuan O.15
Hope, Suzy V O.19
Hossack, Martin O.9
Houben, Boy P.051
Hughes, Alun P.053, P.054
Humphrey, Jay D.O.1, O.27
Iacob, Michaela P.123
Infanger, Denis P.103
Ioakeimidis, Nikolaos P.081
Jadoon, Maryam P.106
Jahangiri, Mohammad P.130
Jaminon, Armand 0.11
Janiak, Philip O.26
Jannot, Leo P.005
Jaryal, Ashok P.071
Jaryal, Ashok Kumar P.122
Jeroncic, Ana P.105
Jimenez, Iratxe O.22
Joannides, Robinson P.123
Johansson, Madeleine P.104
Joseph, Jayaraj P.095
Jover Garcia, Eva O.21
Junior, Edivaldo P.011
Junior, Justin P.106
Jyotsna, Viveka P P.122
Kahn, Faisel O.19
Kals, Jaak P.044, P.055, P.126
Kaniusas, Eugenijus P.033
Karadag, Cevat Volkan P.064
Karalliedde, Janaka P.057
Kasepalu, Teele P.044, P.055
Kaufmann, Christoph O.16
Keles, Nazim Arda P.036
Khettab, Hakim P.013, P.025, P.106
Kilk, Kalle P.126
Kimmoun, Antoine P.133
Kirkham, Frances Ann P.099
Klein, Marcia Regina Simas O.25
Klenk, Christopher P.103
Klosinska, Aleksandra O.4
Knaier, Raphael P.103
Koletsos, Nikolaos O.14, P.015
Konigstein, Karsten O.13, P.103
Korcarz, Claudiq P.024
Koren, Pjero P.105
Kőrosi, Beata P.043
Kroon, Bram P.051
Kuusik, Karl P.055
Labas, Carlos P.130
Lacaze, Emmanuelle P.004
Lacolley, Patrick P.003, P.004, P.130, P.132, P.139
Laffargue, Muriel P.124
Lagrange, Jeremy P.130, P.131, P.132
Laguna-Fernandez, Andres O.8
Laime, Mathilde P.025
L’Allinec, Vincent P.136
Lambrichts, Sara P.022
Lamprou, Stamatina O.14, P.015
Lamy, Jerome P.021
Lariviere, Richard O.17
Lartaud, Isabelle P.006
Laszlo, Andrea P.043
Latorre, Marcos O.1
Laučytė-Cibulskienė, Agnė P.034
Lazaridis, Antonios P.015
Le Pelletier, Laura O.12
Lecat, Sandra P.006
Lechuga, Christopher P.027
Lecoq, Enzo P.005
Lee, David O.1

Leenders, Peter P.032
Leftheriotis, Georges P.097
Lenting, Peter P.069, P.131, P.140
Li, Yanlu P.110
Li, Zhenlin P.003, P.004
Lieberg, Juri P.055
Lindholm, Bengt P.070
Liu, Xiao P.004
Loirand, Gervaise P.136
Lopez-Andres, Natalia O.21
Luo, Tao O.4
Lutsey, Pamela L O.18
Lye, David P.037
Maciel, Tamara P.121
Maciel, Thiago Trovati P.140
Mac-Way, Fabrice O.17
Madhu, Nilesh P.110
Madine, Jillian O.9
Madore, Francois O.10, P.045
Maessen, Jos 0.11
Magalhaes, Lucelia P.031
Malet, Nicole P.124
Malik, Afrah P.128
Malikov, Serguei P.132
Mangelis, Anastasios P.015, P.057
Manouchehri, Marjan P.151
Marre, Michel P.097
Martina, Maria Raffaella P.014, P.151
Martina, Mariella P.030
Martinet, Wim P.127
Martinez, Sara P.042
Martin-Nunez, Ernesto O.21
Mastrogiannis, Konstantinos O.14, P.015
Matilla Cuenca, Lara O.21
Mattace-Raso, Francesco P.091
Mattos, Samanta O.25
Maureira, Jean-Pablo O.7
Mawson, David M O.19
Mayer, Christopher O.23, P.151
Mc Cluskey, Genevieve P.069
McDonnell, Barry P.068
McNally, Ryan J O.2
Mendes, Mariana P.031
Mendizabal, Andrea O.22
Mengozzi, Manuella P.099
Mensah, Ekow P.099
Menu, Patrick O.7
Mercier, Nathalie P.130
Mess, Werner P.128
Meyer, Michelle O.18
Michel, Jean-Baptiste P.132
Mikolaitytė, Jurgita P.034
Mintziori, Gesthimani O.14
Moreau-Grange, Lucile P.123
Mousseaux, Elie P.021
Mudnic, Ivana P.105
Mulder, Paul O.26
Murtada, Sae-Il O.27
Nabeel, P. M.P.095
Nadeau-Fredette, Annie-Claire O.10, P.045
Nandi, Manasi O.15
Narang, Rajiv P.071
Natarajan, Satheesh P.062
Natour, Ehsan 0.11
Navarro, Adela O.21
Nemcsik, Janos P.043
Nemcsik-Bencze, Zsofia P.043
Neuhauser, Hannelore O.13
Neutel, Cedric P.127
Neve, Gilles P.103
Neves, Mario O.25
Newton, Michael A O.18
Nguyen, Vincent P.021
Nicol, Lionel O.26
Nikolaidou, Barbara P.015
Nilsson, Jan O.19
Nilsson, Peter M P.104
Obeid, Hasan O.5, P.025, P.065, P.092, P.093, P.097
Ofenheimer, Alina O.16
Ohlow, Marc-Alexander P.001, P.002
Okyar, Fethi P.064
Olivier, Veronique P.132
Omarjee, Loukman P.025
Orter, Stefan P.033
O’Shaughnessy, Kevin O.4
Ottas, Aigar P.044, P.126
Ozoux, Marie-Laure O.26
op ’t Roodt, Jos P.128
Paapstel, Kaido P.126
Palombo, Carlo O.19
Pan, Dan O.7
Panagiotou, Angeliki P.057
Panayiotou, Andrie G.P.151
Parati, Gianfranco P.067
Pare, Mathilde O.5, P.093
Parikh, Shaiv 0.11
Park, Chloe O.23, P.151
Parlakian, Ara P.003
Pascoe Gonzalez, Sara P.046
Patel, Chetan P.071
Patrick, Lacolley P.131
Pencheva, Margarita G.P.032
Perez, Leticia O.22
Perseguer, Zeneida P.042
Petersen, Lonnie G 0.6
Petit, Claudie O.3
Pewowaruk, Ryan P.024, P.061
Peyrin-Biroulet, Laurent P.131
Qasem, Ahmad P.063
Qureshi, Abdul P.070
Raj, Kiran V P.095 P.098, P.100, P.101, P.102, P.107, P.108
Rajkumar, Chakravarthy P.099
Ramachandra, Abhay B.O.27
Ramaekers, Mitch 0.11
Ramel, Damien P.124
Ramos Becerra, Carlos Gerardo P.134
Raoul, Alexandre P.004
Rapala, Alicja P.054
Raza, Farhan P.027
Reboucas, Laysa P.031
Reesink, Koen 0.11, P.022, P.032, P.061, P.067, P.128,
Regnault, Veronique O.7, P.003, P.004, P.130, P.131, P.132, P.139
Reig, Javier P.042
Reperant, Christelle P.069
Richard, Darren E.O.17
Richter, Stefan P.001, P.002
Rio, Marc P.136
Robertson, Andrew 0.6, P.141
Roca, Frederic P.123
Rodilla, Enrique O.22, P.042
Roldan, Alicia O.22
Roussel, Camille P.140
Royaud, Isabelle O.7
Ruch, Aurelie P.132
Ruiz, Desire P.042
Ruiz, Fanny P.042
Ruiz-Rodriguez, Maria Jesus O.1
Runciman, John P.066
Ryliškytė, Ligita P.034
Sadaba, Rafael O.21

Saez, Maria-Carmen O.22
Salar, Luis P.042
Saller, Francois P.140
Salvi, Paolo P.067
Santha Chandran, Dinu P.095
Sarthou, Marie-Kergulen P.124
Scalese, Marco P.014
Scalise, Filippo P.067
Schalkwijk, Casper P.051
Schalla, Simon 0.11
Schellenberg, Celia P.131
Schmidt-Trucksass, Arno O.13, P.103
Schurgers, Leon P.061, 0.11
Schwartz, Martin A.O.1
Sedzro, Josepha-Clara P.140
Segal, David P.097
Segers, Patrick P.065, P.110
Sharif, Isam O.4
Shore, Angela C O.19
Siew, Keith O.4
Soderberg, Magnus P.070
Soulat, Gilles P.021
Soulez, Marie Shannon P.021
Spai, Sofia P.081
Spanneut, Theo P.058
Spronck, Bart O.27, P.001, P.002, P.022, P.032, P.061, P.067, P.092, P.121, P.128
Srivastava, Prachi P.095, P.122
Stathi, Dimitra P.057
Stauber, Alexander P.001, P.002
Stehouwer, Coen P.051
Stenvinkel, Peter P.070
Stephan, Yohan O.26
Stergiopulos, Nikos P.065
Stevens, Kailey P.121, P.141
Stohr, Eric P.068
Stoner, Lee O.18
Sunjic, Borna P.105
Sutcliffe, Michael O.4
Tairi, Amira P.092, P.093
Tan, Isabella P.063
Tanaka, Hirofumi O.18
Terentes-Printzios, Dimitrios P.081, P.151
Testa, Marisa P.151
Thomas, Arthur P.130
Thomas, Mireille O.3
Tian, Lei P.004
Tisler, Andras P.043
Tomiyama, Hirofumi O.20
Tone, Caterina Maria P.004
Torella, Francesco O.9
Torop, Liisi Anette P.055
Torzsa, Peter P.043
Toupance, Simon P.130
Tousoulis, Dimitrios P.081
Triantafyllou, Areti O.14, O.23, P.015, P.151
Tsioufis, Konstantinos P.081
Tuna, Bilge P.064
Tuna, Bilge Guvenc P.151
Ung, Roth-Visal O.17
Vahi, Mare P.055
Van De Velde, Gabrielle P.139
van der Bruggen, Myrthe M.P.032
van der Laan, Koen P.022, P.032, P.128
Van Loo, Cindy P.061, P.121
van Mieghem, Nicolas P.091
Vassilenko, Valentina P.011
Vaudo, Gaetano P.030
Vicente, Julio P.042
Vico, Julieta Anabela O.21
Vion, Anne-Clemence P.136
Vlachopoulos, Charalambos P.081
Wagner, Jonathan P.103
Wahart, Amandine P.124
Wassertheurer, Siegfried O.10, P.033
Weber, Thomas O.16, O.23, P.033
Wesley, Callan P.127
Wessels, Hester P.097
Wijnhoven, Renske P.091
Wildberger, Joachim Ernst 0.11
Wilkinson, Ian O.4
Williams, Colin O.4
Wisniewski, Nathan P.005
Wymann, Matthias P.124
Yasmin, Y O.4
Young, Barnaby P.037
Zhao, Xiaofei P.051
Zilmer, Mihkel P.055
Zmuda, Louise P.123
Zografou, Ioanna O.14

Published online: 7 February 2023
